# Occupational Risk from Avian Influenza Viruses at Different Ecological Interfaces Between 1997 and 2019

**DOI:** 10.3390/microorganisms13061391

**Published:** 2025-06-14

**Authors:** Maria Alessandra De Marco, Alessandra Binazzi, Paola Melis, Claudia Cotti, Michela Bonafede, Mauro Delogu, Paola Tomao, Nicoletta Vonesch

**Affiliations:** 1 Wildlife Service, Institute for Environmental Protection and Research (ISPRA), 40064 Ozzano dell’Emilia, BO, Italy; mariaalessandra.demarco@isprambiente.it; 2 Occupational and Environmental Medicine, Epidemiology and Hygiene Department, Italian Workers’ Compensation Authority (INAIL), 00143 Rome, Italy; a.binazzi@inail.it (A.B.); m.bonafede@inail.it (M.B.); 3 Occupational and Environmental Medicine, Epidemiology and Hygiene Department, Italian Workers’ Compensation Authority (INAIL), 00078 Monte Porzio Catone, RM, Italy; p.melis@inail.it (P.M.); p.tomao@inail.it (P.T.); 4 Wildlife and Exotic Animal Service, Department of Veterinary Medical Sciences, University of Bologna, 40064 Ozzano dell’Emilia, BO, Italy; claudia.cotti@unibo.it (C.C.); mauro.delogu@unibo.it (M.D.)

**Keywords:** avian influenza, avian influenza viruses, poultry, wild birds, workplaces, exposure, bird-exposed workers, zoonotic risk, one-health approach, scoping review

## Abstract

Unprotected exposures to infected poultry or wild birds, and/or to the related avian influenza virus (AIV)-contaminated environments, could account for AIV infection in workers. This study was aimed at highlighting the ecological interfaces related to domestic poultry and wild birds posing an occupational risk regarding AIV. A search of all the articles investigating the possible presence of AIV in workers attested through virological and serological techniques and published up to August 2019 was performed on PubMed and Scopus electronic databases. Ninety-four articles consisting of 11 virological, 67 serological, and 16 mixed (both virological and serological) studies were obtained. Both virological and serological evidences of AIV infection were mainly related to H5, H7, and H9 subtypes. In addition, one piece of virological evidence for H10 subtype was reported, whereas seropositivity to all hemagglutinin subtypes from H4 to H11 was detected by serological studies. The number of AIV subtype exposures inferred from serological results showed that workers from large-scale industrial poultry farms and markets were the most represented, whereas workers from small-scale and backyard poultry farms showed seropositivity to a greater number of AIV subtypes. Workers exposed to wild bird habitats tested seropositive to H5, H9, and H11. In the occupational settings, direct contact with infected poultry or wild birds could account for AIV infection in workers. This AIV spillover can result in severe health complications for the workers, also posing a potential pandemic risk to the general population. From a public health perspective, the surveillance and early detection of AIV in workplaces should be a priority faced by a one-health approach.

## 1. Introduction

It has long been known that wild waterbirds—the natural reservoir of the influenza A virus (IAV) gene pool—perpetuate low-pathogenic (LP) avian influenza virus (AIV) subtypes in wetland habitats, as the result of a long-established balance between virus and host [1]. Indeed, these avian hosts harbor the AIV antigenic diversity provided by the reassortment of genes encoding for 16 hemagglutinins (H1 to H16) and 9 neuraminidases (N1 to N9), potentially accounting for 144 subtype combinations (H1N1 to H16N9) [2]. Among these, only the H5 and H7 antigenic subtypes can occasionally mutate in poultry into highly pathogenic (HP) AIV, posing a relevant threat to animal health worldwide [3], including the possible negative impact of the recent emergences of H5Nx HPAI viruses in wild bird and mammal populations [4].

Having eight single-stranded RNA segments, IAV is a “master in metamorphosis” characterized by the ability to rapidly acquire mutations accounting for the antigenic drift and/or shift [5]. Thus, novel IAV can emerge from wild waterbirds to infect other animals—including avian and mammalian species—showing variable degrees of viral adaptation in these new hosts, with effects spanning from sporadic infections to the possible sustained circulation of species-adapted IAV lineages as occurring in poultry, humans, pigs, horses, and dogs [6].

The interspecies transmission ability exploited by AIV accounts for real zoonotic potential, expressed by the occurrence of sporadic bird-to-human spillover events which may contribute to the genesis of new pandemic influenza strains able to spread in immunologically naïve populations [7,8,9,10].

Close contact to AIV-infected birds is recognized as a primary risk factor for the zoonotic transmission of either LP and HP virus strains [2,3,10,11], and this zoonotic risk can be markedly increased by the occupational exposure occurring in several workplaces such as farms (e.g., large-scale industrial, small-scale commercial and backyard poultry farms, ornamental/pet bird farms, and indoor and/or outdoor poultry farms), live bird markets (LBMs), poultry slaughterhouses, wildlife habitats, and diagnostic laboratories [12,13,14].

According to important reviews, several virological [15] and serological [16] studies have been put in place worldwide to assess—by a direct and indirect diagnostic approach, respectively—the zoonotic potential of AIV. However, both approaches have their pros and cons and complement each other. In fact, virological tests demonstrate a high predictive value, but the diagnostic capability is restricted to a relatively short virus-shedding period and possible mild disease in humans [15]. On the other hand, due to the longer duration of antibody detectability, serological assays offer a broader diagnostic window, but interpreting results is more challenging due to the test’s intrinsic sensitivity and specificity [16].

A scoping review was performed to assess ecological interfaces posing an occupational risk from AIV. Data from reviewed papers—categorized according to workplaces characterized by the risk of exposure to AIV at different bird–human–environment interfaces—were analyzed and discussed, focusing the attention on virological and/or serological data obtained from the different risk groups occupationally exposed to AIV, but also on the preventive measures adopted by workers as well as on the study approach underlying the papers examined. Our data emphasize how the surveillance and early detection of AIV in workplaces should be implemented as a priority by a one-health approach.

## 2. Materials and Methods

### 2.1. Search Strategy

We performed a literature search for virological and serological studies dealing with occupational exposure to AIV, with a special focus on aspects regarding the different bird–human–environment interfaces. The search and selection processes were based on the PRISMA extension for scoping reviews (PRISMA-ScR) [17] (see the Supplementary Materials for the PRISMA-ScR checklist).

The data search was performed on PubMed and Scopus electronic databases, without limiting the search start date up to August 2019.

Search strategies were formulated using the following Medical Subject Headings (MeSH) terms: occupational diseases; occupational exposure; occupations; work; Influenza; Avian; Avian Flu; Flu; Avian; Avian Influenza; Influenza A virus.

When creating the search syntax to quickly identify articles carried out in the occupational setting, we used the “more sensitive search strategy” and the strings created especially for this purpose by Mattioli et al. [18].

We created two different search strings for PubMed and Scopus, and distinct by the categories of “poultry” and “wild birds”.

#### 2.1.1. Poultry

##### PubMed

(occupational diseases [MH] OR occupational exposure [MH] OR occupational exposure* [TW] OR “occupational health” OR “occupational medicine” OR work-related OR working environment [TW] OR at work [TW] OR work environment [TW] OR occupations [MH] OR work [MH] OR workplace* [TW] OR workload OR occupation* OR worker* OR work place* [TW] OR work site* [TW] OR job* [TW] OR Veterinarian OR Laboratory technician OR Market poultry worker* OR Poultry worker* OR Poultry farm* OR Occupational poultry-exposed population OR Agricultural worker OR Bird grower* OR Chicken grower OR Slaughter* OR Abattoir* OR Culler* OR employment OR worksite* OR industry) AND (Influenza, Avian [MH] OR Avian [MH] OR Avian Flu [MH] OR Flu, Avian [MH] OR Avian Influenza [MH] OR Influenza A virus [MH] OR human Avian Influenza) AND human NOT health care worker*.

##### Scopus

(“occupational diseases” OR “occupational exposure” OR “occupational exposure*” OR “occupational health” OR “occupational medicine” OR “work related” OR “working environment” OR “at work” OR “work environment” OR “occupations” OR “work” OR “workplace*” OR “workload” OR “occupation*” OR “worke*” OR “work place*” OR “work site*” OR “job*” OR “Veterinarian” OR “Laboratory technician” OR “Market poultry worker*” OR “Poultry worker*” OR “Poultry farm*” OR “Occupational poultry exposed population” OR “Agricultural worker” OR “Bird grower*” OR “Chicken grower” OR “Slaughter*” OR “Abattoir*” OR “Culler*” OR “employment” OR “worksite*” OR “industry”) AND “avian influenza*” AND “human*” AND (LIMIT-TO (DOCTYPE, “ar”) AND (EXCLUDE (EXACTKEYWORD, “Nonhuman”) OR EXCLUDE (EXACTKEYWORD, “Health Care Personnel”) OR EXCLUDE (EXACTKEYWORD, “Clinical Trial”) OR EXCLUDE (EXACTKEYWORD, “Randomized Controlled Trial”) OR EXCLUDE (EXACTKEYWORD, “Attitude Of Health Personnel”) OR EXCLUDE (EXACTKEYWORD, “Drug Efficacy”) AND (LIMIT-TO (LANGUAGE, “English”)).

#### 2.1.2. Wild Birds

##### PubMed

(occupational diseases [MH] OR occupational exposure [MH] OR occupational exposure* [TW] OR “occupational health” OR “occupational medicine” OR work-related OR working environment [TW] OR at work [TW] OR work environment [TW] OR occupations [MH] OR work [MH] OR workplace* [TW] OR workload OR occupation* OR worke* OR work place* [TW] OR work site* [TW] OR job* [TW] OR Veterinarian OR Laboratory technician OR Wild bird market OR Wild bird trade OR Wildlife professional* OR Wildlife manager* OR Wild bird handler* OR Waterfowl hunter* OR Bird ringer* OR Bird bander* OR Zoo bird keeper* OR Sanctuary bird keeper* OR Wildlife humans contact OR Gamekeeper* OR Ranger* OR Forestry worker* OR Firemen OR Government worker* OR Personnel tasked with bird collection OR Worker* dealing with natural parks OR Captive Breeding program* OR Wild bird breeder* OR employment OR worksite* OR industry) AND (Influenza, Avian [MH] OR Avian Flu [MH] OR Flu, Avian [MH] OR Avian Influenza [MH] OR Influenza A virus [MH] OR human Avian Influenza) AND human NOT health care worker*.

##### Scopus

(“occupational diseases” OR “occupational exposure” OR “occupational exposure*” OR “occupational health” OR “occupational medicine” OR “work related” OR “working environment” OR “at work” OR “work environment” OR “occupations” OR “work” OR “workplace*” OR “workload” OR “occupation*” OR “worke*” OR “work place*” OR “work site*” OR “job*” OR “Veterinarian” OR “Laboratory technician” OR “Wild bird market” OR “Wild bird trade” OR “Wildlife professional*” OR “Wildlife manager*” OR “Wild bird handler*” OR “Waterfowl hunter*” OR “Bird ringer*” OR “Bird bander*” OR “Zoo bird keeper*” OR “Sanctuary bird keeper*” OR “Wildlife humans contact” OR “Gamekeeper*” OR “Ranger*” OR “Forestry worker*” OR “Firemen OR Government worker*” OR “Personnel tasked with bird collection” OR “Worker* dealing with natural parks” OR “Captive Breeding program*” OR “Wild bird breeder*” OR “employment” OR “worksite*” OR “industry”) AND “avian influenza*” AND “human*” AND (LIMIT-TO (DOCTYPE, “ar”)) AND (EXCLUDE (EXACTKEYWORD, “Nonhuman”) OR EXCLUDE (EXACTKEYWORD, “Health Care Personnel”) OR EXCLUDE (EXACTKEYWORD, “Clinical Trial”) OR EXCLUDE (EXACTKEYWORD, “Randomized Controlled Trial”) OR EXCLUDE (EXACTKEYWORD, “Attitude Of Health Personnel”) OR EXCLUDE (EXACTKEYWORD, “Drug Efficacy”)) AND (LIMIT-TO (LANGUAGE, “English)) AND (EXCLUDE (EXACTKEYWORD, “Drug Effect”).

### 2.2. Eligibility Criteria

Articles with the following criteria were selected and included in the present review:

- Articles published in peer-reviewed journals;
- English language;
- Publication period: no limit of years (up to August 2019);
- Observational studies (including cross-sectional, seroprevalence, retrospective, case–control, case-report);
- Occupational exposure to AIV;
- Working population: all ages, both sexes, all ethnic groups;
- Avian influenza viruses: all subtypes;
- Tests used in virological studies: AIV molecular detection and characterization (PCR, sequencing); virus isolation methods (embryonated chicken eggs, cell cultures); AIV serological characterization by haemagglutination inhibition assay (HIA), neuraminidase inhibition assay (NIA);
- Tests used in serological studies: HIA; enzyme-linked immunosorbent assay (ELISA); neutralization test (NT), microneutralization assay (MNA), plaque neutralization assay (PNA); Western blot assay (WBA); single radial haemolysis (SRH) assay; IFA, immunofluorescence assay; protein microarray;
- Only studies including tests on humans.

Non-original studies or those lacking sufficient information were excluded.

Studies that did not present original findings (reviews, letters, editorials, or comments) or those lacking sufficient information (such as details on the number of occupational cases, the methodology, virological and/or serological outcomes, and other relevant data) were eliminated. Papers assessing avian influenza viruses only in animals or environments, or solely human-to-human transmission, were also excluded.

### 2.3. Study Selection

Two pairs of researchers independently assessed the titles and abstracts of studies acquired through the search strategy.

Any disagreements were resolved through a collaborative discussion among all authors, resulting in unanimous agreement. Full-text versions of articles meeting the selection criteria were recovered and thoroughly read. Papers were examined for information on:

Country and time lapse when the study was conducted;

Workplace where the potential AIV exposure could have occurred;

Study design;

Population size and type of exposed workers;

AIV subtypes included in testing and employed methods for AIV detection;

Use of personal protective equipment (PPE) and information on vaccination and antiviral drugs (when available);

### 2.4. Synthesis

We grouped the studies by virological, serological, and mixed (both virological and serological) results, and summarized the information regarding the country/year(s) of the studies, workplace and potential AIV exposure, study design, laboratory methods and results, and preventive and protective measures. We classified work activities into 15 categories based on similar exposure risk factors, merging them into eight macro-categories according to workplaces, as follows: wildlife habitats (WLH), farms (F), markets (M), slaughterhouses (SH), laboratories (L), veterinary staff workplaces (V), places of unspecified poultry exposure (P), and other workplaces (OW) (see Table A1).

## 3. Results

### 3.1. Search Results

**Figure 1:**
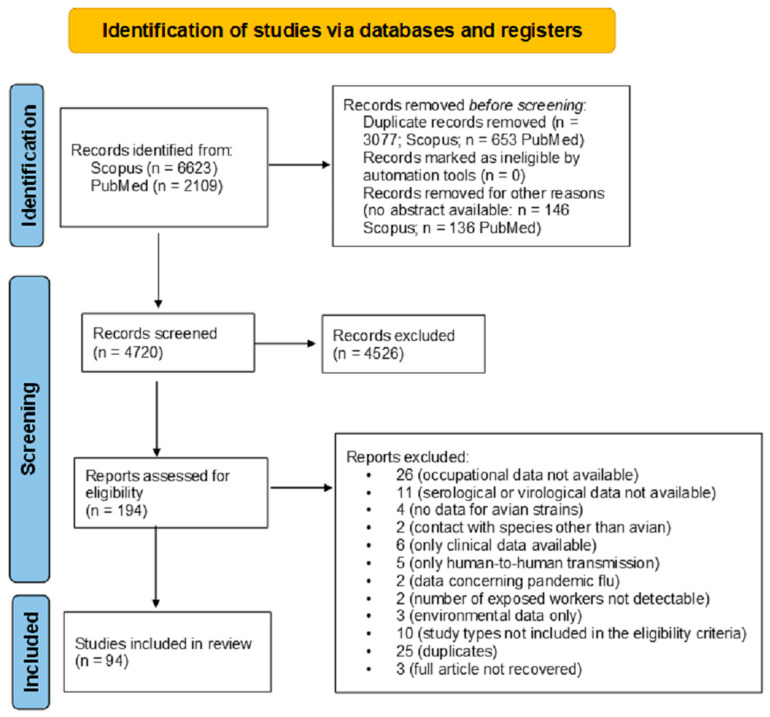
The study selection process using the PRISMA 2020 flow diagram, according to Page et al. [19].

During the selection process, a total of 6623 records were retrieved from Scopus and 2109 from PubMed, yielding 8732 articles. After removing 3733 duplicates, 5002 unique records remained, of which 282 were excluded due to the absence of an abstract. A total of 4720 abstracts were screened, with 4526 subsequently discarded. The full-text articles assessed for eligibility were 194. After the full-text screening by four authors and after subsequent discussion and consensus by other authors, 100 were excluded as they did not meet the eligibility criteria. The final output of the literature search was 94 articles (Figure 1).

**Figure 2:**
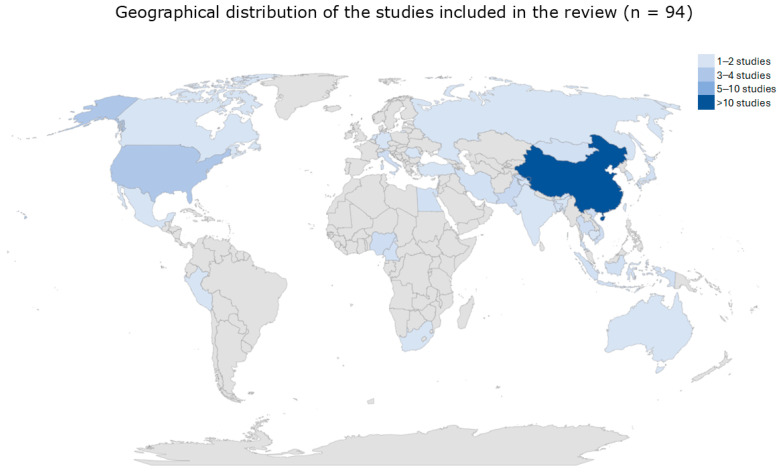
The image represents reviewed studies categorized by country and abundance.

The studies, published between 2001 and 2019, originated from all the five continents: Asia (N = 66), America (N = 12), Africa (N = 8), Europe (N = 7), and Australia N = 1). The most represented countries were China (N = 35) and USA (N = 9) (Figure 2).

**Figure 3:**
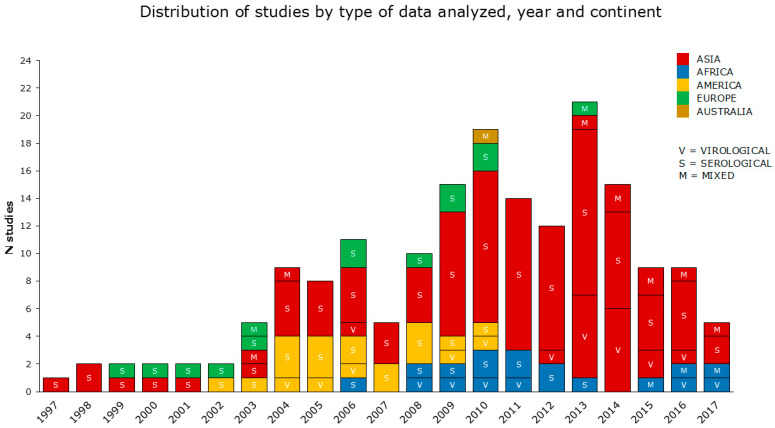
The reviewed articles aimed to assess the occupational risk posed by AIV from 1997 to 2017, visualized by representing (i) the type of data analyzed; (ii) the year(s) of the study period(s); (iii) the continent. In this graph, multiannual studies are spread over more than one year. No studies were selected in 2018 and 2019.

The periods analyzed by the reviewed studies cover a range of 23 years (from 1997 to 2019). According to the data reported, the reviewed articles were categorized into virological, serological, and mixed (both serological and virological) studies, as shown in detail in Section 2.4. The reviewed articles also included 52 multiannual studies. The greatest number of articles concerned serological data, prevalently from the Asian continent (the most condensed between 2009 and 2014), followed by America, Africa, and Europe. Virological and mixed data were investigated in a smaller number of studies, with a prevalence from Asia (Figure 3).

### 3.2. Results from the Selected Studies

#### 3.2.1. Data Overview

An overview of the main characteristics of the 94 included studies is shown in Table 1, Table 2 and Table 3. Details of the studies (country/year(s) of the studies, workplace and potential AIV exposure, study design, laboratory methods and results, preventive and protective measures) are provided in Table S1 [20,21,22,23,24,25,26,27,28,29,30], Table S2 [31,32,33,34,35,36,37,38,39,40,41,42,43,44,45,46,47,48,49,50,51,52,53,54,55,56,57,58,59,60,61,62,63,64,65,66,67,68,69,70,71,72,73,74,75,76,77,78,79,80,81,82,83,84,85,86,87,88,89,90,91,92,93,94,95,96,97,98,99,100,101,102,103,104,105,106], and Table S3 [107,108,109,110,111,112,113]. Additional country data and workplace information about the animal species and keeping system as a potential source of exposure for workers, insights into the analytic methods used in humans, and further details on the preventive measures adopted by exposed workers are available in Table S4 [20,21,22,23,24,25,26,27,28,29,30], Table S5 [31,32,33,34,35,36,37,38,39,40,41,42,43,44,45,46,47,48,49,50,51,52,53,54,55,56,57,58,59,60,61,62,63,64,65,66,67,68,69,70,71,72,73,74,75,76,77,78,79,80,81,82,83,84,85,86,87,88,89,90,91,92,93,94,95,96,97,98,99,100,101,102,103,104,105,106], and Table S6 [107,108,109,110,111,112,113]. Supplementary Tables S4–S6 also include two additional columns, aiming to check for papers reporting concurrent laboratory studies performed in animals and/or the environment according to a one-health approach and to summarize the main results from each of the reviewed studies.

#### 3.2.2. Virological Data

Table 1 and Table S1 includes 11 virological studies obtained from risk groups occupationally exposed to AIV. Six out of eleven studies were case reports, two had a cross-sectional design, and three a longitudinal design. Six studies were conducted in Asia [21,23,25,26,28,30], two in Africa [27,29], two in Europe [20,24], and one in the Americas [22]. Regarding workplaces with potential AIV exposure, seven papers focused on farms (F), one paper investigated both farms (F) and places of unspecified poultry exposure (P), and the remaining three papers focused on markets (M), places of unspecified poultry exposure (P), and both farms (F) and markets (M). To detect AIV in clinical samples, including different types of swabs and blood, 6 of 11 studies used both molecular and virus isolation methods [20,22,24,25,26,28], while five studies used molecular methods only [21,23,27,29,30]. The collected samples were tested for the presence of H5 AIV subtype in five studies [21,27,28,29,30]; in one of them [21], HPAI H5N1 virus was detected by RT-PCR in three poultry farm workers from Vietnam in the years 2003 and 2004, whereas the remaining four studies gave an H5 negative results. The presence of H7 AIV was confirmed in six of seven reviewed studies [20,22,23,24,25,26] and further characterization allowed for the identification of HP H7N7 virus in the Netherlands in 2003 [20] and in Italy in 2013 [24], as well as the HP H7N3 virus in Mexico in 2012 [22] and the LP H7N9 virus in China in 2013 [23,25,26]. The presence of multiple AIV subtypes was investigated in one study conducted in Pakistan during the 2015–2016 period [28], in which samples tested LP H9N2 positive but negative for H5 and H7 subtypes. Regarding the implementation of preventive measures, comprising the use of personal protective equipment (PPE), the administration of seasonal anti-influenza vaccine (Vax) and of antivirals (antiV), 2 papers reported the use of all three measures [20,30]; for 3 papers, no information was available [22,28,29]. The use of PPE was reported by 4 papers [20,23,24,30], the administration of seasonal anti-influenza vaccine (Vax) by 3 papers [20,27,30], and the administration of antiV by 5 papers [20,21,25,26,30]. For some of these measures, efficacy was demonstrated, since wearing PPE may have decreased the risk of bird-to-human transmission [23,24,30].

The administration of antiviral prophylactic treatment contributed to the recovery of symptomatic patients [21,25,26,30]

Further specific details on preventive measures are shown in Table S4.

#### 3.2.3. Serological Data

Table 2 and Table S2 include 67 papers [31,32,33,34,35,36,37,38,39,40,41,42,43,44,45,46,47,48,49,50,51,52,53,54,55,56,57,58,59,60,61,62,63,64,65,66,67,68,69,70,71,72,73,74,75,76,77,78,79,80,81,82,83,84,85,86,87,88,89,90,91,92,93,94,95,96,97], classified as serological studies. Forty-nine out of sixty-seven were seroprevalence studies, seven had a cross-sectional design, and eleven a longitudinal design. Forty-nine studies were conducted in Asia [31,32,34,37,41,42,43,44,45,46,49,51,53,56,59,60,62,63,64,65,67,68,69,71,72,73,74,75,77,78,79,80,81,82,83,84,85,86,87,88,89,90,91,92,93,94,95,96,97], four in Africa [48,58,70,76], five in Europe [33,50,52,61,66], and nine in the Americas [35,36,38,39,40,47,54,55,57]. According to the eight macro-categories shown in Table A1, 26 papers focused on farms (F), 6 on markets (M), 4 on wildlife habitats (WLH), and a further 4 were focused on workplaces with unspecified poultry exposure (P). Ten articles examined both farms (F) and markets (M), while six covered farms (F) and slaughterhouses (SH), and three articles regarded farms (F), slaughterhouses (SH), and markets (M). The remaining seven studies included various combinations of these workplace macro-categories. As shown in Tables S2 and S4, with regard to the serological methods, most of the reviewed studies (23 out of 67) used both HIA and MNA [32,39,41,43,48,51,59,61,69,71,73,75,76,79,81,82,84,86,87,88,89,93,95], 13 of 67 [36,40,42,44,47,55,57,58,63,66,67,68,97] and 12 of 67 [53,64,71,74,77,78,83,90,91,92,94,96] used only MNA or HIA, respectively. The 19 remaining studies used protein microarray [85] or different combinations of serological tests including HIA, MNA, NT, PNA, WBA, ELISA, IFA, SRH [31,33,34,35,37,38,45,46,49,50,52,54,56,60,62,65,70,80]. Of 67 serological studies, 31 included a group of unexposed controls [34,40,41,47,51,53,55,58,59,60,61,63,64,65,66,69,70,72,73,78,81,82,83,84,85,88,90,92,93,94,95] and only one a group of veterinarians without exposure to AIV-infected birds [40].

Statistical analysis showed higher seroprevalence (Table S2) in exposed workers than in controls in 12 studies [40,51,55,59,61,64,66,69,81,85,90,93]. In 15 out of 67 serological studies [31,35,41,44,46,49,52,65,70,86,91,92,93,94,97] workers were followed up with, and seroconversion was reported in 9 papers [31,44,65,70,86,91,92,93,94]. Regarding the implementation of preventive measures (Table S2), only 1 paper [38] reported the use of all the three measures; for 20 papers [32,33,34,41,42,45,49,51,59,60,61,64,68,71,75,79,86,87,92,94], no information was available (Table S2). The use of PPE was reported by 26 papers [31,35,36,37,38,40,43,47,48,50,52,53,54,55,56,58,62,65,67,69,72,76,77,83,89,96], the administration of seasonal anti-influenza vaccine (Vax) by 21 papers [37,38,39,40,44,50,55,56,57,58,63,66,72,76,83,85,89,91,93,95,97], and the administration of antivirals by 2 papers [38,62]. Eight articles [36,37,38,52,54,62,72,96] asserted that the use of PPE could have protected potentially exposed workers from acquiring AIV infection and be a possible explanation for negative and/or minimal serological findings. Further details on preventive measures are shown in Table S5.

#### 3.2.4. Mixed—Serological and Virological—Data

Table 3 and Table S3 comprise 16 studies [98,99,100,101,102,103,104,105,106,107,108,109,110,111,112,113], in which virological and serological analyses were performed to obtain AIV detection and seroprevalence data in occupationally exposed subjects, respectively. Three out of sixteen studies were case reports, two had a cross-sectional design, five a longitudinal design, and six were seroprevalence studies. Eleven studies were conducted in Asia [99,102,104,105,106,107,108,109,110,111,112], two in Africa [100,113], two in America [98,101], and one in Australia [103]. Regarding workplaces with potential exposure to AIV, three papers focused on farms (F) and six on markets (M), three studies reported the data of workers operating in farms (F) and markets (M), one study was conducted in wildlife habitats (WLH), and one in farms (F) and slaughterhouses (SH). The remaining two studies focused on farms (F), markets (M), slaughterhouses (SH), wildlife habitats (WLH), places of unspecified poultry exposure (P) and on farms (F), slaughterhouses (SH), wildlife habitats (WLH), respectively. Virological data in Table S3 highlight that 10 out of 16 studies used molecular methods only [100,101,102,103,104,105,108,109,110,113] and 6 studies tested samples by both molecular and virus isolation methods [98,99,106,107,111,112]. The H7N9 subtype—the most frequently searched AIV in the mixed studies [105,106,107,108,109,110,111,112]—was detected in both LBM and PF workers in China in the 2013–2015 period [106,107,108,110,111]; only one study conducted in China in 2006 [99] among six searching for HP H5N1 virus [99,100,102,104,112,113] reported positivity in one hospitalized patient. The detection of the LP H10N7 subtype was described in one SH worker in Australia in 2010 [103]. Multiple AIV subtypes were also investigated in five studies [98,100,101,102,111], in which only one H7N9 positivity already mentioned [111] was detected. With regard to the serological methods, most of the reviewed studies (8 of 16) used HIA only [104,105,106,107,108,109,111,112], 4 of 16 [98,100,101,102] MNA only, and 3 of 16 both MNA and HIA [99,110,113]. The remaining study used both HIA and VNA [103]. According to serological data from Table S3, 8 out of 16 studies included a group of unexposed controls [98,100,101,102,103,104,105,109]. Statistical analysis showed higher seroprevalence in exposed workers than in controls in one study [105]. In 7 out of 16 studies [98,100,102,103,104,109,113], workers were followed up with, and seroconversion was reported in 5 papers [98,100,104,109,113]. Regarding the use of preventive measures (Table S3), no paper reported the use of all three measures, and for 7 papers [99,100,104,106,107,112,113] no information was available. The use of PPE was reported by two papers [101,108], the administration of seasonal anti-influenza vaccine (Vax) by six papers [98,101,102,105,108,109], and the administration of antivirals by one paper [110]. No article has speculated on the efficacy of PPE in potentially AIV-exposed workers. Further details on preventive measures are shown in Table S6.

#### 3.2.5. Occupational Exposure Inferred from Serological Data

**Figure 4:**
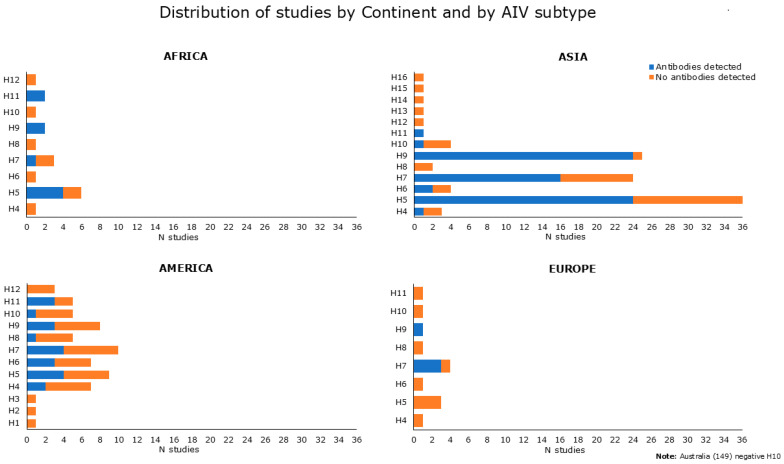
Number of reviewed studies showing positive or negative serological results (indicated in blue and in orange, respectively) extracted and categorized by continent and by AIV subtype used as an antigen in diagnostic assays. Data at baseline (not related to follow-up) were obtained from both serological and mixed studies (Tables S2 and S3).

Figure 4 summarizes and shows serological data reported in Tables S2 and S3, categorized by continent and by AIV subtype, and used as an antigen in diagnostic assays. Serological evidence for antibodies to H5, H9, and H7 avian subtypes was prevalent. Indeed, anti-H5 antibodies were reported in 32 papers, as found from Table S2 [31,34,37,40,41,44,46,49,51,55,57,58,60,63,65,71,75,76,79,83,85,86,90,91,92,93] and Table S3 [98,99,100,104,109,113]. Antibodies against H7 have been attested in 24 studies, as shown in Table S2 [33,35,40,41,52,61,74,76,78,83,85,89,90,91,92,93,95] and Table S3 [98,101,105,106,107,108,109]. Antibodies against H9 were found in 30 papers, as derived from Table S2 [34,41,53,55,58,59,63,64,66,67,68,69,73,74,77,78,81,82,84,85,86,90,91,93,94,95,96] and Table S3 [98,100,101]. Regarding the remaining HA subtype, seropositivity was found for H4 in 3 studies, 2 from Table S2 [55,72], and 1 from Table S3 [98]. Five studies found positive results for H6, as shown in Table S2 [40,55,63,83] and in Table S3 [98]. One serological study tested positive for H8 [55] and two studies reported antibodies against H10 [55,88], as exhibited in Table S2. Antibodies against H11 were found in six studies, as shown in Table S2 [39,55,58,72] and Table S3 [100,101].

#### 3.2.6. Occupational Exposure by Workplace and Work Activities

The detailed description of the distribution of AIV-positive serological results across the continents and over the years, as well as the seroconversion of exposed workers—suggestive of a local circulation of AIV in workplaces—can be found in Appendix B.1 and Appendix B.2 and in the Supplementary Materials (Tables S2 and S3).

The total number of exposures by macro-categories of workplaces at risk (Table A1) was 1514 in “Farms”, 613 in “Markets”, 447 in “Unspecified Poultry Exposure Places”, 83 in “Slaughterhouses”, 55 in “Veterinary Staff Workplaces”, 23 in “Wildlife Habitats”, 18 in “Other Workplaces”, and 15 in “Laboratories”.

**Figure 5:**
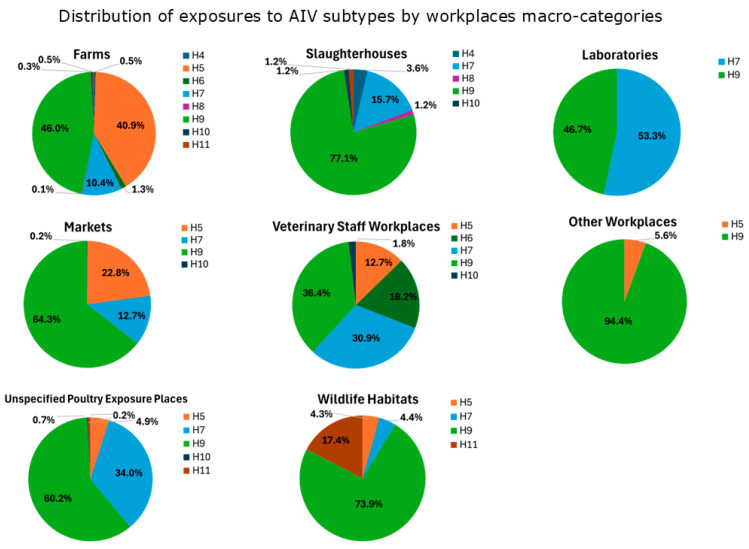
AIV subtype exposures inferred from serological results (Tables S2 and S3) categorized by workplace macro-categories at risk.

The percentage of seropositivity to avian influenza viruses in each category are reported in Figure 5, with the following distribution:

- Exposures to H9 are prevalent across all the workplaces (except for “Laboratories”). “Slaughterhouses”, “Wildlife Habitats”, “Markets” and “Unspecified Poultry Exposure Places” had the highest percentages of exposures (>60%). Studies reporting H9-positive serological results were mostly from Asia (China, Vietnam, Mongolia, Iran, India, Pakistan, and Cambodia) in a time period ranging from 2001 to 2017, but also from Africa (Nigeria) in the period 2008–2011, America (USA) between 2004 and 2010, and Europe (Romania) in the period 2009–2010 (see Appendix B.1).
- Exposures to H7 are more widespread in “Laboratories”, “Unspecified Poultry Exposure Places”, “Veterinary Staff Workplaces”, with percentages over 30%. H7-positive serological results were observed in studies from Asia (China, Pakistan, Taiwan) during a time period that ranged from 2004 to 2016, followed by America (USA) from 2002 to 2010, Europe (Italy, England) in periods between 1999 and 2010, and Africa (South Africa) in the period 2011–2012 (see Appendix B.1).
- The majority of exposures to H5 have been observed in “Farms” (40.9%), “Markets” (22.8%), and “Veterinary Staff Workplaces” (12.7%). H5-positive serological results were observed in Asian studies conducted in many countries (China, Vietnam, South Korea, Japan, Thailand, Mongolia, Bangladesh, Taiwan, Cambodia, Indonesia) in periods varying from 1997 to 2016. Also, studies originating from Africa (Nigeria, South Africa, Cameroon) reported H5-positive serological results in periods between 2008 and 2017, as well as from America (USA) in periods ranging from 2004 to 2010 (see Appendix B.1).
- Regarding other subtypes, exposures to H6 were mainly observed in “Veterinary Staff Workplaces” (18.2%), to H11 in “Wildlife Habitats” (17.4%) and to H4 in “Slaughterhouses” (3.6%). Other subtypes were less represented (H8 in “Farms” and “Slaughterhouses”, less than 1.5%; H10 in “Farms”, “Markets”, “Unspecified Poultry Exposure Places”, “Slaughterhouses”, “Veterinary Staff Workplaces”, less than 1.8%). Studies reporting positive results for these AIV subtypes were conducted in Asia and America (except for H11, which was also in Africa) (see Appendix B.1).

The richness and diversity of workers’ categories revealed by the present review was frequently associated, in the single studies, with the ranking of occupational risk by the comparison of work activities. Overall, ten of the 94 reviewed papers reported a statistically higher occupational risk as follows: for HP H7N7, in PDeW and vets vs. CoCFW and HCW [20]; for HP H5N1, in PInW vs. GoPDeW [31]; for H5, H6, and H7 AIV, in vets exposed to infected birds vs. vets without this exposure [40]; for H4, H5, H6, H8, H9, and H10 AIV, in BaTG vs. InTFW and TMePr [55]; for H4 and H11 AIV, in BaPG vs. CoPG [72]; for H7 and H9 AIV, in vaccinators vs. poultry attendants, butchers/retailers, and vets [78]; for H5 and H9, in LPMW vs. SHW [91]; for HP H5N1 in PW vs. SW [92]; for H9N2, in wholesale and retail LPMW vs. PW [95]; for H9N2, in LW vs. field vets [96] (See Table A1 and Tables S1–S6 for details).

#### 3.2.7. One-Health Approach

Overall, according to a one-health approach, 21 out of 94 reviewed papers reported concurrent laboratory studies (described in the Materials and Methods sections of the papers) performed in animals and/or the environment. This approach was implemented in 3 out of 11, in 10 out of 67, and in 8 out of 16 studies, respectively, as shown in Table 1, Table 2 and Table 3, and more in detail in Tables S4–S6.

#### 3.2.8. Study Design Comparison

To better analyze different study design approaches, particularly to avoid the possible confounding effect of study design attribution in the mixed studies (Table 3 and Table S3), we have compared different study approaches obtained from papers reporting only virological or serological results in this subsection, shown in detail in Table 1 and Table S1, Table 2 and Table S2.

According to the direct diagnostic approaches, the AIV detection results mainly relied on case reports accounting for six of the eleven studies reported in Table S1, followed by three longitudinal and two cross-sectional studies. Consequently, it should be considered that case reports—often and usefully associated with AIV detection and characterization—were mainly devoted to an in-depth investigation of a few patients suspected of AIV infection. Instead, the higher numbers of workers tested by longitudinal and cross-sectional studies could provide further epidemiological information, thus helping to better detect—by a fitting sample size—a low expected prevalence of zoonotic AIV infection, in turn characterized by a relatively short virus-shedding period and possible mild disease or asymptomatic infection in humans [15].

However, considerably more studies emerged when implementing the indirect diagnostic approaches. Indeed, during the same 1997–2019 period, a total of 67 serological studies were selected, of which forty-nine were seroprevalence studies, seven had a cross-sectional design, and eleven a longitudinal design (Table 3 and Table S3). Aiming to detect the presence of specific antibodies against AIV, these studies are limited by a higher level of difficulty when interpreting results, related to the test intrinsic sensitivity and specificity that can be improved by appropriate diagnostic methods and/or study design, such as HI assay performed with horse RBC [77]; sera tested/treated to assess/minimize possible confounding cross-reactivities against human IAV or vaccines [34,35,60,90]; co-testing sera from both AIV-exposed workers and control groups (Tables S2 and S3). On the other hand, due to the long duration of antibody detectability, serological assays offer a broader diagnostic window when compared to the virological ones and allow us to assess the AIV-infection spread in the population under study [16]. In addition, longitudinal studies following the same individuals over time can provide further relevant epidemiological data, such as the presence of seroconversion suggestive of recent AIV infection [16].

As previously stated in this review, both the direct and indirect diagnostic approaches have their pros and cons and complement each other. These intrinsic characteristics naturally accounted for the implementation of the sixteen mixed—virological and serological—studies shown in Table 3 and Table S3.

## 4. Discussion

Avian influenza viruses circulate among avian hosts but can also be transmitted to other animals such as swine, feline, equine, canine, and other mammalian species, including humans that can be infected by both HPAI and LPAI viruses [114].

In the context of the occupational health, biological risk is considered a global problem in workplaces [115] and, as is increasingly being demonstrated, the zoonotic potential of AIV spillover can represent a feared risk to occupationally exposed workers who need to be adequately protected from AIV-infected birds and/or AIV-contaminated environmental components [116,117]. According to the two search strings used in this scoping review, both the “wild birds” and “poultry” categories—with their related environments—underlie this zoonotic potential, as they include, respectively, natural reservoir species of the IAV gene pool and potential domestic reservoir or spillover hosts of AIV [118,119]. Workers’ protection stems from the risk assessment of exposure to AIV, taking into consideration the epidemiology of these viruses in domestic and wild birds, the biosafety levels and management practices in the working environments, and the protective measures adopted by workers [120]. Notably, biosafety levels are usually high in intensively reared poultry under indoor conditions, and lower in small-scale or backyard farms of birds, frequently housed partially or totally outdoors [121].

This scoping review, aimed to assess the occupational risk posed by AIV at different ecological interfaces enabling possible virus spillover [12,13,122], can be contextualized in the general history of human infections with AIV. As detailed by Wang et al. [123], from 1959 to September 2019, several AIV spillover events from poultry to humans occurred worldwide, including both HPAIV and LPAIV belonging to the H5N1, H5N6, H7N7, H7N3, H7N9, and to the H7N2, H7N3, H9N2, H7N9, H6N1, H10N7, H10N8 antigenic subtypes, respectively. Most of these zoonotic events were of limited duration, except for some epidemiological trends characterized by a wide time frame, during which human cases were reported. These long-lasting trends started with the zoonotic emergence of H5N1 HPAIV in 1997 in Asia and Africa, H9N2 LPAIV in 1998 in Asia, and LPAIV H7N9 in 2013 and HPAIV H5N6 in 2014, both in China [123]. Globally, as of 23 April 2025, 973 avian influenza A(H5N1)-confirmed human cases, including 470 deaths, have been reported [124].

As shown in Tables S2 and S3, most of the occupational categories identified in this review were seropositive to the H5 and H7 subtypes of AIV, able to circulate as LP and/or HP pathotypes [125], and to the LP H9 subtype, potentially zoonotic and blamed as a possible candidate for the next influenza pandemic in humans [126].

Regarding wildlife habitats, seropositivity to the AIV H5 subtype was found in 0.24% of US bird banders [57], whereas a seroprevalence of 0.6% was reported among bird banders to both the H7N3 and H9N2 subtypes [101]. Moreover, seropositivity to AIV H11 subtype was reported by two studies conducted in the USA [39,101], with a prevalence of 2.8% and 0.6% in duck hunters/bird banders and bird banders, respectively.

In line with the epidemiology of AIV human infections, our review attested an increased occupational risk of exposure to infected poultry during the 1997 outbreaks of HPAIV H5N1. This was demonstrated through a retrospective cohort study conducted in China among workers involved in slaughtering activities (seropositivity among exposed workers ranging from 3% to 10%) [31]. Moreover, our review evidenced H5-seroprevalences in AIV-exposed workers, ranging from 0.1% [92] to 29.1% [31], in line with other articles attesting the transmission of this virus from infected poultry to humans [127,128].

Starting from 2013, five waves of H7N9 outbreaks occurred in China, and in 2016–2017, during the fifth wave, the LPAI H7N9 virus evolved into the highly pathogenic form (HPAI H7N9), causing high mortality in poultry and human cases [123]. In our review, different categories occupationally exposed to poultry were shown to be seropositive to H7N9 in China, starting from 2012 [85], and a percentage of exposed workers was significantly higher than in the general population [105]. In our review, overall seropositivity to H7 subtypes ranged between 0.08% [41] and 21.2% [74], with data based on articles with a variety of study designs and serological tests performed.

Articles reporting H9N2-seropositive workers were mainly found in Asia, starting from 2009 in Mongolia [102]. Three studies conducted in Pakistan in the 2010–2011 period [74], in 2011 [78], and in the 2016–2017 period [96] estimated a very high H9N2 seroprevalence data among poultry workers (47.8%), vaccinators (85.7%) and different categories of poultry professionals including poultry farm workers, field veterinarians, laboratory workers (50.3%), respectively. In China, H9N2 seroprevalences ranging from 0.52% [68] to 55.6% [91] were reported in the 2009–2011 and 2013, 2014 periods. Recently, a meta-analysis targeted at evaluating the H9N2-related risk factors among humans in China, estimated an overall seroprevalence of 5.56% with significant differences among various occupationally exposed subjects [129].

In our review, we focused on the number of exposures to different H subtypes of AIV—inferred from seroprevalence data—by comparing the different macro-categories of working places rather than occupational categories. Indeed, data from AIV-exposed workers are specific to each of the studies reviewed, characterized by different patterns of exposure that can occur at the human–bird–environment interface and in turn strictly related to the epidemiological conditions at the time of enrollment. Thus, also considering the differences in the serological approaches used in the selected studies, as well as the variability of sample sizes and geographical areas, it should be noted that even the term ‘occupational exposure’, as well as sometimes the occupational groups, are inaccurately defined in the studies analyzed, making a direct comparison difficult. As detailed in the Section 3.2.6 of Results, 10 of the 94 reviewed papers reported a statistically higher occupational risk in PDeW [20], PInW [31], vets exposed to infected birds [40], BaTG [55], BaPG [72], vaccinators [78], LPMW [91], PW [92], wholesale and retail LPMW [95], LW [96] (see Table A1 and Tables S1–S6 for details).

Regarding the categories of exposed workers (Table A1), our review found that “Large scale Commercial/Industrial poultry farm workers” and “Bird-exposed market workers”, when added together, exceeded the total obtained from all the others. This is in accordance with a scoping review [117], which showed that 63.3% of the papers identified poultry farmers, breeders, and sellers as the occupational group at higher risk of exposure to AIV. It is well known that large-scale poultry farms and poultry markets are characterized by a high number of reared and commercialized birds [130] and that, in these workplaces, many are the practices exposing workers to AIV [131]. Notably, AIV detection and transmission risk to workers increased progressively along the poultry supply chain from farms, vehicles of transport, and wholesale LPM to retail LPM. [95]. Interestingly, the “Agricultural workers” and “Small scale and backyard poultry farm workers” categories—characterized by a lower number of poultry breeds, frequently housed partially or totally outdoor—showed seropositivity to a greater number of avian influenza subtypes (Tables S2 and S3), related to a possible poultry–wildlife interaction and a subsequent zoonotic risk for workers [132]. Similarly, “Veterinary staff workers” and “Slaughterhouse and poultry meat processing plant workers”—categories exposed to birds of various and multiple origins—turn out to be exposed to four different AIV subtypes each. Finally, both “Wild bird-exposed workers” and “Wild bird-exposed hunters” tested positive for H11, and this finding is not unexpected since waterfowl and shorebirds are the main reservoirs of the recognized H11 viruses [133].

In occupational settings, the use of PPE is crucial in mitigating biological risk by safeguarding the user from exposure to hazardous microorganisms. WHO recommends that workers with direct or indirect contact with AIV-infected or potentially infected poultry and wild birds or their environments should wear appropriate PPE—depending on the risk assessment specific to the task performed—primarily comprising respirators (FFP2, N95 equivalent or higher quality), eye protection (goggles or face shield), and gloves [134].

Regarding all the 94 articles included in our review, we found that PPE were used mainly in farms (F), in 22 out of 32 papers, particularly in large-scale/commercial poultry farms [20,24,31,35,36,38,47,55,56,58,72,89]. The seasonal influenza vaccine was found to have been administered mainly in farms (F), including large-scale/commercial poultry farms [20,27,38,44,55,56,58,72,89] and/or backyard poultry farms [27,38,55,58,66,72,85]. Avian H5N1 and/or seasonal influenza vaccination was reported in workers only in one paper [83]. The eleven articles reviewed affirmed that the use of PPE may have prevented workers from contracting the AIV infections, but for most of the remaining articles we were not able to evaluate their effectiveness as risk mitigation strategies. Indeed, it was not possible to systematically assess compliance with PPE use—often inconsistent and infrequent—as well as any effect of PPE by statistical methods.

WHO also recommends that hand hygiene with alcohol-based hand rub or by washing with soap and water should be performed, particularly before and after contact with animals and their environments [134]. However, few articles mentioned hand washing [48,54,58,67,96,101] and biosecurity programs adopted by farms or other premises at risk of AIV infection [47,53,72], and none provided a specific assessment about the effectiveness of these practices.

Even though its implications are significant for occupational health, the present review has several limitations, as listed below. (i) Since our study is a scoping review and not a meta-analysis, we did not systematically evaluate each study for its strengths, limitations, and biases. The main weakness is the definition of occupational categories and exposure. In fact, we have attempted to assembly work activities extrapolated from all the included publications into 15 groups based on the risk of exposure to AIVs, although we are aware that exposure depends on the specific type of practice performed, the proximity and duration of the contact with the infected/contaminated source, the size and type of the exposure source, as well as on the biosafety, protective, and preventive measures adopted, and workers provided with adequate training and information on the risks they might be exposed to. (ii) The selected studies are heterogeneous in terms of geographical distribution, study design, sample size, and recruitment. Avian influenza outbreaks may have different outcomes depending on the existing preparedness strategies and the socio-economic level of each country in which they occurred. Outbreaks in nations that implement preventive measures and adhere to surveillance and vaccination programs are frequently perceived as easily controllable: consequently, the global distribution of AIV outbreaks as reported in the scientific literature may be subjected to bias. (iii) The surveillance and diagnostic procedures applied in each country are different among the studies evaluated. This is also related to the wide period over which the articles were selected, which may have implied differences in the surveillance plans adopted, depending on the AI subtype responsible for the outbreak and its pathogenicity. Moreover, the use of standardized analytical methods would have been necessary to improve the interpretation and comparability of the laboratory data. Differences in the positivity criteria (antibody cut-off titres) used in the serological analyses have also been reported in various papers. An underestimation of seroprevalence data could be due to sampling at the wrong time, as the duration of immunity to different AIV strains is not well known. On the other hand, some seropositive workers could have unspecific antibodies against an AIV subtype because of cross-reactivity with human influenza viruses not assessed during the experimental phase. (iv) No risk of bias has been performed on the included studies: this scoping review is designed as an exploratory review of the literature, focused on the occupational risk of AIV, a topic that has not been addressed in the existing literature to the best of our knowledge. (v) Finally, another limitation is due to the use of PubMed and Scopus only: other search engines have not been utilized, as well as other data sources (agency sites, gray literature, etc.).

Despite its limitations, the present review emphasizes that AIV outbreaks occurred in both developed and developing countries, posing a significant challenge to occupational and public health. It is well known that, globally, the poultry industry represents an important and wide economic asset by producing 40% of all animal proteins in the world population [135], and we expect that further AIV outbreaks could arise following the dissemination of these viruses within and across the countries, also related to the migrations of wild birds that are able to carry and exchange AIV strains during their movements [120].

During the COVID-19 pandemic, only a few studies regarding cases of AIV infection in workers have been published. Both the efforts to fight the SARS-CoV-2 pandemic and the related mitigation measures, such as nonpharmaceutical interventions, could have caused other infectious diseases to receive less attention.

From January 2019 to June 2024, 199 laboratory-confirmed cases in the general population were reported from 17 nations, caused by five emerging (H5N8, H10N3, H3N8, H10N5, H5N2) and four re-emerging (H5N1, H5N6, H7N9, H9N2) AIV subtypes. The HPAIV detected belonged to H5 and H7 subtypes [136]. Starting from the beginning of 2022 until June 2024, 15 human infections caused by HPAI H5N1 virus clade 2.3.4.4b have been attested in six countries, and 11 of these were related to exposure to poultry [137,138,139]. Between December 2024 and March 2025, 22 new human cases of AIV were reported, including 15 A(H5) cases (12 in the USA, 2 in Cambodia, and 1 in the United Kingdom), 6 H9N2 cases, and 1 H10N3 case in China; considering the widespread circulation of AIV in animals, human infections can be considered infrequent [140].

At present, further occupational settings might be involved because of the growing number of H5 HPAIV-infected terrestrial and aquatic mammalian species [141].

Our review highlights the need of ensuring a safe workplace and implementing preventive measures, including the administration of the seasonal flu vaccine. In fact, the risk of reassortment of avian and human influenza strains, which could cause the emergence of a new pandemic strain, should not be underestimated. Moreover, the data obtained suggest that future efforts should be directed to improve AIV surveillance plans, diagnostic capacity, and biosecurity management by applying a one-health perspective [142,143]. We also found that only 21 out of 94 reviewed articles reported laboratory results performed in animals and/or their environment, whereas a one-health approach could have provided more information on workers’ exposure [144]. In fact, recognizing patterns of AIV shedding in infected poultry is essential for understanding host–pathogen interactions and adopting effective preventive and protective strategies [145]. Finally, the protection of wildlife habitats should also be considered to mitigate AIV risk [146].

In this context, the Tripartite organization, established formally in 2010 with the involvement of FAO (Food and Agriculture Organization of the United Nations), WHO (World Health Organization), and WOAH (World Organization for Animal Health), was created with the purpose of giving strategic directions for the coordination of global activities on health risks at the human–animal–environment interface. This organization has developed a guide and correlated operational tools to support countries in tackling zoonoses through a one-health approach [147,148,149].

## 5. Conclusions

An increasing risk of AIV transmission at the wildfowl–poultry–human interface occurs under ongoing conditions of global change, which include an increase in land use moving into wetlands, human population growth and life expectancy, and the need for increased food supply, provided by livestock and mostly by avian species [150]. Consequently, an increasing trend of infections with AIV has been described in different workers’ categories all over the world, including dairy cattle workers [151]. In the occupational settings, unprotected exposures to infected poultry or wild birds, and/or to the related AIV-contaminated environments, could account for AIV infection in workers. This AIV spillover can lead to illness, including severe illness, in workers, and additionally poses a potential pandemic risk for the general population. Thus, from a public health perspective, surveillance activities implemented through virological and/or serological analyses aiming toward the early detection of AIV in workplaces, supported by new technological advances such as mathematical models and artificial intelligence [152], should be a priority.

## Figures and Tables

**Table 1:** Virological studies grouped by study design, single or multiple macro-categories of workplace, and the one-health approach implementation are distributed across different continents. See Table A1, Tables S1, S4, S7 and S8 for further details.

Study Characteristics		Overall	Asia	America	Europe	Africa	References
All, n (%)		11 (100)	6 (54.5)	1 (9.1)	2 (18.2)	2 (18.2)	[20,21,22,23,24,25,26,27,28,29,30]
Study design, n (%) ^							
	Case report study	6 (54.5)	4 (66.7)	1 (100)	1 (50.0)	—	[21,22,23,24,25,26]
	Cross-sectional study	2 (18.2)	—	—	1 (50.0)	1 (50.0)	[20,29]
	Longitudinal study	3 (27.3)	2 (33.4)	—	—	1 (50.0)	[27,28,30]
Workplace macro-categories, n (% ^)							
	F	7 (63.6)	3 (50.0)	1(100)	2 (100)	1 (50.0)	[20,22,24,26,27,28,30]
	F+P	1 (9.1)	1 (16.7)	—	—	—	[21]
	M	1 (9.1)	1 (16.7)	—	—	—	[23]
	P	1 (9.1)	1 (16.7)	—	—	—	[25]
	F+M	1 (9.1)	—	—	—	1 (50.0)	[29]
One-health approach *, n (%)							
	Yes	3 (27.3)	1 (16.7)	—	—	2 (100)	[26,27,29]
	No	8 (72.7)	5 (83.3)	1 (100)	2 (100)	—	[20,21,22,23,24,25,28,30] [22] [23] [24] [25] [26]

^, % may not total 100% due to rounding; —, no study selected; F, farms; M, markets; P, workplaces of unspecified poultry exposure; *, concurrent studies performed in animals and/or environments.

**Table 2:** Serological studies grouped by study design, single or multiple macro-categories of workplace, and the one-health approach implementation are distributed across different continents. See Table A1, Tables S2, S5, S7 and S8 for further details.

Study Characteristics		Overall	Asia	America	Europe	Africa	References
All, n (%)		67 (100)	49 (73.1)	9 (13.4)	5 (7.5)	4 (6.0)	[31,32,33,34,35,36,37,38,39,40,41,42,43,44,45,46,47,48,49,50,51,52,53,54,55,56,57,58,59,60,61,62,63,64,65,66,67,68,69,70,71,72,73,74,75,76,77,78,79,80,81,82,83,84,85,86,87,88,89,90,91,92,93,94,95,96,97]
Study design, n (%) ^							
	Cross-sectional study	7 (10.4)	4 (8.2)	3 (33.3)	—		[42,54,55,57,74,75,90]
	Longitudinal study	11 (16.4)	7 (14.3)	2 (22.2)	1 (20.0)	1 (25.0)	[32,35,38,46,48,52,86,92,93,94,95]
	Seroprevalence study	49 (73.1)	37 (75.5)	4 (44.4)	4 (80.0)	3 (75.0)	[31,33,34,36,37,39,40,41,43,44,45,47,49,50,51,53,56,58,59,60,61,62,63,64,65,66,67,68,69,70,71,72,73,76,77,78,79,80,81,82,83,84,85,87,88,89,91,96,97]
Workplace macro-categories, n (%) ^							
	F	26 (38.8)	18 (36.7)	4 (44.4)	3 (60.0)	—	[33,35,36,37,38,41,42,43,44,45,46,47,49,56,60,61,63,66,69,71,72,74,77,82,83,97]
	F+M	10 (14.9)	8 (16.3)	—	—	2 (3.0)	[31,32,58,62,70,73,78,85,87,89]
	F+SH	6 (9.0)	5 (10.2)	1 (11.1)	—	—	[53,55,64,67,92,94]
	M	6 (9.0)	6 (12.2)	—	—	—	[34,59,65,79,86,93]
	WLH	4 (6.0)	—	3 (33.3)	1 (20.0)	—	[39,50,54,57]
	P	4 (6.0)	2 (4.0)	1 (11.1)	1 (20.0)	—	[40,51,52,80]
	F+SH+M	3 (4.5)	3 (6.1)	—	—	—	[75,81,95]
	F+M+V	1 (1.5)	1 (2.0)	—	—	—	[90]
	F+V+SH+M	1 (1.5)	1 (2.0)	—	—	—	[88]
	F+M+L	1 (1.5)	—	—	—	1 (1.5)	[48]
	F+SH+V	1 (1.5)	1 (2.0)	—	—	—	[84]
	M+F+SH+WLH	1 (1.5)	1 (2.0)	—	—	—	[68]
	M+SH	1 (1.5)	1 (2.0)	—	—	—	[91]
	P+SH+L	1 (1.5)	1 (2.0)	—	—	—	[96]
	OW	1 (1.5)	—	—	1 (20.0)	—	[76]
One-health approach *, n (%)							
	Yes	10 (14.9)	9 (18.4)	—	—	1 (25.0)	[32,43,48,59,60,65,75,86,93,95]
	No	57 (85.1)	40 (81.6)	9 (100)	5 (100)	3 (75.0)	[31,33,34,35,36,37,38,39,40,41,42,44,45,46,47,49,50,51,52,53,54,55,56,57,58,61,62,63,64,66,67,68,69,70,71,72,73,74,76,77,78,79,80,81,82,83,84,85,87,88,89,90,91,92,94,95,96,97]

^, % may not total 100% due to rounding; —, no study selected; F, farms; M, markets; SH, slaughterhouses; WLH, wildlife habitat; P, workplaces of unspecified poultry exposure; V, veterinary staff workplaces; L, laboratories; OW, other workplaces; *, concurrent studies performed in animals and/or environments.

**Table 3:** Mixed—serological and virological—studies grouped by study design, single or multiple macro-categories of workplace, and the one-health approach implementation are distributed across different continents. See Table A1, Tables S3 and S6–S8 for further details.

Study Characteristics		Overall	Asia	America	Europe	Africa	Australia	References
All, n (%)		16 (100)	11 (68.7)	2 (12.5)	—	2 (12.5)	1 (6.3)	[98,99,100,101,102,103,104,105,106,107,108,109,110,111,112,113]
Study design, n (%) ^							—	
	Case report study	3 (18.7)	2 (18.2)	—	—	—	1 (100)	[99,103,106]
	Cross-sectional study	2 (12.5)	1 (9.1)	1 (50.0)	—	—	—	[101,107]
	Longitudinal study	5 (31.2)	2 (18.2)	1 (50.0)	—	2 (100)	—	[98,100,102,110,113]
	Seroprevalence study	6 (37.5)	6 (54.5)	—	—	—	—	[104,105,108,109,111,112]
Workplace macro-categories, n (%) ^							—	—
	F	3 (18.7)	3 (27.3)	—	—	—	—	[102,111,112]
	F+M	3 (18.7)	1 (9.1)	—	—	2 (100)	—	[100,108,113]
	M	6 (37.5)	6 (54.5)	—	—	—	—	[99,104,105,106,109,110]
	F+SH	1 (6.3)	—	—	—	—	1 (100)	[103]
	WLH	1 (6.3)	—	1 (50.0)	—	—	—	[101]
	F+M+SH+WLH+P	1 (6.3)	1 (9.1)	—	—	—	—	[107]
	F+SH+WLH	1 (6.3)	—	1 (50.0)	—	—	—	[98]
One-health approach *, n (%)								
	Yes	8 (50.0)	6 (54.5)	—	—	1 (50.0)	1 (100)	[99,103,104,107,108,110,111,113]
	No	8 (50.0)	5 (45,5)	2 (100)	—	1 (50,0)	—	[98,100,101,102,105,106,109,112]

^, % may not total 100% due to rounding; —, no study selected; F, farms; M, markets; SH, slaughterhouses; WLH, wildlife habitat; P, workplaces of unspecified poultry exposure; *, concurrent studies performed in animals and/or environments.

## Data Availability

The original contributions presented in the study are included in the article/Supplementary Material, further inquiries can be directed to the corresponding author.

## Supplementary Materials

The following supporting information can be downloaded at: https://www.mdpi.com/article/10.3390/microorganisms13061391/s1, PRISMA-ScR (Preferred Reporting Items for Systematic reviews and Meta Analyses extension for Scoping reviews) Checklist. Table S1. Virological results obtained from risk groups occupationally exposed to AIV. See Table footer, Table A1, Tables S4, S7 and S8 for acronyms and/or further details. Table S2. Serological results obtained from risk groups occupationally exposed to AIV. See Table footer, Table A1, Tables S5, S7 and S8 for acronyms and/or further details. Table S3. Mixed—virological and serological—results obtained from risk groups occupationally exposed to AIV. See Table footer, Table A1, Tables S6–S8 for acronyms and/or further details. Table S4. Additional information related to Table S1 (virological results obtained from risk groups occupationally exposed to AIV). See Tables S7 and S8 for acronym details. Table S5. Additional information related to Table S2 (serological results obtained from risk groups occupationally exposed to AIV). See Tables S7 and S8 for acronym details. Table S6. Additional information related to Table S3 (mixed—virological and serological—results obtained from risk groups occupationally exposed to AIV). See Tables S7 and S8 for acronym details. Table S7. Ecological interfaces posing zoonotic occupational risk: workplaces and risk-group acronyms used in Tables S1–S3. Table S8. Ecological interfaces posing zoonotic occupational risk: list of additional acronyms used in Tables S1–S3.

## Appendix A

**Table A1:** Occupational zoonotic risk posed by avian influenza viruses: work activities and workplaces related to different human–bird–environment interfaces.

Workplace Macro-Categories Grouping Work Activities (n = 8)	Work Activities (n = 15)	Worker Acronym	Acronym Detail
WLH (Wildlife Habitats)	Wild Bird-Exposed Worker(s)	BBa	bird bander(s)
		BHa	bird handler(s)
		WBHW	wild bird habitat worker(s)
		WLB	wildlife biologist(s)
		WLE	wildlife exposed
		WLE-GoW	wildlife exposed government worker(s)
	Wild Bird-Exposed Hunter(s)	BHu	bird hunter(s)
		DHu	duck hunter(s)
		RSBu	rural subsistence bird hunter
		USpHu	urban sport hunter(s)
		WBHu	wild bird hunter(s)
F (Farms)	Large-Scale Commercial/Industrial Poultry Farm Worker(s)	CoCFW	commercial chicken farm worker(s)
		CoPFW	commercial poultry farm worker(s)
		CoPG	commercial poultry grower(s)
		InPFW	industrial poultry farm worker(s)
		InTFW	industrial turkey farm worker(s)
		Large-scale PFW	large-scale poultry farm worker(s)
		PInW	poultry industry worker(s)
	Agricultural Worker(s)	AgW	agricultural worker(s)
		AnFW	animal farm worker(s)
		FW	farm worker(s)
	Small-Scale and Backyard Poultry Farm Worker(s)	BaPFW	backyard poultry farm worker(s)
		BaPG	backyard poultry grower(s)
		BaTG	backyard turkey grower(s)
		Small-scale PFW	small-scale poultry farm worker(s)
	Poultry Depopulation Worker(s)	DeW	depopulation worker(s)
		GoPDeW	government poultry depopulation worker(s)
		GoW	government worker(s)
		PCu	poultry culler(s)
		PDeW	poultry depopulation worker(s)
	Swine Farm Worker(s)	SFW	swine farm worker(s)
		CoSFW	commercial swine farm worker(s)
	Poultry Farm Worker(s) *	DFW	duck farm worker(s)
		PFW	poultry farm worker(s)
		PG	poultry grower(s)
M (Markets)	Bird-Exposed Market Worker(s)	LBMW	live bird market worker(s)
		LPM butcher	live poultry market butcher(s)
		LPMW	live poultry market worker(s)
		LPV	live poultry vendor(s)
		PMW	poultry market worker(s)
		Wholesale market seller	wholesale market seller(s)
		WMW	wet marker worker(s)
SH (Slaughterhouses)	Slaughterhouse and Poultry Meat-Processing Plant Worker(s)	SHW	slaughterhouse worker(s)
		PMePr	poultry meat processor(s)
L (Laboratories)	Laboratory Worker(s)	LW	laboratory worker(s)
V (Veterinary Staff Workplaces)	Veterinary Staff Worker(s)	Vaccinators	—
		Vet	veterinarian(s)
		Veterinary staff	—
P (Unspecified Poultry Exposure Places)	Poultry Worker(s)	PW	poultry worker(s)
	(Live/Dead) Poultry Exposed Worker(s) **	WpE(L/D)PW	worker(s) in workplace(s) involving exposure to (live/dead) poultry
OW (Other Workplaces)	Other Worker(s)	OW	other worker(s)

*, poultry workers employed on farms of unspecified size; **, poultry workers with exposure to live or dead poultry. Fifteen work activities based on similar exposure risk factors were merged into eight macro-categories of workplaces grouped by different color backgrounds as follows: green, wildlife habitats (WLH); pink, farms (F); yellow, markets (M); light blue, slaughterhouses (SH); gray, laboratories (L); white, veterinary staff workplaces (V), places of unspecified poultry exposure (P), and other workplaces (OW).

## Appendix B

### Appendix B.1. Distribution of AIV Positive Serological Results

The distribution of AIV-positive serological results across the continents and over the years, also shown in Tables S2 and S3, is described below.

In Africa, AIV seropositivity was reported as follows. In Africa, H11, H9, and H5 were reported in Nigeria [58,100] in the 2008–2011 period; H7 and H5 in South Africa [76] in the 2011–2012 period; and H5 in Cameroon [113] in the 2016–2017 period.

In Asia, AIV seropositivity was found as follows: H11 was reported in Lebanon [72] in 2010; H10 in China [88] in 2013; H9 in Vietnam [34] in 2001, in China in 2004 [41], in 2006 and 2008 [53], in the 2008–2010 period [59], between 2009 and 2013 [67,68,69,77,81,82] and between 2012 and 2016 [85,90,91,93,94,95], in Mongolia [63] in 2009, in Iran in 2009 [64] and in the 2012–2013 period [84], in India [73] in 2010, in Pakistan in the 2010–2011 period [74,78] and in the 2016–2017 period [96], and in Cambodia [86] in 2013; H7 in China in 2004 [41], in 2012, 2013, 2014 [85], in 2013 [105,106,109], between 2013 and 2014 [89,90,91,108], in the 2013–2015 period [92,93,107] and in 2015–2016 [95], in Pakistan [74,78] between 2010 and 2011, in Taiwan [83] in 2012; H6 in Mongolia [63] in 2009, in Taiwan [83] in 2012; H5 in China in the 1997–1998 period [31], in 2004 and in 2006 [41,99], between 2008 and 2012 [60,71,75], between 2012 and 2014 [85,90,91], between 2013 and 2016 [92,93,95,109], in Vietnam in 2001 [34] and in 2011 [79], in South Korea [37] in 2003–2004, in Japan in 2005 [44] and in 2006 [51], in Thailand [46] in the 2005–2008 period, in Mongolia [63] in 2009, in Bangladesh [65] in the 2009–2010 period, in Taiwan [83] in 2012, in Cambodia [86] in 2013, in Indonesia [104] in the 2012–2016 period; H4 in Lebanon [72] in 2010.

In the Americas, AIV seropositivity was reported as follows: H11 in USA in 2004, 2005 [39] and in the 2007–2008 period [55]; H10 in USA [55] in the 2007–2008 period; H9 in USA in 2004, 2006 [98], in the 2007–2008 period [55], in 2009, 2010 [101]; H8 in USA [55] in the 2007–2008 period; H7 in USA in 2002 [35], in 2004, 2006 [40,98], in 2009, 2010 [101]; H6 in USA in 2004, 2006 [40,98] and in the 2007–2008 period [55]; H5 in USA in 2004, 2006 [40,98], in the 2007–2008 period [55], in 2008, 2009, 2010 [57]; H4 in USA in the 2007–2008 period [55], in 2004, 2006 [98].

In Europe, AIV seropositivity was reported as follows: H9 was reported in Romania [66] in the 2009–2010 period; H7 in Italy in the 1999–2003 [33] and in the 2008–2010 [61] periods, and in England [52] in 2006.

### Appendix B.2. Seroconversion Results in AIV Exposed Workers

As shown in Tables S2 and S3, the seroconversion of exposed workers, suggestive of local circulation of AIV in workplaces, was reported as follows: in China [31], in the 1997–1998 period, 1 s.c. for H5; in Japan [44], in 2005, 20 s.c. for H5; in Bangladesh [65], in the 2009–2010 period, 6 s.c. for H5; in Egypt [70], in the 2010–2013 period, 27 s.c. for H7 with workers’ sera negative at baseline; in Cambodia [86], in 2013, 4 s.c. for H5, 1 s.c. for H9; in China [91], in 2013, 2014, 7 s.c. for H5, 2 s.c. for H7, 7 s.c. for H9; in China [92], in the 2013–2015 period, 16 s.c. for H7N9, 42 s.c. for H5N1 clade 2.3.4, 5 s.c. for H5N1 clade 2.3.2.1; in China [93], in the 2013–2016 period, 2 s.c. for H7, 13 s.c. for H9, 7 s.c. for H5; in China [94], in the 2013–2016 period, 96 s.c. for H9; in USA [98], in 2004, 2006, 6 s.c. for H5, 2 s.c. for H9; in Nigeria [100] in the 2008–2011 period, 5 s.c. for H5N1 with workers’ sera negative at baseline; in Indonesia [104] in the 2012–2016 period, 15 s.c. for H5; in China [109] in 2013, 52 s.c. for H7N9; in Cameroon [113] in the 2016–2017 period, 2 s.c. for H5.
