# Supplementary material for: Occupational Risk from Avian Influenza Viruses at Different Ecological Interfaces Between 1997 and 2019
Source: Microorganisms. 2025 Jun 14;13(6):1391. doi: 10.3390/microorganisms13061391 (PMC12195780; doi:10.3390/microorganisms13061391)
Supplement: Supplementary file 1 [file microorganisms-13-01391-s001.zip › Table S1.pdf]

**Table S1.** Virological results obtained from risk groups occupationally exposed to AIV. See Table footer, Table A1, Table S4, Table S7, and Table S8 for acronyms and/or further details.

| Ref. | Country, Year(s) | Workplace / potential AIV exposure | Study Design | AIV detection and further characterization (pos./tested)# | PPE | Vax | antiV |
|---|---|---|---|---|---|---|---|
| [20] | Netherlands, 2003 | CoCF / HP H7N7 | Cross-sectional | H7 (89/453: 16 CoCFW and their FaM, 54 **PDeW**, 5 **Vet**, 0 HCW, 14 OW e.sw., np.sw./op.sw.) by RT-PCR (25/89 initially confirmed by VI-CC/antigenic subtyping). H7 (31/39 e.sw.) by VI-CC, molecular methods. | Yes | Yes | Yes |
| [21] | Vietnam, 2003, 2004 | PF, WpE(L/D)P / HP H5N1 | Case report | H5, N1 (3 PFW /10 infected patients n.sw./t.sw.) by RT-PCR. | n.i. | n.i. | Yes |
| [22] | Mexico, 2012 | PF / HP H7N3 | Case report | H7 (2/2 PFW c.sw.) by rRT-PCR. HP H7N3 (1/1 PFW c.sw.) by VI-EE, genomic and antigenic analyses. | n.i. | n.i. | n.i. |
| [23] | China, 2013 | Wholesale WM / H7N9 | Case report | H7N9 (1/61 PDeW p.sw./s.sw.) by rRT-PCR. | Yes | n.i. | n.i. |
| [24] | Italy, 2013 | InPF / HP H7N7 | Case report | H7N7 (3/3 InPFW/PDeW c.sw.) by rRT-PCR. HP H7N7 (1/3 InPFW/PDeW c.sw.) by VI-CC, genomic and antigenic analyses. | Yes | n.i. | n.i. |
| [25] | China, 2014 | WpE(L/D)P / H7N9 | Case report | H7N9 (1/1 WpE(L/D)PW op.sw.) by VI-EE, VI-CC, rRT-PCR. H7N9 (1/1 WpE(L/D)PW op.sw.) by genetic and phylogenetic analyses | n.i. | n.i. | Yes |
| [26] | China 2014 | Small-scale PF / H7N9 | Case report | H7N9 (1/1 PW t.sw.) by rRT-PCR, VI-EE, genomic analyses | No | n.i. | Yes |
| [27] | Egypt, 2015 | CoPF, BaPF / H5 AI | Longitudinal | H5 (0/50 CoPFW and BaPFW, 0/15 HS n.sw/op.sw.) by rRT-PCR. | n.i. | Yes | n.i. |
| [28] | Pakistan, 2015-2016 | BaPF, CoPF / H9N2 | Longitudinal | H9 (1/117 BaPFW n.sw), H5 and H7 (0/117 CoPFW and BaPFW n.sw.) by VI-EE, HAA, HIA. H9N2 (1/1 PW n.sw.) by RT-PCR, genetic and phylogenetic analyses. | n.i. | n.i. | n.i. |
| [29] | Cameroon, 2016-2017 | CoPF, BaPF, LBM / HP H5N1, H5N8 | Cross-sectional | HPAIV (0/481, of which 0/136 retested, CoPFW, BaPFW and LBMW blood samples) by RT-PCR | n.i. | n.i. | n.i. |
| [30] | Korea, 2017 | PF / HP H5N6 | Longitudinal | H5 (0/22 PDeW np.sw.) by rRT-PCR | Yes | Yes | Yes |

#, in red bold font statistically higher occupational risk in workers; PPE, personal protective equipment; Vax, seasonal influenza vaccination; antiV, antiviral prophylaxis and/or therapy; n.i., no information
