# Supplementary material for: Occupational Risk from Avian Influenza Viruses at Different Ecological Interfaces Between 1997 and 2019
Source: Microorganisms. 2025 Jun 14;13(6):1391. doi: 10.3390/microorganisms13061391 (PMC12195780; doi:10.3390/microorganisms13061391)
Supplement: Supplementary file 1 [file microorganisms-13-01391-s001.zip › Table S2.pdf]

Table S2. Serological results obtained from risk groups occupationally exposed to AIV. See Table footer, Table A1, Table S5, Table S7, and Table S8 for acronyms and/or further details.

Ref.	Country, Year(s)	Workplace / potential AIV exposure	Study Design)	HA sutype(s) of AIV Ag	Seroprevalence % (pos./tested)#, ## [Follow-up (pos./tested), seroconversion]	PPE	Vax	antiV
[31]	China, 1997-1998	Retail and wholesale PM, hatchery, PF and other PIn / HP H5N1	Seroprevalence	H5, H5§	Overall, 29.1% (444/1525 PInW) and 3% (9/293 GoPDeW) by MNA (partially confirmed by WBA in 81/231 PInW, 9/9 GoPDeW). [229/293 GoPDeW, 1 s.c.]	Yes	n.i.	n.i.
[32]	Taiwan, 1998-2000	Wholesale and retail PM, PF / H3, H4, H6 AI	Longitudinal	H4, H6	0/195 PMW and PFW, 0/146 Vet for H4 and H6 by HIA and MNA.	n.i.	n.i.	n.i.
[33]	Italy, 1999-2003	InPF / LP and HP H7N1and LP H7N3	Seroprevalence	H7	0/798 InPFW exposed in 1999-2002 to H7N1 and H7N3 by MNA, HIA, SRH; 3.8% (7/185) InPFW exposed in 2003 to H7N1 and H7N3 by MNA, 7/7 confirmed by WBA.	n.i.	n.i.	n.i.
[34]	Vietnam, 2001	Large LPM / HPAI or LPAI H5 or LPAI H9 virus	Seroprevalence (c=200)	H5, H5§, H9	4% and 3% (8/200 and 6/200) LPMW for H5 and H9 respectively by MNA confirmed by WBA.	n.i.	n.i.	n.i.
[35]	USA, 2002	CoPF / LP H7N2	Longitudinal	H7	1.25% (1/80 GoW involved in control outbreak activities) by MNA confirmed by WBA, ELISA. [1/80 (1/1), no s.c.]	Yes	n.i.	n.i.
[36]	USA, 2003, 2005	Large PIn / AIV	Seroprevalence	H4-H7, H9	0/24 PInW for H4-H7, H9, by MNA.	Yes	n.i.	n.i.
[37]	South Korea, 2003-2004	PF / HP H5N1	Seroprevalence	H5	0.68% (9/1327 PCu) 0/176, PFW, 0/70 OW by MNA confirmed by WBA.	Yes	Yes	n.i.
[38]	Canada, 2004	BaPF, CoPF / HP H7N3	Longitudinal	H7§	0/167 PDeW, Vet, OW by MNA of which 0/16 confirmed by HIA, WB.	Yes	Yes	Yes
[39]	USA, 2004, 2005	Wetland WLH / AIV	Seroprevalence	H1-H12	2.80% (3/107, of which 1/68 DHu, 2/39 BBa) by MNA and HIA for H11; 0/68 DHu by MNA for H1-H10, H12 and 0/39 BBa by MNA for H1, H3-H10, H12.	No	Yes	n.i.
[40]	USA, 2004, 2006	WpE(L/D)P / AIV	Seroprevalence (c=66)	H4-H12	Of 42 Vet, **12.2%** (5/41), **23.8%** (10/42), **14.4%** (6/41) for **H5**, **H6**, **H7** respectively, and 0/42 for H8 by MNA. Statistically not significant results by MNA for H4, H9-H12.	Yes	Yes	n.i.
[41]	China, 2004	Small-scale outdoor PF / HP H5N1	Seroprevalence (c=983)	H5, H7, H9	3.03%, (7/231**),** **9.52**% (22/231) and 0.43% (1/231) for H5, H9 and H7 respectively, in PG, LPV, PCu by HIA (randomly confirmed by MNA). [7/231 (1/7 for H5), 22/231 (0/22) for H7), 1/231 (0/1 for H9), no s.c.]	n.i.	n.i.	n.i.

[42]	Thailand, 2004	InPF, BaPF / HP H5N1	Cross-sectional	H5	0/322 InPFW, BaPFW and PCu by MNA. 7/322 PFW had low (<1:80) Ab titers.	n.i.	n.i.	n.i.
[43]	Vietnam 2004-2005	PF / HP H5N1	Seroprevalence	H5§	0/500 PFW, PCu by MNA. 3/317 PCu had low (1:80) Ab titers y HIA.	Yes	n.i.	n.i.
[44]	Japan, 2005	Large CoPF / LP H5N2	Seroprevalence	H5	14.0% (36/257 CoPFW) by MNA. [257/257 (48/257), 20 s.c.]	n.i.	Yes	n.i.
[45]	Thailand, 2005	BaPF, AgA, rural villages / HP H5N1	Seroprevalence	H5§	0/901 BaPFW, AgW, AnFW, OW by MNA confirmed by IFA	n.i.	n.i.	n.i.
[46]	Thailand, 2005-2008	PF / HP H5N1	Longitudinal	H5§	2.4% (6/242 PW, PFW, PCu) by MNA, confirmed by WBA. [6/242 (6/6), no s.c.]	n.i.	No	n.i.
[47]	Peru, 2006	Large PIn / AIV	Seroprevalence (c=17)	H4-H12	0/132 InPFW for H4-H12 by MNA.	Yes	No	n.i.
[48]	Nigeria, 2006	PF, PM, Lab / HP H5N1	Longitudinal	H5	0/295 PFW, PMW, PDeW, Vet and 0/25 LW by HIA and MNA.	Yes	n.i.	n.i.
[49]	Turkey, 2006	PF / HP H5N1	Seroprevalence	H5	60% (3/5 H5N1 cases), 3.6% (1/28 H5N1 cases-FaM), 0/95 PCu, 0/75 subjects exposed to diseased poultry and 0/81 non-exposed people from the same area, 0/97 HCW by MNA (confirmatory of ELISA and HIA). [97/97 (0/97) HCW, no s.c.]	n.i.	n.i.	n.i.
[50]	Germany, 2006	Wetland WLH / HP H5N1	Seroprevalence	H5	0/78 (WLE-firemen, WLE-GoW, WLE-Vet) by MNA, confirmatory test of PNA.	Yes	Yes	n.i.
[51]	Japan, 2006	H5-AI-free PF, geographic area where an H5N2 outbreak previously occurred / H5N2	Seroprevalence (c=100)	H5	**15.3%** (8/52 PW), 7% (8/114 general inhabitants in outbreak areas) by MNA for **H5N2**. MNA seropositive samples were also tested by HIA.	n.i.	n.i.	n.i.
[52]	England 2006	WpE(L/D)P / LP H7N3	Longitudinal	H7§	1.1% (1/91 WpE(L/D)PW) by MNA, HIA and WBA. [33/91 (0/33), no s.c.]	Yes	n.i.	n.i.
[53]	China, 2006, 2008	PF in HP H5N1 poultry outbreaks areas; PMePrP and SH / H9N2 and H7 AI	Seroprevalence (c=407)	H7, H9	1.5% (12/783) FW and PW for H9; 0/783 FW and PFW for H7; 0/277 PW for H7 and H9 from different regions by HIA	Yes	n.i.	n.i.
[54]	USA, 2007-2008	WLH / HP H5N1	Cross-sectional	H5	0/712 WBHu, RSBHu, RSBHu-FM, USpHu, WLB by MNA, confirmed by HIA and WBA	Yes	n.i.	n.i.
[55]	USA, 2007-2008	BaPF, large PIn, PMePrP / AIV	Cross-sectional study (c=82)	H4-H11	20.0% (4/20 **BaTG**), 0/35 InTFW, 8.3% (3/36 TMePr) for H4, 30.0% (6/20 **BaTG**), 5.7% (2/35 InTFW), 0/36 TMePr for H5,	Yes	Yes	n.i.

					20% (4/20 **BaTG**), 0/35 InTFW, 0/36 TMePr for H6,<br>0/20 BaTG; 0/35 InTFW, 0/36 TMePr for H7<br>10.0%% (2/20 **BaTG**), 0/35 InTFW, 2.8% (1/36 TMePr) for H8,<br>20.0% (4/20 **BaTG**), 0/35 InTFW, 0/36 TMePr for H9,<br>25.0% (5/20 **BaTG**), 0/35 InTFW, 2.8% (1/36 TMePr) for H10,<br>5.0% (1/20 BaTG), 2.9% (1/35 InTFW), 2.8% (1/36 TMePr) for H11 by MNA.<br>BaTG GMT significantly higher for **H4**, **H5**, **H6**, **H8**, **H9**, **H10**			
[56]	Indonesia, 2007	Small and large CoPF / HP H5N1	Seroprevalence	H5	0/495 CoPFW by HIA and NT	Yes	Yes	n.i.
[57]	USA, 2008, 2009, 2010	WLH / H5N2, H7N2, H7N3, H9N2	Cross-sectional	H5, H7, H7§, H9	0.24% (1/401 BHa) for H5, 0/401 BHa for H7 and H9 by MNA	n.i.	Yes	n.i.
[58]	Nigeria, 2008-2010	PIn, open BaPF and LBM / AIV	Seroprevalence (c=54)	H4-H8, H9§, H10-H12	0.3% (1/315 PInW, BaPFW, PMW) for LP H5N2,<br>1.3% (4/315 PInW, BaPFW, PMW) for H9,<br>0.9% (3/315 PInW, BaPFW, PMW) for H11 by MNA;<br>0/315 for H4, H6-H8, H10, H12.	Yes	Yes	n.i.
[59]	China, 2008-2010	WM / H9N2	Seroprevalence (c=1730)	H9	**12.5%** (103/826 WMW) for **H9** by HIA, in strong concordance with MNA.	n.i.	n.i.	n.i.
[60]	China, 2008-2012	SF ponds / HP H5N1	Seroprevalence (c=104)	H5	0.6% (10/1606 SFW, SFW-FM, Vet, OW) by NT, confirmatory test of HIA.	n.i.	n.i.	n.i.
[61]	Italy, 2008-2010	InPF / LP H5N2, H5N7, H7N3, LP and HP H7N1	Seroprevalence (c=379)	H5, H7	0/188 InPFW for H5; **3.2%** (6/188 InPFW) for **H7** by MNA, confirmatory test of HIA.	n.i.	n.i.	n.i.
[62]	Bangladesh, 2009	PF, wholesale and retail LBM / HP H5N1	Seroprevalence	H5§	0/422 PFW, LBMW, DeW by HIA and WBA, confirmatory tests of MNA.	Yes	n.i.	Yes
[63]	Mongolia, 2009	AgA / AIV including HP H5N1	Seroprevalence (c=81)	H4-H8, H9§, H10	0.3% (1/358 AnFW) for H5, 0.3% (1/358 AnFW) for H6, 0.8% (3/358 AnFW) for H9, 0/358 for H4, H7, H8, H10 by MNA	n.i.	Yes	n.i.
[64]	Iran, 2009	PF, SH / H9N2	Seroprevalence (c=60)	H9	**90%** (54/60 PFW), **78.3%** (47/60 SHW), **71.6%** (43/60 Vet), 46.6% (28/60 ill-patients), for **H9** by HIA	n.i.	n.i.	n.i.
[65]	Bangladesh, 2009-2010	LBM / HP H5N1	Seroprevalence (c=101)	H5§, H5	2% (9/404 LBMW) for H5 by WBA,<br>confirmatory test of MNA.<br>[278/387 (5/278), 6 s.c.]	Yes	n.i.	n.i.

[66]	Romania, 2009-2010	Large-scale CoSF, BaPF / AIV including HP H5N1	Seroprevalence (c=51)	H4–H8, H9§, H10-H11	**9.1%** (33/363 AgW including CoSFW, BaPFW) to **H9§,** 0/363 for H4, HP and LP H5, H6-H8, H10, H11 by MNA	n.i.	Yes	n.i.
[67]	China, 2009-2010	PF, SH / HP H5N1, H9N2	Seroprevalence.	H5, H9	0/305 PFW and SHW for H5; 3.7% (10/269 PFW), 11.1% (4/36 SHW) for H9 by MNA.	Yes	No	n.i.
[68]	China, 2009-2011	LPM, large-scale PF and BaPF, SH, WBH / H9N2	Seroprevalence	H9§, (G9) H9 (G1)	3.88% (156/4021 LPMW), 2.97% (115/3872 large-scale PFW), 3.15% (130/4121 BaPFW), 2.12% (26/1228 SHW), 1.66% (11/664 WBHW), 1.52% (15/990, OW) for H9§ (G9) and 1.09% (38/3480 LPMW), 1.65% (57/3463 large-scale PFW), 1.3% (52/3985 BaPFW), 0.52% (5/970 SHW), 0.88% (5/565 WBHW), 0.19% (2/990, OW) for H9 (G1) by MNA	n.i.	n.i.	n.i.
[69]	China, 2009-2012	Lakes with wild birds near SF, sometimes including poultry / H9N2	Seroprevalence (c=83)	H9	**1.2%** (24/2006 SFW, SF owners and Vet, SFW-FM) for **H9** by MNA, confirmatory test of HIA	Yes	n.i.	n.i.
[70]	Egypt, 2010-2013	BaPF, LBM / H7 AI	Seroprevalence (c=250)	H7	0/565 BaPG and LBMW for H7N7 by MNA confirmed by WBA [1st follow-up: (13/682) PW, 2nd follow-up: (14/649) PW); 27 s.c.]	n.i.	no	n.i.
[71]	China, 2010	BaPF near waterbird habitats / HP H5N1	Seroprevalence	H5§, (2010) H5§ (2005)	0/306 BaPFW for 2010 H5N1 and 2.61% (8/306 BaPFW) for 2005 H5N1 by HIA	n.i.	n.i.	n.i.
[72]	Lebanon, 2010	BaPF, CoPF / AIV	Seroprevalence (c=50)	H4-H6, H7§, H8-H16	2.3% (3/128 **BaPG**) for H4, 1.6% (2/128 **BaPG**) for H11, 0/128 BaPG for H5-H10, H12 and 0/72 CoPG for H4-H16, by MNA confirmed by HIA	Yes	Yes	n.i.
[73]	India, 2010	CoPF, wet LBM in urban, semiurban or rural areas / H9N2	Seroprevalence (c=249)	H9	6.2% (21/338 PW) by either HIA or MNA, 4.7% (16/338 PW) and 3.8% (13/338 PW) by HIA and MNA respectively, 8/21 by both HIA and MNA.	n.i.	No	n.i.
[74]	Pakistan, 2010-2011	CoPF / HP H5N1 and H7N3, H9N2	Cross-sectional	H5, H7, H9	0/354 CoPFW for H5, 21.2% (75/354 CoPFW) for H7, 47.8% (169/354 CoPFW) for H9 by HIA.	n.i.	No	n.i.
[75]	China, 2010-2012	Large-Scale CoPF, urban and rural LBM; BaPF, SH, PMePrP / HP H5N1	Cross-sectional	H5§, (2010) H5§ (2005)	5.4% (13/241 LBMW), 4.7% (25/537 CoPFW), 0/36 PMePr and SHW, 4.8% (17/355 BaPFW-FM) for 2010 or 2005 H5N1 by MNA confirmatory test of HIA.	n.i.	n.i.	n.i.
[76]	South Africa, 2011-2012	OF / HP H5N2, LP H7N1	Seroprevalence (c=38)*	H5, H7	1% and 2% (2/207 and 4/207 DeW, FW, Vet, OW for H5 and H7, respectively; in detail 1/121 FW and 1/9 OW for H5, 4/56 SHW for H7) (1st survey). 1.5% and 13% (1/66 and 8/66 DeW, FW, LW, Vet for H5 and H7 respectively; in detail 1/2 researcher for H5,	Yes	Yes	n.i.

					6/41 SHW, 1/4 LW, 1/8 Vet staff (2nd survey).<br>2.7% and 10.8% (1/37 and 4/37 Vet) for H5 and H7 respectively (3rd survey).<br>All samples tested by HIA confirmed by MNA			
[77]	China, 2011	CoDF, BaDF, slaughtering sites / H5, H7, H9 AI	Seroprevalence	H5, H7, H9	0/1741 DFW for H5 and H7, 0.7% (12/1741 DFW) for H9 by HIA	Yes	n.i.	n.i.
[78]	Pakistan, 2011	PIn, LBM / H9N2, H7 AI	Seroprevalence (c=25)	H9, H7	85.7% (6/7 **vaccinators**), 46.6% (165/354 poultry attendants), 38.9% (7/18 LW), 38.1% (24/63 butcher/retailers), 30.4% (7/23 Vet) for H9 and 44.4% (8/18 LW), 42.9% (3/7 **vaccinators**), 26.1% (6/23 Vet), 31.4% (111/354 poultry attendants), 11.1% (7/63 butcher/retailers) for H7 by HIA.	n.i.	No	n.i.
[79]	Viet Nam, 2011	LPM / HP H5N1	Seroprevalence	H5§ (C 1),<br>H5§ (C 2.3.4),<br>H5§ (C 2.3.2.1)	0/607 LPMW (sellers and slaughters) for H5 (C1);<br>4% (24/607 LPMW) for H5 (C 2.3.4),<br>0.3% (2/607 LPMW) for H5 (C 2.3.2.1) and 1.8% (11/607 LPMW) for both, by HIA confirmed by MNA	n.i.	n.i.	n.i.
[80]	China, 2011-2012	WpE(L/D)P / HP H5N1	Seroprevalence	H5	0/406 Vet by both HIA and NT	n.i.	No	n.i.
[81]	China, 2011-2013	PF, SH, wholesale and retail PM / H9N2	Seroprevalence (c=600)	H9	**8.5%** (51/600 PFW, SHW, PMW) for **H9** by HIA confirmed by MNA	n.i.	No	n.i.
[82]	China, 2011-2012	CoPF / H9N2	Seroprevalence (c=100)	H9	2.3% (9/382 CoPFW) by either HIA (9/382) or MNA (7/382)	n.i.	No	n.i.
[83]	Taiwan, 2012	Live poultry stalls, PF / LP and HP H5N2, H6N1, LP H7N3, H7N9	Seroprevalence (c=577)	H5, H6§, H7, H7§	2.99% (10/335 LPV) and 1.79% (6/335 PFW) for H5N2;<br>0.6% (2/335 LPV) and 1.19% (4/335 PFW) for H7N3;<br>0.3% (1/335 PFW) and 0/335 LPV for H6N1;<br>0/335 PFW and LPV for H7N9§ by HIA.	Yes	Yes**	n.i.
[84]	Iran, 2012-2013	PF, SH, university poultry hospital / H9N2	Seroprevalence (c=100)	1998 H9 (G1, sub-lineage A).<br>2008 H9 (G1, sub-lineage B).	Sub-lineage B: 17% and 12% (17/100 and 12/100 PFW, SHW, Vet) by MNA and HIA respectively;<br>in detail 3/20 PFW, 12/70 SHW, 2/10 Vet by MNA and 1/20 PFW, 10/70 SHW, 1/10 Vet by HIA.<br>Sub-lineage A: 2.0% (2/100 PFW, SHW, Vet) by both MNA and HIA; in detail 0/20 PFW, 1/70 SHW, 1/10 Vet.	n.i.	No	n.i.
[85]	China, 2012, 2013, 2014	Wholesale LPM, meat markets or retail LPM, BaPF, PF / HP H5N1, HP and LP H7N7, H7N9, H9N2	Seroprevalence (c=96)***	13 different HA (HA1-part) of 5 H5§, 3 H7§ and 2 H9§, 3 9	Differences in Ab profiles between controls and 371 PW were consistent with exposure and a **higher prevalence** of Ab to **H5** and **H9** was detected in PW by protein microarray. H7 Ab were less commonly detected, only in PW from 2 LPM by protein microarray.	n.i.	Yes	n.i.

[86]	Cambodia, 2013	LBM / HP H5N1, H9N2, H7N9	Longitudinal	H5§ (C 1.1.2), H9§§, H7§	During 4 surveys (within 2013) an overall prevalence of 4.5% (5/111), 1.8% (2/111), 0/111 LBMW for H5, H9 and H7 respectively was found by both HIA and MNA. [4 s.c. for H5, 1 s.c. for H9]	n.i.	n.i.	n.i.
[87]	China, 2013	PF, LPM, pet birds exposure areas / H10N8	Seroprevalence	H10§	0/400 Vet for H10 by MNA, confirmatory test of HIA	n.i.	n.i.	n.i.
[88]	China, 2013	SF, PF, retail PM, SH, zoo, vet structures / H10N8	Seroprevalence (c=107)	H10§	0.4% (3/710 animal workers: 1 Vet, 1 PW and 1 retail PMW for H10 by MNA, confirmatory test of HIA	n.i.	No	n.i.
[89]	China, 2013-2014	CoPF, small-scale CoPF, LPM and wholesale market / H7N9	Seroprevalence	H7§	0.1% (1 LPM butcher and 1 wholesale market seller/1866 CoPFW, small-scale CoPFW, LPMW, wholesale market seller) by MNA, confirmatory test of HIA	Yes	Yes	n.i.
[90]	China, 2013-2014	SF, PF, LPM (including pigs), pet veterinary structures / HP H5N1, H9N2, H7N9	Cross-sectional (c=264)	H5, H7, H9	0.6% (1/171 SFW), 2% (3/150 PFW), 4.8% (5/105 LPMW), 1.17% (2/120 Vet) for H5; 1.2% (2/171 SFW), **4.7%** (7/150 PFW), **17.1%** (18/105 LPMW), 1.17% (2/120 Vet) for **H7**; 3,5% (6/171 SFW), **11.3%** (17/150 PFW), **22.9%** (24/105 LPMW), 2,5% (3/120 Vet) for **H9**, by HIA.	No	No	n.i.
[91]	China, 2013, 2014	LPM (wholesale markets or poultry retail markets), pig/cattle SH / HP H5N1, H7N9, H9N2,	Seroprevalence	H5§, H7§, H9§	For H5: 0/99 LPMW and SHW in 2013, 37.8% (17/45 **LPMW)** and 3.7% (1/27 SHW) in 2014 by HIA. For H7: 10% (3/30 LPMW) and 13% (9/69 SHW) in 2013, 2.2% (1/45 LPMW) and 0/27 SHW in 2014 by HIA. For H9: 10% (3/30 LPMW) and 8.7% (6/69 SHW) in 2013; 55.6% (25/45 **LPMW**) and 14.8% (4/27 SHW) in 2014 by HIA. [22 followed up (10 LPMW, 12 SHW), 6 s.c. in LPMW and 1 s.c. in SHW for H5; 1 s.c. in LPMW and 1 s.c. SHW for H7; 5 s.c in LPMW and 2 s.c. in SHW for H9]	n.i.	Yes	n.i.
[92]	China, 2013-2015	Commercial or private PF and SF, SH / H7N9 and HP H5N1	Longitudinal (c= 1200, 1188, 1135 in 1st, 2nd, 3rd survey)	H5§ (C 2.3.4), H5§ (C 2.3.2.1), H7§	1st survey: 0/1258 PW, 0.1% (1/1332 SW) for H5 (C 2.3.4); 0/1258 PW, 0.2% (2/1332 SW) for H5 (C 2.3.2.1); 0/1258 PW, 0/1332 SW for H7N9; 2nd survey: 1.6% (17/1056 **PW**), 0.1% (1/1254 SW) for H5 (C 2.3.4); 0.2% (2/1056 PW), 0/1254 SW for H5 (C 2.3.2.1); 0/1258 PW, 0/1332 SW for H7N9; 3rd survey: 0.2% (2/1123 PW), 0.1% (1/998 SW) for H5 (C 2.3.4); 0.1% (1/1123 PW), 0.2% (2/998 SW) for H5 (C 2.3.2.1); 0.4% (4/1123 PW), 0/998 SW for H7N9,	n.i.	n.i.	n.i.

					by HIA. [16 PW s.c. for H7N9, 38 PW s.c. and 4 SW s.c. for H5N1 clade 2.3.4, 3 PW s.c. and 2 SW s.c. for H5N1 clade 2.3.2.1]			
[93]	China, 2013-2016	LPM including pigs / H7N9, H9N2, HP H5N1 and H5N6	Longitudinal (c=915)	H7, H5N6 (C 2.3.4.4), H5N1 (C 2.3.2.1c), H9	**0.93%** (9/964 LPMW) for **H7**, **1.87%** (18/964 LPMW) for **H9N2**, **1.87%** (18/964 LPMW) for **H5N1**, 0/964 LPMW) for H5N6 by MNA, confirmatory test of HIA. [2 s.c. for H7N9, 13 s.c. for H9N2, 7 s.c. for H5N1]	n.i.	Yes	n.i.
[94]	China, 2013-2016	Large scale CoPF, large scale SF, poultry and swine SH, private PF and SF, backyard P and S raising sites / H9N2	Longitudinal (c= 1200, 1188, 1135, 920 in 1st, 2nd, 3rd, 4th survey)	H9§§,	1st survey: 0.48% (6/1258 PW), 1.35% (18/1332 SW); 2nd survey: 1.61% (17/1056 PW), 1.12% (14/1254 SW); 3rd survey: 1.16% (13/1123 PW), 0.60% (6/998 SW); 4th survey: 2.77% (19/685 PW), 0.82% (5/610 SW), by HIA. [63 PW s.c., 33 SW s.c.]	n.i.	n.i.	n.i.
[95]	China, 2015-2016	Large, small and free-range PF, SH, wholesale- and retail-LPM / H9N2, H5 and H7 AIV	Longitudinal (c=232)	H5, H7, H9	0.7% (2/296 PW) for H7; 0.3% (1/296 PW) for H5; 8.1% (24/296) PW of which: 5.1% (4/77) PFW, 8.3% (14/168) **wholesale LPMW**, 12.8% (6/47) **retail LPMW** for H9N2, by MNA confirmatory test of HIA	n.i.	Yes	n.i.
[96]	Pakistan, 2016-2017	WpE(L/D)P, SH, AI diagnostic Lab / H9N2	Seroprevalence	H9	50.3% (167/332) PW of which: 100% (19/19 **LW**), 38.5% (15/39 field Vet); 83.3% (15/18 vaccinators), 52.4% (11/21 butchers), 45.5% (107/235 PFW) for H9 by HIA.	Yes	n.i.	n.i.
[97]	Korea, 2016-2017	PF / HP H5N6	Seroprevalence	H5	0/523 PFW by MNA [523/523, 0 s.c.]	n.i.	Yes	n.i.

#, in red bold font statistically higher occupational risk in workers; ##, in black bold font statistically significant difference in AIV seroprevalence between exposed and unexposed controls; PPE, personal protective equipment; Vax, seasonal influenza vaccination; antiV, antiviral prophylaxis and/or therapy; s.c., seroconversion(s); n.i., no information; c, unexposed controls; G1, G1 lineage; G9, G9 lineage; C, clade; *, as control group, veterinarians involved in animal disease control, including AI outbreaks in poultry; **, H5N1 and/or seasonal influenza vaccination; ***, as control group, healthy blood donors who were not specifically asked about poultry exposures.§, virus of avian origin but isolated from a human; §§, virus of avian origin but isolated from the environment.
