# Supplementary material for: Occupational Risk from Avian Influenza Viruses at Different Ecological Interfaces Between 1997 and 2019
Source: Microorganisms. 2025 Jun 14;13(6):1391. doi: 10.3390/microorganisms13061391 (PMC12195780; doi:10.3390/microorganisms13061391)
Supplement: Supplementary file 1 [file microorganisms-13-01391-s001.zip › Table S3.pdf]

Table S3. Mixed—virological and serological—results obtained from risk groups occupationally exposed to AIV. See Table footer, Table A1, Table S6, Table S7, and Table S8 for acronyms and/or further details.

Ref.	Country, Year(s)	Workplace / potential AIV exposure	Study Design	AIV detection and further characterization (pos./tested)	HA sutype(s) of AIV Ag	Seroprevalence % (pos./tested)#, ## [Follow-up (pos./tested), seroconversion]	PPE	Vax	antiV
[98]	USA, 2004, 2006	PF, PMePrP, WBH / AIV	Longitudinal (c=66)	IAV (22/74 set of n.sw. and gargle from 66 subjects followed for ILI) by rRT-PCR and VI-CC. 0/22 for AIV by RT-PCR and sequencing.	H4-H7, H9	0.5% (2/415) P-NE AgW for H4, 8.9% (34/381) P-E AgW and 7.5% (31/412) P-NE AgW for H5, 3.4% (13/378) P-E AgW and 1.5% (6/409) P-NE AgW for H6, 5.5% (21/380) P-E AgW and 3.2% (13/412) P-NE AgW for H7, 2.4% (9/379) P-E AgW and 1.5% (6/408) P-NE AgW for H9, by MNA. [740/740 AgW followed at 12 and 24 mo., 6 s.c. for H5, 2 s.c. for H9]	n.i.	Yes	n.i.
[99]	China, 2006	Large LBM / HP H5N1	Case report	HP H5N1 (1/1 t.sw. from hospitalized patient) by rRT-PCR and VI	H5 (1997) H5 (2004)	0.9% (1/110 LBMW) for both H5 strains by MNA, confirmatory test of HIA	n.i.	n.i.	n.i.
[100]	Nigeria, 2008-2011	Large-scale CoPF in AgA, open LBM / AIV including HP H5N1	Longitudinal (c=54).	AIV and H5N1 (0/124 followed for ILI AgW ph.sw., n.sw.) by rRT-PCR	H4-H8, H9, H12	0/316 P-E AgW for HP H5N1, 0.3% (1/316) P-E AgW for H5N2, 1.3% (4/316) P-E AgW for H9N2, 0.9% (3/316 P-E AgW) for H11N1; by MNA. See [58] for further details. [12-mo. follow-up: 2 s.c for HP H5N1; 24-mo. follow-up: 3 s.c. for HP H5N1].	n.i.	n.i.	n.i.
[101]	USA, 2009, 2010	Bird banding sites in WBH / AIV	Cross-sectional (c=78)	AIV and H5N1 (0/3 followed for ILI BBa c.sw., n.sw., t.sw.) by rRT-PCR, and subsequent reactions to determine influenza HA subtype.	H4-H11	0.6% (1/157 BBa) for H7N3, 0.6% (1/157 BBa) for H9N2, 0.6% (1/157 BBa) for H11N3 and 0/157 BBa for H4-H6, H8, H10, by MNA	Yes	Yes	n.i.
[102]	Mongolia, 2009-2011	AgA / AIV including HP H5N1	Longitudinal (c=81)	IAV (36/100 set of n.sw. and p.sw. from ILI cases during follow-up) by rRT-PCR,	H4-H8, H9§, H10-H12	See [63] for enrollment results. [1st (12-mo.) follow-up: 363/439 (1/363 for H6N1, 4/363 for H9N2,	n.i.	Yes	n.i.

				5/36 subtyped by rRT-PCR were AIV negative.		0/363 for H10N4) 2nd (24-mo.) follow-up: 351/439 (2/351 for H6N1, 4/351 for H9N2, 1/350 for H10N4. No s.c.]			
[103]	Australia, 2010	Large-scale CoCF, SH / H10N7 confirmed outbreak	Case report Seroprevalence (c=200)	H10 AIV (1/6 SHW c.sw. and 1/7 SHW n.sw. and t.sw) by partial sequencing analysis following IAV screening by PCR	H10	[7/7 (0/7), no s.c.] by HIA or VNA	No	n.i.	n.i.
[104]	Indonesia, 2012-2016	LPM / HP H5N1	Seroprevalence (c=121)	H5N1 (0/101 LPMW including sellers, butchers op.sw in Feb. 2014 by rRT-PCR.	Eurasian H5 (C 2.3.2.1)	Apr. 2012: 48% (30/63 LPMW), Feb. 2014: 84% (85/101), Feb. 2015: 59% (59/100), Mar. 2016: 26% (37/142) for H5 (C 2.3.2.1) by HIA. [1st follow-up: 25/101 (19/25), 11 s.c. 2nd follow-up: 36/100 (22/36), 1 s.c. 3rd follow-up: 58/142 (15/58), 3 s.c.]	n.i.	n.i.	n.i.
[105]	China, 2013	Wholesale LPM, SH / H7N9	Seroprevalence (c=1129)	H7N9 (0/396 PW n.sw.) by RT-PCR	H7§	**6.3%** (25/396 PW) for **H7N9**§ by HIA	n.i.	Yes	n.i.
[106]	China, 2013	LBM with L/D-birds, swine, dogs and cats / H7N9	Case-report	H7N9 (9 LBMW of which 6 slaughtered poultry, 2 transported live poultry, 1 traded and raised pigeons/139 persons with laboratory-confirmed H7N9 infection). 138/139 H7N9 cases confirmed by rRT-PCR or VI-EE	H7§	1/139 (1%) H7N9 cases was confirmed by HIA serologic testing. 5/138 virologically confirmed cases were also Ab positive when tested for H7N9§ virus by HIA.	n.i.	n.i.	n.i.
[107]	China, 2013-2015	LPM, PF, BaPF, SH/PMePrP, WBH, WpE(L/D)P / H7N9	Cross-sectional	H7N9 (81 PFW/ 170 hospitalized cases) by rRT-PCR and VI-EE	H7	3.7 % (34/912) PW, including 7.7% (22/284) **BaPFW**, 3.7% (11/300) **PFW**, 0.4% (1/227) LPMW, 0/28 SHW/PMePr, 0/26 WBH exposed, 0/47 OW, for H7N9 by HIA	n.i.	n.i.	n.i.
[108]	China, 2013-2014	Retail and wholesale LPM, PF /H7N9	Seroprevalence	H7N9 (1 /321 LPMW t.sw.) by RT-PCR, 0/6 ILI LPMW retested	H7§	1.6% (5/316 LPMW) for H7N9 by HIA	Yes	Yes	n.i.

[109]	China, 2013	Retail and wholesale LPM / H7N9, H5N1	Seroprevalence (c=417 in March, 408 in Sept)	H7N9 (0/501 LPMW n.sw.) by r-RTPCR	H5§, H7§	May 2013: 7.2% and 0.8% (36/501 and 4/501 LPMW) for H7N9 and H5N1 respectively, by HIA.<br>Dec. 2013: 14.9% and 0.8% (56/375 and 3/375 LPMW) for H7N9 and H5N1 respectively by HIA.<br>[96/501, 52 s.c. for H7N9]	n.i.	Yes	n.i.
[110]	China, 2014	LBM / H7N9	Longitudinal	H7N9 (1/1 LBMW t.sw. and s.sw, 2/2 LBMW-FaM t.sw. and s.sw) by rRT-PCR and genome analysis.<br>0/25 LBMW-close contacts by rRT-PCR	H7§	0/25 LBMW close contacts for H7N9 by both MNA and HIA	n.i.	n.i.	Yes
[111]	China, 2014	Small-scale PF / AIV including H7N9, H9N2	Seroprevalence	H7N9 (1/1 hospitalized PFW t.sw.) by rRT-PCR performed to detect H1-H16 and N1-N9 AIV,<br>by VI-EE, genome sequencing and genetic analysis	H7§	0/56 acute-phase and 0/47 convalescent-phase PFW close contacts for H7N9 by HIA	No	n.i.	n.i.
[112]	Russia, 2014-2015	PF / H7N9, HP H5N1 and H5N8 AIV	Seroprevalence	0/44 influenza viruses obtained by VI-CC from autopsy (n. 19) and clinical materials (n. 25) of severe flu-like cases in the 2014-2015<br>epidemic season	H7§<br>H5 (C 2.2.3)<br>H5 (C. 2.2.4.4)	0/1939 PFW for H7N9, H5N1, H5N8 by HIA	n.i.	n.i.	n.i.
[113]	Cameroon, 2016-2017	PF, LBM with (L/D)P / HP H5N1	Longitudinal	H5 and H7 AIV (0/663 PFW, LBMW np.sw and op.sw) by rRT-PCR	H5§§, (C 2.3.2.1)c	2.3% (3/131 PFW and LBMW) for HP H5N1 by HIA,<br>confirmed by MNA.<br>[131/131, 2 s.c.]	n.i.	n.i.	n.i.

#, in black bold font statistically significant difference in AIV seroprevalence between exposed and unexposed controls; ##, in red bold font statistically higher occupational risk in workers; PPE, personal protective equipment; Vax, seasonal influenza vaccination; antiV, antiviral prophylaxis and/or therapy; s.c., seroconversion(s); n.i., no information; c, unexposed controls; G1, G1 lineage; G9, G9 lineage; C, clade; §, virus of avian origin but isolated from a human; §§, virus of avian origin but isolated from the environment.
