# Supplementary material for: Occupational Risk from Avian Influenza Viruses at Different Ecological Interfaces Between 1997 and 2019
Source: Microorganisms. 2025 Jun 14;13(6):1391. doi: 10.3390/microorganisms13061391 (PMC12195780; doi:10.3390/microorganisms13061391)
Supplement: Supplementary file 1 [file microorganisms-13-01391-s001.zip › Table S4.pdf]

Table S4. Additional information related to Table S1 (Virological results obtained from risk groups occupationally exposed to AIV). See Table S7 and Table S8 for acronym details. **In red bold font,** statistically higher occupational risk in workers.

Ref.	Country, Year(s)	Workplace (animal species exposure)/ Potential AIV exposure	Concurrent studies in animals and/or environments	Virological methods used in humans	Personal protective equipment (PPE), vaccines, antiviral therapy	Main outcomes
[20]	Netherlands, 2003	CoCF (chicken) / HP H7N7	No	Swabs collected from both eyes, oropharynx and nasopharynx were initially screened (the first 25 cases) for influenza virus by cell culture and RT-PCR, positive samples were further typed and subtyped by HIA. RT-PCR for IAV was then used as screening method, followed by specific molecular methods for subtyping (H3, H7).	On 3 Mar. 2003, (A/H7N7 AI outbreak confirmation), workers who screen and cull poultry were advised to wear protective eyeglasses and mouth and nose masks. On 7 Mar. 2003, (first case of A/H7-conjunctivitis confirmation in humans) mandatory vaccination with inactivated influenza virus vaccine was recommended to all PW who handle, screen, or cull potentially infected chickens. When 19 people were diagnosed with the infection, all PW received mandatory influenza virus vaccination and prophylactic treatment with oseltamivir. 56% of A/H7 infections reported arose before the vaccination and treatment programmed.	After the first case of A/H7-conjunctivitis was confirmed in a vet who visited several farms with HPAI-infected poultry flocks (vet who 15 days after exposure died of respiratory insufficiency) active human case finding was started from 10 Mar. 2003. An unexpectedly high number of transmissions of AIV subtype H7N7 was found in people directly involved in handling infected poultry, and evidence for person-to-person transmission was shown. Eye swabs were more frequently positive than were throat swabs. **Vets and cullers had the highest risk of A/H7 infection**. Characterization of the viruses found in the index case, the fatal case, and the three contact cases was done to exclude the possibility of spread of a reassorted influenza virus variant. All viruses characterized were completely of avian origin.

[21]	Vietnam, 2003, 2004	PF WpE(L/D)P (chickens, ducks) / HP H5N1	No	Patients' blood was tested by viral culture in monolayers of MDCK cells. Isolated viruses were identified by IFA and HIA. Nasal and throat swabs were tested by RT-PCR for H5 and N1 genes.	No information	Eight of ten patients died, one patient has recovered, and one is recovering. In all 10 patients the infection appears to have been acquired directly from infected poultry. None of the farmers' household members or relatives was sick.
[22]	Mexico, 2012	PF / HP H7N3	No	Conjunctival swabs from both patients were tested by rRT-PCR by using an H7 gene specific assay. Viral isolation from patient 1 was performed using embryonated chicken eggs. Viral antigenic characterization was performed by a turkey RBC-based and HIA using a panel of ferret antisera. Nucleotide sequences of 8 IAV gene segments were generated by NGS.	No information	The isolate obtained from patient 1 had a multibasic cleavage site indicative of an HPAIV that might have been derived from recombination with host rRNA.
[23]	China (Zhejiang Province), 2013	Wholesale WM / H7N9	No	Pharyngeal and sputum swabs were collected from 1 hospitalized worker. Pharyngeal swabs form the patient's 60 co-workers tested by rRT-PCR for H7N9.	Workers examined wore protective clothing, ordinary disposable masks and latex gloves. Neither goggles nor face shields were used.	The hospitalized worker developed mild upper respiratory symptoms 6 days after the contact with H7N9 infected poultry. Virus persisted longer in sputum than in pharyngeal swab samples collected.
[24]	Italy, 2013	InPF (chicken)/ HP H7N7	No	Conjunctival swabs were tested by two rRT-PCR to confirm influenza virus subtype H7N7. Virus isolation was performed on MDCK cells. RT-PCR products were sequenced and full genome sequencing was also performed on patient 3. Antigenic characterization of H7N7 was performed by turkey RBC-based HIA and H7 reference antisera.	Patients 1 and 2 had not used PPE until when H7N7 infection in poultry was diagnosed. Thereafter, they were involved in culling and wore PPE, including face masks with eye protection. Patient 3 participated in depopulation procedures wearing PPE.	This study provides further evidence of H7 subtype–specific ocular tropism. Molecular findings suggest direct transmission of the virus from chickens to humans. All 3 patients were isolated at home; without specific antiviral treatment, symptoms resolved in a few days.
[25]	China (Beijing), 2014	WpE(L/D)P / H7N9	No	Oropharynx swab was tested by rRT-PCR for the H7N9 subtype. Virus isolation was performed in embryonated chicken eggs. To study the gene evolution of the H7N9 virus, the whole genome was amplified and sequenced.	No information	The novel H7N9 virus probably emerged and further reassorted with other H9 or H7 viruses in poultry before transmitting to humans. These findings indicate that humans became infected by direct contact

						with poultry, in the absence of human-to-human transmissibility.
[26]	China (Jilin Province) 2014	Small-scale PF (turkeys, guinea fowls, chickens, goose commingled in an egg production warehouse / H7N9	Yes<br>i) Dates of illnesses and deaths among bird flock on poultry farm (including around 1100 newly-introduced birds),<br>ii) the case patient's symptom onset, and<br>iii) confirmed testing results were recorded for epidemiological investigation.<br>Samples from the poultry farm, tested by rRT-PCR, showed that: 19/84 poultry and environmental specimens were positive for both H7N9 and H9N2 AIV, 1/84 for H7N9 only, and 3/84 for H9N2 only.	On February 19, 2014, throat swab samples were taken from the hospitalized case patient, and the same day tested by rRT-PCR: positive results for H7N9 AIV were obtained and confirmed the following day.<br>The virus from the case-patient's specimens, isolated in egg culture, was designated A/Jilin/10117/2014(H7N9), and full sequence are available from GISAID.	The case-patient cared for the poultry farm birds daily and did not use personal protective equipment.<br>His previous vaccination history was unknown.<br>Treatment with oseltamivir was administrated to the case patient.	After treatment with oseltamivir, the case patient recovered, and was discharged from the hospital on March 7, 2014.<br>Notably, the case of H7N9 AIV human infection, occurring in Jilin Province in northeastern China, was associated with a poultry farm rather than a live bird market, pointing out a new focus for improved surveillance and biosecurity on poultry farms involved in the H7N9 emergency.

			None of the birds received AIV vaccines on the case-patient's farm, and previous vaccination histories were unknown			
[27]	Egypt, 2015	CoPF, BaPF / H5 viruses	Yes. 75 samples from 10 commercial chicken farms (n. 50) and 5 duck flocks (n. 25), showing high mortality rates and suspected to be infected with AIVs, were collected. 25 wild egrets were also sampled. AIVs were only detected in chicken samples (18%) and molecularly confirmed as subtype H5.	Oropharyngeal and nasal swabs collected from asymptomatic PFW were tested by rRT-PCR for the identification of H5 gene (specific for AIV subtype H5) and H1 gene (specific for influenza A (H1) pdm09).	20/65 (31%) of the examined participants received influenza vaccine.	This study showed the HPAI H5N1 virus endemic circulation in poultry (especially in broilers than in layers) despite large-scale vaccination campaigns. All the avian isolated AI H5 viruses were clustered into clade (2.2.1.2) and shared a high similarity rate at nucleotides and amino acid levels. HPAI H5 virus infection in poultry farms was significantly associated ($p \leq 0.05$) to chicken breed, workers' movement among flocks, lack of utensils' disinfection, new birds' introduction to the farm. Co-circulation of AI H5 viruses in birds and pdm09 viruses in humans raises alarm for the emergence of reassortant viruses posing risks for avian and human health. Lack of positivity to H5 viruses in humans could be explained by a partial protection of influenza vaccine.
[28]	Pakistan, 2015-2016	BaPF, CoPF / AIV	No	Nasal swabs samples were individually inoculated in embryonated chicken eggs and amnio-allantoic fluid harvested after 48 h incubation was first tested by HA assay and	No information	The positive PW had not major signs of ILI. The sequencing of H and N genes of A/Pakistan/486/2015 (H9N2) suggested

				positive samples were screened by HIA for H5, H7, H9 AIV. Virus isolate was confirmed as H9N2 by RT-PCR, and HA and NA sequencing.		direct cross-species transmission from poultry to humans.
[29]	Cameroon, 2016-2017	CoPF, BaPF, LBM / HP H5N1, H5N8	Yes. Cloacal and tracheal swabs from birds (including broilers, laying hens, backyard chickens, turkeys, guinea fowls, ducks, layer breeders, geese, Indian peafowl, pigeon) were collected and examined for the AIV detection by RT-PCR. Two HPAIV (H5N1 and H5N8) strains were detected.	All human blood samples were tested by RT-PCR for H5N1, and H5N8 AIV detection.	No information	Despite the occurrence of H5N1 and H5N1/H5N8 HPAI outbreaks leading to high mortalities of poultry in commercial, backyard, and exotic farms (in 2006 and 2016-2017), no human case was detected suggesting the circulation of not human-adapted AIV strains in the country.
[30]	Korea, 2017	PF / HP H5N6	No	Nasopharyngeal swabs were collected from depopulation workers with suspected infection (i.e. any respiratory symptoms as rhinorrhea, cough, or shortness of breath with or without fever within 10 days following their last exposure to affected poultry) and tested by rRT-PCR for IAV H5 detection.	Depopulation workers were provided with PPE: disposable coveralls, nitrile gloves, N95 half-mask, goggles, boots. Moreover, preventive antiviral prophylaxis (oseltamivir) was administered from the day of the first exposure to the 7 days after the last exposure.	Poultry-to-human transmission of HPAI H5N6 virus was not identified. Preventive control measures (PPE, chemoprophylaxis and culling method by asphyxiating poultry in containers) may have contributed to decrease the risk of bird-to-human virus transmission.

					Overall, 2198 workers (47.4%) were already vaccinated against seasonal influenza before the AIV outbreak, whereas 2433 (52.5%) during the outbreak.	
