# Supplementary material for: Occupational Risk from Avian Influenza Viruses at Different Ecological Interfaces Between 1997 and 2019
Source: Microorganisms. 2025 Jun 14;13(6):1391. doi: 10.3390/microorganisms13061391 (PMC12195780; doi:10.3390/microorganisms13061391)
Supplement: Supplementary file 1 [file microorganisms-13-01391-s001.zip › Table S5.pdf]

Table S5. Additional information related to Table S2 (Serological results obtained from risk groups occupationally exposed to AIV). See Table S7 and Table S8 for acronym details. **In red bold font,** statistically higher occupational risk in workers.

Ref.	Country, Year(s)	Workplace (animal species exposure)/ Potential AIV exposure	Concurrent studies in animals and/or environments	Serological methods used in humans	Personal protective equipment (PPE), vaccines, antiviral therapy	Main outcomes
[31]	China, Hong Kong, 1997-1998	Retail and wholesale PM, hatchery, PF, other PIn (chickens and other domestic fowl)/ HP H5N1	No	Serum samples were tested by MNA for Ab to LPAI H5N3 virus A/Duck/Singapore/Q/F119-3/97 (anti-H5 titers >80 were considered positive).<br>Positive MNA results were confirmed by WB using a highly purified baculovirus-expressed HA protein from A/Hong Kong/156/97 (H5N1) virus.	GoPDeW often wore protective clothing, such as gowns, masks, and gloves, when working directly with poultry during the culling operation.	**GoPDeW had lower H5 seroprevalence (3%) than PInW (10%)** because of less prolonged exposures to poultry and most of them participated in depopulation of poultry on farms, not in **retail markets,** which **were more likely to have H5N1-infected poultry**.<br>For PInW, butchering poultry and exposure to poultry with >10% mortality was most highly associated with H5 positivity.
[32]	Taiwan, 1998-2000	Wholesale and retail PM (chickens, ducks)/ H3, H4, H6 AI	Yes.<br>About 1300 faecal samples were obtained from chickens (n. 600) and ducks (n.700), by sampling cages or trucks that were transporting birds to the poultry market A.<br>Samples was passaged in specific pathogen free (SPF) chicken embryonic eggs, and allantoic fluids testing positive by	Considering AIV H subtypes found in market A and avoiding possible cross reaction of current human NA subtypes (N1 and N2), human serum samples were tested for Ab against A/Duck/ Czechoslovakia/56 (H4N6) and A/Shearwater/Australia/1/72 (H6N5) by HIA and MNA, according to standard procedures.	No information.	Although through active market surveillance 28 avian influenza isolates were obtained, no seropositivity was found in PW and vets tested against 2 avian influenza virus strains (H4N6 and H6N5). These data indicate a very low possibility of direct transmission of new AIV strains to humans in Taiwan during the 1999-2000 season.

			HA assay were further subtyped by HIA and NIA. Allantoic fluids were tested by RT-PCR for sequencing. AIV isolation rates were 0% (0/580) in chickens and 4% (28/700) in ducks. 28 LPAIV, isolated from ducks were: 12 H3 (2 H3N1, 1 H3N2, 1 H3N6, 8 H3N8), 14 H4 (2 H4N1, 6 H4N6, 2 H4N4, 4 H4N6), and 2 H6N1 viruses.			
[33]	Italy, 1999-2003	InPF (turkeys and chickens)/ LP and HP H7N1and LP H7N3	No	983 serum samples were tested by a horse RBC-based HIA and MNA for Ab against H7N1 (A/Ty/It/2676/99 and A/Ty/It/3889/99) or H7N3 (A/Ty/It/214845/02) LPAIV.<br>Each serum sample was tested at least twice in separate MNA and sera that repeatedly had titers >20 were considered to be reactive in the MNA. A titer of 10 was considered to be a positive result in the HIA. WB was used as confirmatory assay for samples positive for either HIA or MNA test. A subset of sera was also tested by SRH assay (sera having reactive zones with diameters > 3.5 mm were considered to be positive).	No information	This study provides the first serological evidence of transmission of LPAIV to humans during an epizootic in domestic poultry. None of the 7 seropositive subjects reported a history of ILI.

[34]	Vietnam, 2001	Large LPM / HPAI or LPAI H5 or LPAI H9 virus	No	Serum samples were tested by MNA for Ab against four H5 and three H9 AIV: A/Goose/Vietnam/113/2001, HPAI H5N1; A/Duck/Vietnam/342/2001, LPAI H5N2; an HPAI clade 0 virus, A/Hong Kong/156/1997 (HK/156); an ancestral clade 1 virus, A/HongKong/213/2003 (HK/213). A/Duck/Vietnam /339/2001, LPAI H9N3; A/Hong Kong/1073/1999, LPAI H9N2 (G1 lineage, isolated from a child); a reassortant LPAI H9N7 virus possessing the HA of A/Chicken/Hong Kong/G9/1997 (Y280 lineage) and the NA from A/Equine/Prague/1/56 (H7N7) virus. MN titers >40 in duplicate test and confirmed by WB were considered positive. To minimize possible cross-reactivities with human IAV, sera H9 positive by MNA and WB were adsorbed with an H2N7 reassortant IAV possessing H and N of human and equine origin, and an H3N2 human IAV.	No information.	Seroprevalence detected to HPAI H5N1 virus Ab was low, and seropositive subjects most likely indicated relatively recent HPAI H5N1 infection in relation to sera collection in Oct. 2001. These data indicated the potential for HPAI H5N1 virus to cause asymptomatic infection or mild illness in adults. A similar low seroprevalence of Ab to LPAI H9N2 virus among PMW and controls was detected, suggesting that occupational exposure to poultry was not a major risk factor for LPAI H9N2 virus infection in the study period.
[35]	USA, Virginia, 2002	CoPF, Lab (turkeys and chickens) / LP H7N2	No	The LPAI A(H7N2) virus A/Tky/VA/4529/2002 (Tky/VA), isolated from a turkey from the index farm of the poultry outbreak, was used to test human sera by MNA (titers ≥1:80 were considered positive). Serum samples with duplicate titers of ≥80 were tested by WBA and an ELISA detecting IgM using H7 protein derived from whole purified Tky/VA virus. To rule out the possibility of Ab cross-reactivity between H7 and the	Use of PPE by the GoW involved in control outbreak activities varied by activity. Gloves were always worn by: 88% (44/50) of workers who touched dead birds; 84% (27/32) of workers who touched infected birds; 90% (22/29) of	The positive worker, showing ILI symptoms, had worked disposing infected birds using gloves always and dust mask most of the time, and never eye protection. Prior to outbreak, he had hunted waterfowl, but had limited exposure to domestic poultry. This study confirms that the North American lineage of LPAI A(H7N2) viruses can infect humans and cause respiratory

				A(H3N2) human subtype, virus adsorbed serum samples were tested.	workers who swabbed birds; 100% (14/14) of workers who collected environmental swabs; 77% (30/39) of workers who loaded culled birds on/off trucks. Mask was always worn by: 28% (14/50) of workers who touched dead birds; 31% (10/32) of workers who touched infected birds; 21% (6/29) of workers who swabbed birds; 50% (7/14) of workers who collected environmental swabs; 41% (16/39) of workers who loaded culled birds on/off trucks.	illness; however, the overall risk of infection is low.
[36]	USA, Delaware, Maryland, Virginia, 2003, 2005	Large PIn (chickens) / AIV	No	Serum samples were tested by MNA (a screening titers of 1:10 was utilized) for Ab against A/Duck/Cz/1/56(H4N6), A/Chucker /MN/14591-7/98(H5N2), A/Turkey/MA/6(H6N2), A/Turkey/VA/4529/02(H7N2), A/Chicken/DE/04(H7N2) A/Turkey/MN/38391-6/95(H9N2) LPAIV. Serum samples were also tested by a guinea pig RBC based HIA for Ab against human influenza A viruses	10/24 workers (42%) reported wearing dust masks during the course of work and 5 (29%) protective glasses. Given that nearly 30% of PW had no health insurance, it is likely that many had not received seasonal influenza vaccine, therefore	Lack of serologic evidence of AIV infection in PW could be more likely related to either the absence of AI in their occupational environments or the inefficient birds-to-humans transmission of viruses to which they were exposed. However, antigenic differences between AIV used in MNA and AIV circulating in the poultry cannot be ruled out.

				A/New Caledonia/ 20/99 (H1N1) and A/Panama2007/99 (H3N2).	elevated Ab reflect natural infection.	
[37]	South Korea, 2003-2004	CoPF (chickens, ducks) / HP H5N1	No	Overall, 3448 samples from 2512 persons were analyzed, among which paired samples were available from 936 (37%) subjects and single sample from 1576 (63%). Blood was collected from cullers on the day of culling completion and convalescent-phase blood samples were collected at least 4 weeks later.<br>As recommended by WHO, serum samples were tested by MNA for Ab against H5N1 virus (titer >80 to 2 independent assays were considered positive). MNA positive samples were confirmed by a horse RBC-based HIA or H5 specific WB (purified baculovirus-expressed influenza A/Vietnam/1203/2004 virus).	Persons who participated in the culling operations were equipped with WHO–recommended PPE. Previously non-vaccinated participants were vaccinated with a seasonal influenza vaccine and given oseltamivir as an additional prophylactic measure. However, the use of these preventive measures remained unclear.	Results obtained from poultry cullers showed that the frequency of poultry-to-human transmission of the influenza (H5N1) virus was low.
[38]	Canada, British Columbia, 2004	BaPF, CoPF / HP H7N3	No	All 167 sera were tested by a previously described MNA for Ab to the HPAI H7N3 human isolate (A/Canada/444/04).<br>A subset of 16 sera was also tested by WBA and by routine and modified HIA, using turkey RBC horse RBC, respectively.	Among 65 people who entered barns with infected birds, 55 (85%) had received influenza vaccine, 48 (74%) had received oseltamivir, and 55 (85%), 54 (83%) and 36 (55%) reported always wearing gloves, mask or goggles, respectively.<br>The use of PPE was less consistently reported by farmers for times of exposure	Management of the outbreak benefited from prior experience in the Netherlands (H7N7 HPAI outbreak in 2003). In fact, compliance with vaccination and prophylaxis was comparable, but reported compliance with PPE was generally higher among BC participants than among those surveyed in the Netherlands.<br>During the 2004 BC outbreak, 19/167 participants experienced ILI and 21/167 red or watery eyes, with no significant association between illness reports and exposure to infected birds.

					to infected birds than by workers for specific culling-related activities involving infected birds.	
[39]	USA, Iowa, 2004, 2005	Wetland WLH (wild waterfowl, game birds) / AIV	No	All serum samples were tested by MNA for Ab with influenza A subtypes H1 through H12 from avian sources. All hunter serum samples were tested by horse RBC-based HIA by using H11 AIV subtype A/H11N9/duck/Memphis/546/76 (titer >10 was considered positive).	In the 3 years before the study, influenza vaccination was administered to 37% of DHu and 35% of the Iowa Department of Natural Resources (DNR) workers. The 3 subjects with serological evidence of AIV infection had not been vaccinated against influenza within 3 years before the study; they did not wear PPE (glove, masks or eye protection).	The study provides evidence of past influenza A/H11 infection in persons who were routinely, heavily exposed to wild ducks and geese through recreational activities (duck hunting) or through their employment (bird banding). In particular, the DHu and the 2 DNR workers had 31, 27, and 30 years of duck-hunting experience, respectively. One of the positive DNR workers had several years of live wild duck–banding exposure in addition to 27 years of duck-hunting exposure.
[40]	USA, Iowa, 2004, 2006	WpE(L/D)P / AIV	No	Serum samples were tested by MNA for Ab to LPAIV subtypes from H4 to H12: A/Duck/Czechoslovakia/1/56 (H4N8), A/Chucker/Minnesota/14591-7/98 (H5N2), A/Turkey/Massachusetts/65 (H6N2), A/Turkey/Virginia/4529/02 (H7N2), A/Turkey/Ontario/68 (H8N5), A/Turkey/Minnesota/38391-6/95 (H9N2), A/Chicken/Germany/49 (H10N7), A/Duck/Memphis/546/76 (H11N9), A/Duck/Alberta/60/76 (H12N5).	Overall, 71.0% (76/107) of the subjects enrolled reported having received a flu vaccination within the last 3 years. Use of gloves and masks was inconsistent and infrequent.	**Vets who reported having examined AIV infected birds presented an increasing trend of being seropositive, compared with vets without this exposure and with control subjects.** Results emphasize that vets should be considered for priority access to vaccines and antiviral drugs in pandemic planning.

				Due to the low expected prevalence Because prevalence was expected to be low in the control group, sera were first screened at a dilution of 1:10, followed by 2-fold serial dilutions (from 1:10 to 1:1280) of all positive samples tested in duplicate. In vets, with expected higher reactivity, 2-fold serial dilutions from 1:10 to 1:1280 in duplicate were run on all serum samples. Horse RBC-based HIA was performed on all serum samples with positive MNA results and on a subset of serum samples with negative results for subtypes H5, H6, and H7. HI titers >1:10 were considered to be positive. Serum samples were also tested by HIA against 3 isolates of recently circulating human IAV (A/New Caledonia/20/99, H1N1; A/Panama/2007/99, H3N2; A/Nanchang/933/95, H3N2).		
[41]	China, Guangdong, 2004	Small scale outdoor PF (chickens, ducks) / HP H5N1	No	Serum samples were tested by HIA performed with cock RBC to detect Ab specific for two human influenza and three avian influenza viruses. Ag subtypes used were: H1N1 (A/New Caledonia/20/99); H3N2 (A/Panama/2007/99); H5N1 (A/goose /Guangdong/1/96) HPAIV; H7N1 (A/African starling/England-Q /983/79) LPAIV; and H9N2 (A/chicken/Shanghai/10/01). Ten HI positive samples were randomly tested by MNA. In both serologic tests, a titer of 1:20 or more was regarded as positive.	No information.	The results obtained implied the occurrence of H5N1 and H9N2 AI silent infections in Guangdong populations.
[42]	Thailand, 2004	InPF, BaPF (chickens, ducks,	No	Serum samples were tested by MNA for Ab against AIV subtype H5N1	No information.	Low titers detected (no study participant

		ornamental birds) / HP H5N1		(WHO definition of positive test result as a MN titer > 80 with a confirmatory ELISA or WBA).		had an anti-H5N1 antibody titer of >80, but 7 (2.2%) farmers had lower reactive antibody titers) might have resulted from: cross-reactivity with Ab of previous human influenza virus infection; mild or asymptomatic H5N1 infection; decay of Ab titers over time.
[43]	Vietnam, 2004-2005	Small-scale PF (chickens, ducks, geese, other birds) / HP H5N1	Yes. Sera from poultry were collected on site and analyzed by HIA using chicken RBC and A\Ck\Scot\59 strain as H5N1 Ag. Samples from dead poultry were tested by rRT-PCR for the H5 gene. A total of 65 positive sites (small-scale farms with a number of poultries ranging between 2 and 1600) were identified: 3/65 had dying chicken (confirmed to be infected with H5N1 by RT-PCR in 1/3). All other sites had ducks with positive HI tests. The number of samples tested per site varied between 1	Human serum samples were tested by: i) MNA using influenza A H5N1 strain A/ Vietnam/1194/2004 (Z5) (clade 1) and A/Vietnam/30850/05 (clade 2.3.4) with suppression of viral cytopathic effect as the end point; ii) MNA using influenza A H5N1 strains A/Vietnam /3212/2004 (clade 1), with suppression of virus antigen expression as assessed by ELISA as endpoint. Samples positive in this latter assay were analyzed for cross-reactivity to other IAV by adsorption tests with H1, H3 and H5 Ag. Single sera with a titer of 1:80 or more were considered positive in both MNA tests. Human sera which tested positive at any dilution in at least one of the two tests, were retested by HIA using horse RBC and by MNA with suppression of viral cytopathic effect as the end point, using a clinical isolate of influenza A (H5N1) virus (A/VN/CL26 /2004) representing the circulating virus at the time of the study.	Few PW reported the use of protective gear during their work. The 3 cullers showing HI titer of 1:80 had been involved in culling more than 1 year: 2 of them used only gloves and paper or cloth masks during culling, 1 culler also used a coverall and boots in addition to gloves and paper mask.	Although the study supported the low transmissibility of clade 1 H5N1 to humans, limited transmission to highly exposed persons cannot be fully excluded given the presence of low antibody Ab titers in some individuals.

			and 31 and the number of HI positive samples per site varied between 1 and 30.			
[44]	Japan 2005	Large commercial poultry farm (chickens) / LP H5N2	No	Paired serum samples (1st collected after virus detection in chickens, 2nd 4 weeks up to 2 months apart) were tested by NT for Ab against LPAIV A/chicken/Ibaraki/1/2005(H5N2). Titer > 1:40 were considered positive. Individuals showing 4-fold or greater rise in neutralizing Ab titer in paired sera were considered infected by the H5N2 virus (Ab titers measured at least twice for confirmation).	Seasonal influenza vaccinations were administered within the previous 12 months to the 28% of all subjects (n. 71).	The results suggest that this may have been the first avian influenza H5N2 infection of poultry to affect humans. However, statistical results indicate that: i) a history of seasonal influenza vaccination might be associated with H5N2-neutralizing Ab positivity; ii) aged individuals may have been exposed to more diverse influenza viruses.
[45]	Thailand, 2005	BaPF,AgA, rural villages (chickens or other poultry) / HP H5N1	No	Serum samples were tested by MNA for Ab against the HP A/Thailand/1(KAN-1)/2004 (H5N1) AIV. IFA with 293T cells transfected with hemagglutinin H5N1 recombinant plasmid was used to confirm MNA. MN Ab titers ≥ 40 were considered to be a positive result.	No information.	Results obtained from 901 participants suggested that clade 1 IAV (H5N1) strains circulating in Thailand among backyard poultry during 2004 did not transmit easily to the study population.
[46]	Thailand, 2005-2008	PF / HP H5N1	No	Acute-phase sera were obtained within 1 week of symptom onset, convalescence-phase sera were obtained >14 days after the acute-phase specimens were collected. Serum samples were tested for H5 specific Ab using MNA for H5N1; reactive samples were confirmed by WB using H5-transfected 293T cells as the test antigen. A/Thailand/1(KAN-1)/2004 (H5N1) and A/Thailand/Nong Bua Lumpoo 1 (PT)/2006(H5N1) were used as Ag in MNA.	None of the 6 participants showing low anti-H5 MN Ab titers (<1:80) had influenza vaccination or infection in the previous influenza season. Only 2/236 H5 seronegative subjects had prior influenza vaccination.	Of the 6 poultry exposed workers showing low anti-H5 MN Ab titers, 3 handled sick or dying poultry, 2 were involved in culling apparently healthy poultry in outbreak areas, 1 reported contact with only healthy poultry during routine farming practices. Authors concluded that low anti-H5 MN titers could be interpreted in conjunction with

				According to WHO, anti-H5 MN titer>1 :80 and confirmed by WB was considered as positive result. HIA were performed in all cases with positive low MN titre anti-H5, to detect cross-reactivity with circulating Ab after prior human influenza virus infection.		plausible poultry, environmental and human exposure to H5N1.
[47]	Peru, 2006	Large PIn (chickens)/ AIV	No	Serum samples were tested by MNA for Ab to the nine prototypic strains of LPAIV from H4 through H12: A⁄Duck ⁄Cz⁄1⁄56(H4N8), A⁄Chucker⁄MN⁄ 14591-7⁄98(H5N2), A⁄Turkey⁄MA⁄ 65(H6N2), A⁄Turkey⁄VA⁄4529 ⁄02(H7N2), A⁄Turkey⁄Ontario⁄ 68(H8N5), A⁄Turkey⁄MN⁄ 38391-6⁄95(H9N2), A⁄Chicken⁄ Germany⁄49(H10N7), A⁄Duck⁄ Memphis⁄546⁄76(H11N9), and A⁄ Duck⁄Alberta 60⁄76(H12N5). All serum samples screened as positive at a dilution of 1:10, were full titered by MNA. Serum samples were tested for human H1N1 and H3N2 influenza virus exposure (A⁄New Caledonia⁄20⁄99 (H1N1), A⁄Nanchang⁄933⁄95(H3N2), and A⁄Panama⁄2007⁄99(H3N2)) using a guinea pig RBC-based HIA.	The use of protective equipment (gloves, mask, apron, or glasses) was low. Only 25.2% of the exposed population reported at least sometimes using protective equipment. None of the subjects reported receiving influenza vaccine.	No evidence of previous AI infection among Peruvian poultry workers was found in this first cross-sectional seroprevalence study performed in South America.
[48]	Nigeria, 2006	PF, PM, Lab (chickens, other bird species) / HP H5N1	Yes. Specimens collected from poultry at 6 out of 117 farms with suspected H5N1 infected poultry were tested for influenza A (H5)	All serum samples were tested by both MNA and horse RBC-based HIA for the presence of Ab to HP H5N1 virus. The MNA used A/chicken/Nigeria/246/06(H5N1) AIV; the HIA used A/chicken/Nigeria/42/2006(H5N1) AIV. A subject was considered to be H5N1 seropositive if MN titers of ≥ 1:80 were	Among PW: 14% always wore masks when working with live poultry; 9% always wore gloves when touching live poultry; 85% always washed their hands with soap after touching poultry.	No serological evidence of H5N1 virus infection was identified among participants, despite widespread exposure to poultry likely infected with H5N1 virus (with a median of 14 days of exposure), minimal use of PPE and lack of antiviral chemoprophylaxis during exposure.

			by RT-PCR, and all were positive.	detected with confirmation by the horse RBC-based HIA.<br>To control for specimen quality, all sera were also tested by MNA with a virus derived from the human influenza A/New York/55/2005, H3N2 virus.	Among LW: 2% and 4% always wore gloves and masks, respectively; 96% always washed their hands after handling suspected H5N1 virus in culture or in poultry specimens; 4% always wore gloves and masks and washed their hands after work; 7% never wore gloves or masks or washed their hands after work.	
[49]	Turkey, 2006	PF (chickens) / HP H5N1	No	Serum samples were tested by HIA (HI titer >20 was considered positive) and ELISA. An MNA was performed against the HP A/Turkey/13/06 H5N1 strain (MN titer > 10 was considered positive) on all samples with Ab detected by either ELISA or HIA, as well as on 25 samples randomly chosen from those that were negative by HIA.	No information.	This survey showed minimal subclinical H5N1 infection among contacts of human cases or infection due to close contact with infected poultry in Turkey in 2006.<br>This low rate of subclinical infection support the reported low infectivity of the virus.
[50]	Germany, 2006	Wetland WLH (wild birds) / HP H5N1	No	Serum samples from 78/97 subjects were tested by plaque neutralization assay (PNA) and MNA for Ab against reference HP virus strain A/whooper swan/R65-2/Germany/2006(H5N1).<br>Reactive sera were reanalysed by MNA using the reference virus strains A/bar-headed goose/Qinghai/1A/2005(H5N1) and A/whooper swan/Mongolia/244/2005 (H5N1).	To evaluate adherence to use of protective measures, a PPE-score was constructed.<br>Of 97 participants 12 (13%) reported having always worn all PPE-devices during bird collection (PPE-score: 9). Adherence	Gaps and variability observed in adherence to use of PPE demonstrated the risk of exposure to AI during wild bird collection, justifying serological testing and regular training of these subjects.<br>For this reason, workers potentially involved in bird collection during wild bird outbreaks should be identified in advance and only the vaccinated

				Serum samples were considered reactive by PNA or MNA with anti-H5 titre> 1:20. MNA was also performed using reference virus strains A/New Caledonia/20/99(H1N1) and A/Wisconsin/67/05(H3N2) in order to analyze possible cross-reactive Ab to human influenza A/H1N1 and A/H3N2.	to PPE use differed between firemen (mean PPE-score: 6.6) and government workers (mean PPE-score: 4.5). The proportion of personnel always adherent to wearing PPE was lowest for masks (19%). 42 participants reported receipt seasonal influenza vaccination from Jul. 2005 to Feb. 2006.	against seasonal influenza should be admitted participating in this collection activities.
[51]	Japan, 2006	H5-AI-free PF, geographic area where an H5N2 outbreak previously occurred / H5N2	No	Serum samples were tested by MNA and by a horse RBC-based HIA for Ab against the LPAIV A/chicken/Ibaraki/1/2005 (H5N2) (titers >40 were considered positive).	No information.	The results suggested that inhabitants and PW living in Ibaraki prefecture might possess higher levels of MN Ab or HI Ab against H5N2 when compared to a Japanese healthy population.
[52]	England 2006	WpE(L/D)P (chickens) / LP H7N3	No	Serum samples (available from 91 persons: 33 acute- and convalescent-phase pairs, 49 acute-phase, 9 convalescent –phase) were screened by MNA and HIA for Ab against the H7N3 LPAIV obtained from the index case patient. MN titers >20 were considered as evidence of seroreactivity. When either test was positive, confirmatory WB was performed	The following PPE were self -reported as "always used" by persons under study: protective coveralls by 81%, protective footwear by 82%, disposable gloves by 67%, face-fitted mask by 51%, other mask by 24%, and protective goggles by 19%. Of 103 persons, 56 (54%) reported complete use of PPE.	The results obtained indicated that the use of PPE could have protected the potentially exposed subjects to infected poultry materials during the H7N3 AIV outbreak.

[53]	China, 2006, 2008	PF HP H5N1 poultry outbreaks areas; PMePrP; SH (chickens, ducks, geese, other birds) / H9N2 and H7 AI	No	Serum samples were tested by horse RBC-based HIA for Ab against A/African Starling/ England-Q/938/79 (H7N1) LPAIV and A/Chicken/ Shanghai/10/01(H9N2) (titer >1:160 was considered as positive).	The workers were required to wear respirators, gloves and protective clothing when they were exposed to poultry. However, the use of gloves and respirators was inconsistent and infrequent.	The study demonstrates H9 seropositivity in northern China, with no documented clinical cases of H9 infection reported. Co-circulation in these regions of the two influenza subtypes, H5 and H9, underlies a risk for virus reassortment.
[54]	USA (Alaska), 2007-2008	WLH (wild birds) / HP H5N1	No	Serum samples were tested by MNA for Ab against A/ Whooper swan/Mongolia/244/2005, a clade 2.2.1 HPAI H5N1 influenza virus (positive titer ≥40), and HIA (positive titer ≥80) or WBA used as confirmatory tests.	A small percentage of RSBHu or USpHu reported wearing rubber gloves while hunting birds (lower among subsistence versus sport hunters, 6% versus 13%). A similar percentage of RSBHu and USpHu reported washing hands during or after hunting (44% versus 38%). Only one hunter reported wearing a facemask while hunting birds. WLB who hunted did not differ from other groups in their low reported use of gloves and hand washing while hunting birds, but during their professional duties	Results were obtained from Alaskan residents with exposure to wild birds at a crossroads of intercontinental migratory flyways. Predominant source of exposure was wild birds, as very few persons in any exposure group reported poultry exposures. USpHu had a longer median duration of exposure (31 years) than RSBHu (13 years) or WLB (15 years). Both subsistence and sport hunters reported similar median days per year of contact (16 versus 14, respectively) and median number of birds handled per day when hunting (5 versus 4, respectively). WLB reported more days of wild bird contact per year (median 21 days) and more birds handled per day (median 20 birds). Many WLB also reported hunting wild birds (40%) in addition to their professional exposure.

					62% reported wearing gloves while handling birds and 89% reported washing hands after handling birds.	
[55]	USA (Iowa, Illinois), 2007-2008	BaPF, large PIn, PMePrP (turkeys, chickens, wild birds, swines) / AIV	No	Serum samples from 170 of 177 participants were tested by MNA for Ab against the following H4, H5, H6, H7, H8, H9, H10, H11 LPAIV: A/Duck/CZ/1/56 (H4N6), A/Chucker/Minnesota/14591-6/95(H5N2), A/Turkey/Massachussets/65 (H6N2), A/Turkey/Virginia/4529/02 (H7N2), A/Turkey/Ontario/68 (H8N4), A/Turkey/Minnesota/38391-6/95 (H9N2), A/Duck/Memphis/546/76 (H11N9). Sera were tested in duplicate and considered positive if titres were positive at ≥1:10 dilutions. Ab titres against human influenza viruses A/New Caledonia/20/99 (H1N1) and A/ Panama/2007/99(H3N2) were determined using a guinea pig RBC-based HIA, (titer ≥1:40 was considered positive)	The use of gloves, masks, aprons, boots and eye protection were studied: none of these devices was associated with serological outcomes. Fewer turkey workers reported receiving a seasonal human influenza vaccine in the previous years than non-exposed controls (42%, 43%, and 58% among TG, TMePr and controls respectively). The use of such vaccine was the lowest among confinement growers (33.3%).	**Occupational exposure to turkeys represents a risk factor for infection with AI especially** among workers operating **in small-scale farms**, using **backyard or free-range poultry growing practices**.
[56]	Indonesia, 2007	Small and large CoPF (chickens, wild birds, pet birds, cats, dogs, fish) / HP H5N1	No	Each serum sample was divided into two aliquot and each of them was double tested by standardized methods: modified HIA for Ab to A/H5N1 virus (A/Ck/Banten/05-1116/05, H5N1) and NT using A/H5N1/Indo/05/IBCDC-RG. H5N1 seropositivity was defined as having two NT titers ≥80 and two HIA	PPE were scarcely used: masks were always worn by 14.1% and sometimes by 32.1% of participants. 11/495 persons (2.2%) had influenza vaccination.	Although a limited exposure of the farmers to H5 virus may have occurred (many participants had poultry manure on clothes after a workday). no serological evidence of infection with avian influenza A/H5N1 virus was found among these workers.

				titers ≥160, as well as two positive independent tests.		
[57]	USA (participants recruited from 3 annual meeting of ornithologists), 2008, 2009, 2010	WLH (Passerines Waterfowls, Shorebirds, Raptors, Charadriiformes, Piciformes, Psittaciformes, Apodiformes, others) / H5N2, H7N2, H7N3, H9N2	No	Serum samples were tested by MNA (titer ≥1:40 was considered positive) for the following Ab against A/Chukar/Minnesota/14191-7/98 (H5N2), A/Turkey/Virginia/4569/02 (H7N2), A/Turkey/Germany/49 (H9N2) in 2008, A/Nopi/Minnesota/07/462960/02 (H5N2), A/Blue-winged teal/Ohio/07 (H7N3) in 2009, A/Turkey/Minnesota/38391-6/95 (H5N2), A/Virginia/4529/02 (H7N2), A/Mexico/4108/09 (H1N1) in 2010.	No information.	The seropositive participant did not report occupational or recreational contact with poultry and had worked exclusively with wild migratory birds in the eastern US, which is a region where H5N2 circulates in migratory waterfowl. Though rare, the transmission of AIV from migratory birds to US-based BHa has potentially significant public health and economic consequences.
[58]	Nigeria, 2008-2010	PIn, open BaPF and LBM (chickens, ducks, geese, turkeys, pigeons) / AIV	No	All sera were tested by MNA for Ab against AIV belonging to the following LPAIV: A/Migratory duck/Hong Kong MPS180/2003 (H4N6), A/Chicken/Nigeria/2007/1132123(H5N1), A/Nopi/Minnesota/2007/462960-2(H5N2), A/Teal/Hong Kong/w312/97(H6N1), A/Water fowl/Hong Kong/Mpb127/2005(H7N7), A/Migratory duck/Hong Kong/MP2553/2004(H8N4), A/Hong Kong/1073/1999(H9N2), A/Migratory duck/Hong Kong/MPD268/2007(H10N4), A/Chicken/New Jersey/15906-9/1996(H11N1), A/Duck/Alberta/60/1976(H12N5), avian-like H9N2 influenza virus and HP H5N1 subtypes. MN titer≥1:10 were chosen as evidence of previous infection with an AIV strain.	The majority (88%) of animal-exposed subjects reported never wearing gloves, but 69% did report always washing their hands. Eye protection and masks were never used by most participants (95% and 88%, respectively). Among all participants, only 1 poultry exposed subject reported having received vaccination for human influenza.	Data obtained from participants showed modest evidence of previous infection with three avian-origin influenza viruses (H5N1, H5N2, and H11N1) and one avian-like H9N2 influenza virus, with eight (2.4%) of animal-exposed subjects and two (3.7%) unexposed subjects having elevated MNA Ab . Considerable poultry exposure was reported by seropositive subjects.

				Potential confounding was controlled by HIA testing sera for cross-reacting Ab to human seasonal A/Brisbane/59/2007(H1N1), A/Brisbane/10/2007(H3N2) and the 2009 pdm A/Mexico/4108/2009(H1N1) influenza viruses (HI titers ≥1:40 accepted as evidence of previous human influenza virus infection or vaccination).		
[59]	China (Shanghai), 2008-2010	WM (chickens, ducks, geese, pigeons) / H9N2	Yes. From 2008 to 2010, 239 H9N2 isolates were obtained from 9297 tracheal and cloacal paired swabs (positive rate 2.5%) collected from live poultry. H genes of seven H9N2 virus isolated were sequenced.	Serum samples from participants were tested for Ab against AIV H9 by chicken RBC-based HIA (titers >1:40 were considered positive). MNA for Ab against A/Chicken/Shanghai/0734/2007, A/Chicken/Shanghai/0817/2008, and A/Chicken/Shanghai/0867/2008 LP H9N2 virus strains was performed (titers >1:20 were considered positive). H1N1 was the most prevalent subtype in humans in Shanghai during the sampling period.	No information	Strong evidence of avian-to-human transmission of H9N2 IAV was provided by serological results. H9 HI Ab positive rate in WMW was significantly higher than that in the controls H9 HI Ab positive rate detected in the controls suggests possible acquisition of H9N2 asymptomatic infection by routine contact to birds.
[60]	China (Guangdong, Zhejiang, Fujian, Jiangxi), 2008-2012	SF ponds (wild and domestic avian species, pigs) / HP H5N1	Yes. 1980 pig sera, collected from Mar. 2009 and Mar. 2013 and tested as performed for the 1606 human samples, were all negative for Ab to A/chicken /Guangdong/178/04 (H5N1) clade 2.3.2	Serum samples were tested by a horse RBC-based HIA for Ab against HPAIV A/chicken /Guangdong/178/04 (H5N1) clade 2.3.2; NT was used as confirmatory assay (HI and NT titer ≥ 80 were considered as positive). To rule out non-specific cross-reactivity, all 1710 human sera were titrated against H1N1 and H3N2 seasonal influenza viruses.	No information.	Despite environment and lifestyle on swine farms in southern China provided many opportunities for wild aquatic birds, domestic poultry, pigs, and humans to come in close contact, serological evidence of transmission of HPAI H5N1 strain (clade 2.3.2) seems to be very low.
[61]	Italy, 2008-2010	InPF (gallinaceous species) / LP H5N2, H5N7, H7N3, LP and HP H7N1	No	Serum samples were tested by a horse RBC-based HIA for Ab against H5 and H7 LPAIV: A/Duck/It/4445/07(H5N2);	No information.	The study provided additional support for the low transmissibility of H7 AIV to humans.

				A/Turkey/It/2369/09(H5N7); A/Turkey/It/ 218-193/10(H5N2); A/Chicken/It/3775/99(H7N1); A/Turkey/It/214845/03(H7N3); A/Duck/It/332145/09(H7N3). HI positive results were confirmed by MN-ELISA. Cut-off titer of ≥1:10 were chosen for both serologic assays.		
[62]	Bangladesh, 2009	PF, wholesale and retail LBM (chickens, ducks, geese, pigeons, quail) / HP H5N1	No	Serum samples were tested by MNA for Ab to the HPAI H5N1 strain A/Bangladesh/207095/2008, clade 2.2.2 (titer >40 was considered as positive). Positive results were confirmed by a horse RBC-based HIA using the above virus or by an H5-specific WBA using a recombinant H5 (clade 2.2) protein as Ag.	93% of PW from farms who participated in poultry culling used PPE during culling, and 99% received post-exposure oseltamivir. More than half of these PW used protective measures during daily poultry care.	91% (193/212) of PW from farms and 85% (178/210) LBMW had direct contact with poultry that died during a confirmed HPAI H5N1 poultry outbreak in the farm or with market poultry die-offs (from suspected HP H5N1 AI) in the market. Seronegative results detected in PW could be explained by the long median time interval between onset of poultry die-offs on farms and collection of blood specimens from farm workers (444 days, ranging from 22 to 543) which might have reduced the ability to detect H5N1 Ab, and/or by the protective measures used in farms during culling and daily activities, able to reduce exposure to HPAI H5N1 virus.
[63]	Mongolia, 2009	AgA (horses, camels, goats, sheep, cattle, pigs, domestic poultry) / AIV including HP H5N1	No	MNA was used to detect in serum samples Ab against a panel of: n.1 Mongolian H3N8 equine influenza virus (A/Equine/Mongolia/01/2008(H3N8); n.1 avian-like (A/Hong Kong/1073/1999(H9N2) and n.6 avian LPAIV and n.1 HPAIV: A/Migratory duck/HongKong	17 participants (4.0%) reported having previously received a seasonal influenza vaccine, with 5 participants receiving vaccines within a year of study enrollment.	Seroreactivity was sparse among participants suggesting little human risk of zoonotic influenza infection.

				MPS180/2003(H4N6), A/Nopi/Minnesota/2007/462960-2(H5N2), A/Teal/Hong Kong/w312/97(H6N1), A/Waterfowl/Hong Kong/Mpb127/2005(H7N7), A/Migratory duck/HongKong/MP2553/2004(H8N4), A/Migratory duck/HongKong/MPD268/2007(H10N4) and A/Cygnus/Mongolia/3/2009(H5N1)HPAIV (titers≥1:10 were considered positive).<br>Cross-reactions from previous infection with human influenza viruses (A/Brisbane/59/2007(H1N1), A/Mexico/4108/2009(H1N1), A/Brisbane/10/2007(H3N2)) controlled by HIA for Ab against 3 human influenza viruses (titers≥1:40 were considered positive).		
[64]	Iran, 2009	PF, SH (poultry) / H9N2	No	Serum samples were tested by a turkey RBC-based HIA for Ab against H9N2 AIV.<br>Samples were considered negative if titer were =1/8.<br>Positive samples had at least 1 serum sample with titer >1/8 or at least 3/15 with titer =1/8.	No information.	No significant variation among PFW, SHW and vets, although between these 3 groups and the other 2 (patients with clinical signs of respiratory diseases and normal general citizens) significant variation was observed. Higher prevalence in PFW, SHW and vets possibly enabled by close and frequent contact with H9N2 virus, endemic in Iranian poultry farms.
[65]	Bangladesh, 2009-2010	LBM (chickens, ducks, geese, quail) / HP H5N1	Yes.<br>Routine poultry surveillance identified H5N1 at	Serum samples were tested by MNA using HP H5N1 clade 2.2 (A/Bangladesh/3233/2011) (titer >40 was considered positive).	Low PPE use was reported.	Results suggest that human infection with H5N1 among heavily exposed workers at LBM occurs infrequently.

			11/12 (92%) LBM and in 25/27 monthly samples.	WBA against recombinant hemagglutinin A/bar-headed goose Qinghai/1A/2005 (clade 2.2) HP H5N1 was used as confirmatory test. Seroconversion was defined as detection of a >4 fold rise in MN Ab titer between the initial serum sample and a paired second serum sample, with the second sample achieving a titer >40. Positive and seroconverted samples were also tested by MNA and HIA against pandemic A(H1N1) pdm09 virus strain A/Mexico/ 4108/ 2009 (H1N1) to exclude cross-reactivity.		Feeding poultry, cleaning feeding trays and water containers, not washing hands after working with sick poultry, cleaning feces from pens were behaviours classified as high exposure. Slaughtering, defeathering, eviscerating, collecting or transporting feces, stuffing poultry into bags were classified as medium exposure. Frequently performing high-exposure behaviors was associated with 1.5 times higher risk of acquiring H5N1 virus infection compared with performing medium-exposure behaviors.
[66]	Romania, 2009-2010	Large-scale CoSF, BaPF (domestic poultry, wild birds, pigs) / AIV including HP H5N1	No	Serum samples were tested by: guinea pig or turkey RBC-based HIA for Ab against the following 4 human A/Brisbane/59/2007(H1N1), A/New Caledonia/20/1999(H1N1), A/Mexico/4108/2009(H1N1) and A/Brisbane/10/2007(H3N2) and 3 swine (A/Swine/Lutol/3/2000(H1N1), A/Swine/Gent/7625/1999(H1N2), A/Swine/Flanders/1/1998(H3N2)) IAV; MNA for 7 LPAIV including A/Migratory duck/Hong Kong MPS180/2003(H4N6), A/Nopi/Minnesota/2007/462960-2(H5N2), A/Teal/Hong Kong/w312/1997(H6N1), A/Water fowl/Hong Kong/Mpb127/2005(H7N7), A/Migratory duck/Hong Kong/MP2553/2004(H8N4),	Among subjects upon enrollment, 118/306 agriculture workers and 35/51 controls received vaccination for human influenza.	Although Romania experienced multiple incursions of H5N1 HPAI, no evidence of previous human H5 infections was found among 363 adults studied. According to obtained data, the H9N2 virus may have circulated in Romanian poultry and occasionally infected man. In fact, there was no evidence that previous infection with human H3N2 or H2N2 viruses, potentially confounding the H9N2 seroreactivity.

				A/Migratory duck/Hong Kong/MPD268/2007(H10N4), A/Chicken/New Jersey/15906-9/1996(H11N1) and 1 HPAIV A/Chicken/Romania/6059-1TS/2008(H5N1), and 1 avian-like LP A/Hong Kong/1073/1999(H9N2). For avian and swine viruses, titers of ≥1:10 and ≥1:40 were respectively used in MNA and HIA as indication of previous infection or outcome. In multivariate risk factor modelling for AIV, potential confounding from Ab against human viruses (e.g. H3N2 and H2N2) were controlled by creating binary covariates for such viruses (HI titer ≥1:40 counted as positive).		
[67]	China (Beijing), 2009-2010	PF, SH (ducks, chickens)/ HP H5N1, H9N2	No	Serum samples were tested by MNA for Ab to HP A/duck/Huabei/01/2007 (H5N1) virus, belonging to clade 2.3.4, and A/chicken/Shangdong/ZB/2007(H9N2) of the F/98 genotype (titers>80 were considered positive); all results were generated from at least 2 independent assays.	All participants had no history of vaccination for seasonal influenza in the past 3 years. Wearing PPE was not a routine practice: of 207 participants who completed the questionnaire, 112 (54.1%) wore gloves and 95 (45.9%) masks. Moreover, 165/207 (79.7%). routinely washed their hands after work, 174/207 (84.1%) regularly. used disinfectant.	The study showed that a considerable risk for infection with H9 subtypes was found in PW Wearing protective equipment was not common among the participants.

					Chicken keepers were more likely to follow good hygiene practices than duck keepers (p<0.01).	
[68]	China (22 Provinces), 2009-2011	LPM, large-scale PF and BaPF, SH, WBH / H9N2	No	13,236 serum samples were tested by turkey RBC-based HIA for Ab against H9N2 A/Guangzhou /333/99 (G9) and H9N2 A/quail/Hong Kong/G1/97 (G1) Ag; 2464 serum samples were tested for only one of the two Ag (positive titer >40). MNA was used as confirmatory test (positive titer >40).	No information.	The study showed that in China subclinical human infections with H9N2 AIV are largely distributed. Overall, higher seroprevalence of H9N2 antigens were observed in Southern than in Northern China, and this might be caused by a higher poultry density. The lowest seroprevalence detected in wild bird habitat workers was consistent with previous studies.
[69]	China (southern China), 2009-2012	Lakes with wild birds near SF, sometimes including poultry / H9N2	No	Serum samples were tested by HIA for Ab against A/chicken/Guangdong /V/2008(H9N2) LPAIV (titer >1:160 were considered positive). HI positive sera were tested by MNA, performed according to standard protocols, for confirmation.	Among the swine farm residents, the use of gloves and respirators was inconsistent and infrequent.	All positive participants indicated they sometimes bathe or swim in swine farm ponds shared with waterfowl and reported close poultry contact. The risk of AIV infection among swine farm residents might be greater through contact with outdoor-reared waterfowl and wild birds than with indoor commercial or industrial poultry where biosecurity and swine farm workers' protection are generally higher.
[70]	Egypt, 2010-2013	BaPF, LBM (chickens, ducks, geese, pigeons, turkeys, pet birds)/ H7 AI	No	Serum samples were tested by VMN assay for Ab against A/Netherlands/219/2003 (H7N7) LPAIV (positive titers ≥80), WB and IF assays were used as confirmatory tests	No information.	Serological data suggested that PG were exposed to A/H7 viruses. No significant difference was detected at any year. A/H7 viruses may be circulating in Egyptian poultry albeit at a lower frequency than the

						endemic H5N1 and H9N2 viruses.
[71]	China (Jiangsu Province), 2010	BaPF near waterbird habitats / HP H5N1	No	Serum samples were tested by horse RBC-based HIA for Ab against A/Anhui/1/05 and A/Hubei/1/10 H5N1 HPAI viruses (titer >1:160 was considered positive).	No information.	Birds-to -human transmission of avian H5N1 virus was low. Workers associated with raising larger poultry flocks have a higher risk of seropositivity.
[72]	Lebanon, 2010	BaPF, CoPF (chickens, turkeys, geese, quails, wild birds) / AIV	No	Sera were tested by MNA for Ab to LP A/duck/Hong Kong/365/78 (H4N6), RG-A/turkey/Egypt/7/2007 (H5N1), A/quail/Hong Kong/YU 421/02 (H6N1), RG-A/Netherlands/219/2003 (H7N7), A/turkey/Ontario/6118/68 (H8N4), A/turkey/Israel/1567/04 (H9N2), A/chicken/Germany/N/49 (H10N7), A/duck/Hong Kong/P50/97 (H11N9), A/duck/Alberta/60/76 (H12N5), A/gull/Astrachan/458/85 (H13N6), A/mallard duck/Astrachan/263/82 (H14N5). A/wedge-tailed shearwater/Western Australia/2576/79 H15N9 A/black-headed gull/Sweden/5/99 H16N3 A/Brisbane/59/04 H1N1 A/California/04/09 H1N1 A/Brisbane/10/07 H3N2. (titers ≥1:10 were considered positive). Horse RBC-based HIA was used as confirmatory assay to test sera found to be positive by MNA for the presence of Ab against AIV. To evaluate potential cross-reactivity, human H1N1, H3N2 seasonal and H1N1 pdm influenza viruses exposure was tested by turkey RBC-based HIA.	CoPG used more likely protective masks, footwear, and clothes than BaPG. There was no difference in the use of eye protection and gloves between the two groups.	**Occupational exposure to chicken as risk factor for infection with AI was shown especially among BaPG,** despite CoPG were significantly exposed to more chicken (median = 2000 vs 14 birds) with more hours spent per week (median = 21.0 vs 3.5 hours) than BaPG. In particular, BaPG were found to be infected with H4 and H11 AIV. Farmers with Ab against each virus type clustered in a small geographic area, suggesting that unrecognized outbreaks among birds may have led to these human infections.

[73]	India, 2010	CoPF, wet LBM in urban, semiurban or rural areas (chickens) / H9N2	No	Serum samples were tested by horse RBC- and turkey RBC-based HIA and MNA for Ab against influenza A/Chicken/India /NIV/99321/09(H9N2). In both tests, positive titers were calculated using the three cut-off Ab titres >40, >80 and >160.	Vaccination against seasonal and pandemic flu was not reported by any participants.	This study describes the first seroprevalence report of antibodies against H9N2 AI among poultry workers in India. ILI was not reported by studied subjects in the last 6 months before blood collection, and presence of Ab against H9N2 suggested a probable subclinical infection.
[74]	Pakistan, 2010-2011	CoPF (broiler, layer and breeder chickens) / HP H5N1 and H7N3, H9N2	No	Serum samples were tested by a horse RBC-based HIA for Ab against inactivated HP H5N1 Ag and antiserum (A/Ck/Scot/59), LP H7N7 Ag and antiserum (A/tky/Eng/647/77) and H9N2 Ag and antiserum (A/Turk/Wisc/66) (titer≥ 1:160 were considered positive).	None of the participants was vaccinated with seasonal flu vaccine.	The prevalence of H7 and H9 in poultry farm workers was quite high. The study showed greater risk of H7 infection for PFW who worked at breeder farms, being much larger in terms of total population and space, with breeders reared on a dusty floor with faecal aerosol.
[75]	China (Zhejiang Province), 2010-2012	Large-scale CoPF, urban and rural LBM; BaPF, SH, PMePrP / HP H5N1	Yes. Environmental samples were tested for IAV and H5N1 virus subtype by rRT-PCR. Of 3453 samples, 468 were IAV positive, and 105 (3%) for H5 subtype. Positive rates of samples collected from large scale poultry companies, LBM, poultry slaughtering and processing plants, poultry backyard households and	Serum samples were tested by horse RBCs based HIA for Ab against HP H5N1 viruses [A/Hubei/1/2010 (H5N1) and A/Anhui/1/2005 (H5N1)]. HI titers ≥1:160 were considered positive. All positive samples and 5% of the negative samples were confirmed by MNA.	No information.	In the present study, breeding more than 1,000 birds and direct or close contact with poultry in the workplace or in ther environment could represent potential risks of H5 AIV infection.

			wild migratory bird habitats were 25.7%, 68.6%, 5.7% and 0% respectively, with significant difference. Surface of cages showed the highest H5 IAV prevalence.			
[76]	South Africa, 2011-2012	OF (ostriches) / HP H5N2, LP H7N1	No	Sera were tested by HIA against reference H5 and H7 antigens and autologous inactivated antigens obtained after the 2011 and 2012 ostrich outbreaks of HPAI H5N2 and LPAI H7N1. HIA were performed with both turkey and horse RBC for H5N1 and H7N1 strains.<br>1st survey (H5N2 outbreak): sera were screened by HIA for H5 and H7, with HI titers >20 confirmed by MNA.<br>2nd survey (H7N1 outbreak): sera were screened by HIA for H5 and H7, with HI titers >10 confirmed by MNA.<br>3rd survey (control group) all vets were screened by HIA for both H5 and H7, with HI titers >20 confirmed by MNA. MNA titers >40 were considered to be likely positive.<br>To evaluate possible cross-reaction, the following human influenza antigens were used: A(H1N1)pdm09 in 2011, and both A(H1N1)pdm09 and A(H3N2) in 2012.<br>The following antigens were used for MNA: A/H5N2/S2011/06_0005/2011 and A/H7N1/S2013/04_169/2013, both AIV originally isolated from ostrich.	Data on use of PPE were only available for the 1st survey: among individuals who tested positive to H7N1, 1 out of 4 reported wearing gloves, 2 out of 4 masks (N95), 0 out of 4 goggles ($P = .23$) when dealing with ostriches in the abattoir.<br>During 1st survey (2011) 52 of 207 participants (23%) were vaccinated against seasonal influenza, including 60% of SHW.<br>For the 2nd survey data were not available.	Humans involved in the control of AI outbreaks in South Africa were at low risk of being infected with influenza A H5N2, but cases did occur.<br>H7 strains seemed to pose a greater risk of infection to AW, vets, and LW. Clinical signs seemed to be limited to conjunctivitis and ILI. H7 positive serum samples, collected from abattoir workers in Aug 2011 before the 2012 H7N1 outbreak was detected, suggested that this LP H7 virus might have circulated undetected for several months in ostriches.

[77]	China (Beijing), 2011	CoDF, BaDF, slaughtering sites (ducks) / H5, H7, H9 AI	No	Serum samples were tested by a horse RBC-based HIA for Ab against A/Chicken/Anhui/01/2005 (HPAI H5N1), A/Chicken/Hebei/02/2007(LPAI H7N2) and A/Chicken/Shanghai/10/1999 (LPAI H9N2). A titer≥1:40 was considered positive.	Overall, 732 and 818 duck-related workers used masks and gloves, respectively. Among 12 H9 seropositive workers, mask was used by 1/12 and gloves by 3/12.	The risk of infection with H5, H7 and H9 viruses appeared to be low among duck-related workers in Beijing, China. However, in this study the seroprevalence of H9 antibodies amongst villagers breeding ducks in their backyard was significantly greater than participants from other categories. Close monitoring of H9 infection should be warranted, especially amongst villagers breeding ducks in their backyards.
[78]	Pakistan, 2011	PIn, LBM (chickens) / H9N2, H7 AI	No	Serum samples were tested by a horse RBC-based HIA for Ab against reference H9N2 Ag (A/turkey/Wisc/66), and inactivated LP H7N7 Ag (A/tky/Eng/647/77). Titers ≥1:160 were considered positive.	No information.	Overall GMT of H9 Ab was higher than that of H7 Ab almost in all cases, indicating that H9 is more endemic than H7 in Punjab province. **The highest H7 and H9 GMT in 7 vaccinators could be attributed to continual handling of killed vaccine of subtypes H7 and H9**. The low seropositivity found in poultry vets (30.4%) could be related to possible adoption of precautionary measures like using mask while visiting poultry farms and using gloves during post mortem examination.
[79]	Viet Nam, 2011	LPM / HP H5N1	No	Serum samples were tested by a horse RBC-based HIA for Ab against HP A(H5N1) viruses clade 1 (A/Viet Nam/HN30408/2005), clade 2.3.4 (A/Viet Nam/HN31244/2007) and clade 2.3.2.1 (A/Viet Nam/CM32/	No information	Seropositivity to influenza A(H5N1) viruses detected in all groups of PMW emphasized the importance of the adoption of preventive measures as PPE and vaccination.

				2011). Samples with HI titers ≥40 were also tested by MNA. A sample was considered positive if an HI titre ≥80 and an MN titre ≥20 were obtained in duplicate with any IAV clade.		
[80]	China (Guangdong Province), 2011-2012	WpE(L/D)P / HP H5N1	No	Serum samples were tested by horse RBC-based HIA for Ab against HPAI A/chicken/Guangdong/178/04(H5N1), and by NT. HI and NA titers ≥80 were considered positive.	All 406 vets were unvaccinated.	The risk of avian to human transmission of the HPAI H5N1 AIV was found to be very low.
[81]	China (Tai'an), 2011-2013	PF, SH, wholesale and retail PM (chickens) / H9N2	No	Serum samples were tested, according to WHO standard procedures by HIA for Ab against A/chicken/Shandong/1/08/H9N2 (titer ≥40 was considered positive). HI positive results were confirmed by MNA (titer ≥40 was considered positive).	All the 1200 subjects had no history of vaccination for seasonal influenza in the past three years.	Data obtained indicated that occupational exposure to chickens was an important risk factor for H9N2 AIV infection.
[82]	China (Shandong Province), 2011-2012	CoPF (chickens, hens) / H9N2	No	Serum samples were tested for Ab against LPAI A/chicken/Shandong/1/08/H9N2 virus by horse RBC-based HIA and by the MNA used for confirmation (titers ≥40 were considered positive for both HIA and MNA).	No participant was vaccinated against any seasonal influenza viruses.	This study identified H9N2 AI infections among PW in Shandong, China. Although no documented clinical cases of H9N2 infection were testified, the possibility of mild or subclinical infections could not be excluded.
[83]	Taiwan, 2012	Live poultry stalls, PF / LP and HP H5N2, H6N1, LP H7N3, H7N9	No	Serum samples were tested by HIA using horse RBC for H5N2 AIV subtype; turkey RBC for H6N1 and H7N9 AIV subtypes; guinea pig RBC-for H7N3 LPAIV subtype. The cut-off Ab titre of seropositivity was 1:80 for H5N2 and 1:40 for H7N3, H7N9 and H6N1 viruses. Antigen strains used included: the avian origin A/chicken/Taiwan/1209/2003 (H5N2) and A/duck/Taiwan/A1741/2011 (H7N3) LPAI strains; the human	The majority of LPV and PFW didn't receive H5N1 and/or seasonal influenza vaccines two years prior to specimen collection. More than 50% of the non-PW received seasonal influenza vaccine in 2010 and/or 2011, the H5N1 vaccination coverage was low.	Occupational exposure was associated with a high risk of AIV infection, and the seroprevalence detected in humans reflected the endemic strains in poultry in this region. Geographical analysis showed that PW whose workplaces were near farms where H5N2 outbreaks occurred had greater risks of being exposed to this AIV virus.

				origin A/Taiwan/2/2013(H6N1) and A/Taiwan/1/2013(H7N9) strains.	12.5% of PFW didn't use PPE compared to 2.1% of LPV PPE mainly used were gloves, boots and masks.	
[84]	Iran, 2012-2013	PF, SH, university poultry hospital / H9N2	No	According to WHO procedures, serum samples were tested by a chicken RBC-based HIA and by MNA for Ab against two Iranian LPAIV H9N2 isolates representatives of sub-lineages A (A/chicken/Iran/12VIR/9630/1998) and B (A/chicken/Iran/10VIR/854-5/2008) (titers ≥40 were considered positive). Sera of all individuals were also tested for the presence of Ab to seasonal A/Minnesota/11/2010 H3N2 and A/California/4/2009 H1N1pdm 2009 viruses.	None of the poultry exposed workers had been vaccinated against flu.	The study demonstrated that exposure to H9N2 AIV had occurred in humans in Iran. Statistical analyses models showed that exposure to poultry significantly increased the risk of infection with H9N2 AIV.
[85]	China (Guangdong Province), 2012, 2013, 2014	Wholesale LPM, meat markets or retail LPM, BaPF, PF / HP H5N1, HP and LP H7N7, H7N9, H9N2	No	Serum samples were tested by protein microarray to detect binding Ab to 13 different haemagglutinin (HA1-part) of the following H5, H7, and H9 AIV antigens: n. 5 H5N1 (A/Vietnam/1194/2004, A/Anhui/1/2005, A/Cambodia/R0405050/2007, A/Hubei/1/2010, A/Egypt/N03072/2010); n. 1 H7N7 (A/Chicken/Netherlands/1/03); n. 2 H7N9 (A/Anhui/1/13, A/Shanghai/1/13); n. 5 H9N2: (A/Chicken/Hong Kong/G9/97, A/Guinea fowl/Hong Kong/WF10/99, A/Hongkong/1073/1999, A/Hong Kong/33982/2009, A/Chicken/India/IVRI-0011/2011).	Data on human seasonal vaccination in the past year were available for PW.	Ab titres to the most recent H9 strain (A/Chicken/India/IVRI-0011/2011) were significantly higher for PW from all age groups, suggesting high infection rate with this recent H9 AIV in this occupational group. 7% of PW had titres above 80 for an H5N1 Ag in combination with a PW specific H5 Ab profile, suggesting a substantial rate of mild or asymptomatic H5N1 infections in at-risk populations in Guangdong. For H7, Ab prevalence was remarkably low and limited to PW from two LPM.

				Each serum was tested in four-fold dilutions starting from 1:20. Titers smaller than a serum dilution of 20 were set to 10, and titers higher than a serum dilution of 1280 were set to 1810.		
[86]	Cambodia, 2013	LBM (chickens, ducks) / HP H5N1, H9N2, H7N9	Yes. Poultry and environmental surveillance for A/H5N1 was done by collecting 1048 animals and environmental samples. During each market investigation: tracheal and cloacal swabs were collected from 4 randomly selected poultry (3 ducks and 1 chicken). Environmental samples were collected in the same cage/site where poultry swabs were taken. 45% of all samples were IAV positive by qRT-PCR. H5N1 virus was detected in 79% of samples positive for IAV and in 35% of all samples collected. Molecular analyses	Serum samples from 111 LBMW were tested by HIA and MNA for Ab against: AI A/H5N1 clade 1.1.2 reassortant viruses (A/Cambodia/X0121311/2013 and A/Cambodia/X0125302-/2013) isolated from human cases during 2013; influenza A/H9N2 virus (A/Environ ment/Cambodia/E265/2013) isolated from LBM during 2013; A/H7N9 (A/Anhui/01/2013). Virus exposure was confirmed with HI titer >80, and MN titer >40.	No information	The study documented an intense co-circulation of influenza A/H5N1 and LPAI viruses in poultry and environmental samples from Cambodian LBM during 2013. As no LBMW reported any symptoms in relation to acute AI infection, seroconversions observed to A/H5N1 and A/H9N2 AIV would most likely be related to sub-clinical or very mild cases.

			showed that only A/H5N1 clade 1.1.2 reassortant viruses were detected during 2013. In addition, at least 9 LPAIV from H1, H2, H3, H4, H6, H7, H9, H10 and H11 subtypes co-circulated in birds.			
]87]	China (Guangdong Province), 2013	PF, LPM, pet birds exposure areas / H10N8	No	Serum samples were tested by horse RBC-based HIA against A/Jiangxi-Donghu/346-1/2013, H10N8 virus, generated via RG (titer >1:20 was considered positive). HI positive results were confirmed by MNA (titer >1:80 was considered positive).	No information	No serological evidence for human infection with the novel avian-origin influenza A (H10N8) virus was detected in 400 vets from China during the Feb.-Aug. 2013 period.
[88]	China (Guangdong Province), 2013	SF, PF, retail PM, SH, zoo, veterinary structures / H10N8	No	827 serum samples were screened by a horse RBC-based HIA for Ab against influenza virus A/Jiangxi-Donghu/346-1/2013(H10N8). Sera with HI titers ≥1:20 were further tested with MNA against the same virus. HI titers > 1:40 was indicative of possible evidence of previous infection; both HI and MN titers ≥ 1:40 were indicative of probable evidence of previous infection.	Non-animal exposed subjects reported no history of having received an influenza vaccine.	Results of the study suggested that animal workers may have been infected with the H10N8 virus before the first recognized H10N8 human infection case (Nov. 2013).
[89]	China (Jiangxi and Henan Provinces), 2013-2014	CoPF, small-scale CoPF, LPM and wholesale market / H7N9	No	Serum samples were tested by a modified HIA with horse RBC for Ab detection against IAV H7N9, A/Anhui/1/2013 strain (titer ≥20 was considered positive). A modified MNA was used as confirmatory test (titers ≥20 or four-fold increases in paired sera were considered positive). Paired samples were collected from 47 (77%) of the healthcare contacts and	Of the 2 MNA A(H7N9) positive PW, one reported routine protection with gloves, mask, rubber overshoes; the other with gloves and rubber overshoes	There was no evidence of widespread transmission of influenza A(H7N9) virus during the 2013-2014 studied period, although A(H7N9) may have caused rare, previously unrecognized infections among PW.

				from 84 (72%) of the non-healthcare contacts.		
[90]	China (Guangdong Province), 2013-2014	SF, PF, LPM (including pigs), pet veterinary structures (chickens, ducks, geese, companion animals) / HP H5N1, H9N2, H7N9	No	Serum samples were tested by a horse RBC-based HIA for the presence of Ab against 7 human and animal IAV including: A/Guangdong/1057/2010(H1N1), A/swine/Guangdong/L6/2009(H1N1) [classical swine virus, CS/H1N1], A/swine/Guangdong/SS1/2012 (H1N1) [Eurasian avian-like swine virus, EA/H1N1] A/duck/Anhui/1/2006(H5N1), [AIV/H5N1] A/chicken/Shanghai/10/2001(H9N2) [AIV/H9N2], A/pigeon/Shanghai/S1421/2013(H7N9) [AIV/H7N9] and A/canine/Guangdong/2/2011(H3N2) IAV. To examine potential confounding factors through cross-reactivity, HI titers from control antisera were determined against a panel of rabbit antisera directed against 3 human, 2 swine, 3 avian and 1 canine IAV.	LPM workers were daily exposed to both poultry and pigs, with no use of PPE. All the participants denied having previously received annual influenza vaccines.	Compared to the control group LPMW were at a higher risk of infection with 3 subtypes of avian influenza, H5N1, H7N9, and H9N2. PFW and LPMW had higher antibodies positive rate against H7 and H9 viruses, when compared to non-animal workers. Though the number of H5-positive PFW and LPMW were slightly higher than other groups, no significant differences were observed. The obtained results suggested that animal exposed subjects were more likely to have Ab against animal influenza viruses, although partial confounding by cross-reactive Ab against human viruses or vaccines cannot be ruled out.
[91]	China (Hong Kong), 2013, 2014	LPM (wholesale markets or poultry retail markets), pig/cattle SH (chickens, ducks, pigeons, geese) / HP H5N1, H7N9, H9N2,	No	Serum samples were tested by turkey or horse RBC-based HIA for Ab against A/Vietnam/1194/2004(H5N1), A/Anhui/1/2013 (H7N9) and A/Hong Kong/ 1073/99(H9N2). Turkey RBC were used in HIA for all 3 AIV, horse RBC were also used for A(H7N9) (titer $\geq$40 were considered positive for H5N1 and H9N2, titer $\geq$ 160 were considered positive for H7N9). Seroconversion occurred with a 4-fold increase in HI titer from 2013 to 2014. HIA for human A(H1N1) and	From 2009 to 2012 the percentage of influenza vaccinated workers ranged between 33.3%-40.0% and 20.3%-44.4% in LBM and SH workers, respectively.	For H5N1 virus, none of the serum samples from LPM/SH workers collected in 2013 had an HI titer of ≥40. **If an HI titer ≥40 was used as cutoff H5N1 seropositive rate was significantly higher for LPMW than that of SHW in 2014 (37.8% [17/45] vs 3.7% [1/27])** and the seropositive rate of LPMW were significantly higher in 2014 than in 2013 (37.8% [17/45] vs 0% [0/30]. If an HI titer ≥80 was used

				A(H3N2) viruses were also performed for individuals with paired serum available (virus strains used: A/Hong Kong/415742/2009(H1N1) and A/HK/460611/2013(H3N2). Turkey and guinea pig RBC were used in A(H1N1) and A(H3N2) HIA, respectively.		as cutoff, there was atrend towards higher A(H5N1) seropositive rate among LPMW in 2014 than in 2013 (11.1% [5/45] vs 0% [0/30]. For H9N2 virus, 10% (3/30) of LPMW and 8.7% (6/69) of SHWs had an HI titer ≥40 in 2013. Between 2013 and 2014, the HI seropositive rate of LPMW significantly increased from 10% to 55.6% if a cutoff of 40 was used ($P < 0.001$). There was no significant increase in seropositive rate from 2013 to 2014 for SHW irrespective of the cutoff used. **H9N2 seropositive rate was significantly higher for LPMW than that of SHW in 2014 if the cutoff HI titer for seropositivity was 40 (55.6% vs 14.8%) or 80 (22.2% vs 3.7%).** For A(H7N9) virus, WHO recommended the use of horse RBC in HIA, with a seropositivity cutoff of 160. When horse RBC was used, 10% (3/30) of LPMW and 13% (9/69) of SHW had an HI titer of ≤160, but there was no significant difference in the horse erythrocyte HI seropositive rates between 2013 and 2014 for both groups. This study provided serological evidence of subclinical human infections due to AIV, especially related to A(H5N1) and A(H9N2) subtypes, among LPMW in

						Hong Kong, emphasizing the importance of longitudinal and regular surveillance of LPMW.
[92]	China (Beijing), 2013-2015	Commercial or private PF and SF, SH / H7N9 and HP H5N1	No	Serum samples were tested by HIA for Ab against H7N9 and H5N1 virus Ag using 1% horse RBC. Antigens used: vaccine strains of A/Anhui/1/2013 (H7N9) (NIBRG-268), A/Anhui/01 /2005(H5N1) PR8-IBCDC-RG5 (clade 2.3.4), and A/Hubei /1/2010 (H5N1) (clade 2.3.2.1). Titers of ≥ 1:80 were considered positive. Seroconversion was defined as a four-fold or greater increase in Ab HI titer between paired serum specimens with a titer ≥ 1:40 for the second specimen.	No information	Results obtained showed that the risk of H7N9 and H5N1 virus infections among PW, SW and the general population remained low since 2013, as indicated by the seroprevalences of Ab in the 3 cross-sectional surveys and the incidence rates of infections in the cohorts. Moreover, **PW rather than SW were at a higher risk of contracting H7N9 and H5N1 clade 2.3.4 viruses compared with the general population.** The risks of H7N9 and H5N1 virus infection remain low in poultry workers, swine workers and the general populations in Beijing, China. Importantly, the risk posed by H5 viruses appears not lower than that of H7N9 viruses.
[93]	China (Jiangsu Province), 2013-2016	LPM including pigs (chickens, ducks, geese)/ H7N9, H9N2, HP H5N1 and H5N6	Yes. 3121 poultry and environmental samples, collected from 9 LPM, were examined by PCR, viral isolation and sequencing. A total of 466/2010 (23.2%) cloacal swabs, 145/590 (24.5%) environmental swab samples, and 115/521 (22.0%)	Serum samples were tested by HIA for Ab against the human H7N9 isolate (A/Jiangsu/Wuxi 05/2013), clade 2.3.4.4 H5N6 virus (A/chicken/Jiangsu/WXBING2/2014), clade 2.3.2.1c H5N1 virus (A/chicken /Jiangsu/WX927/2013), and Y280-like H9N2 virus (A/chicken/Jiangsu/WXWA021/2013) (titer ≥10 was considered positive). 1% horse RBC were used in HIA performed with H7N9, H5N6, H5N1 viruses, whereas for the H9N2 virus 0.5% turkey RBC were used.	During the study period, minimum - maximum. percentages of vaccinated participants were: 0.4%-2% in PW, 0.4%-2.9% in SW, and 0.1%-1.6% in controls.	**In total, PW had relatively higher seroprevalence and seroincidence of H7N9, H9N2, and H5N1 than SW and general population,** although the overall seroprevalence and seroincidence was low. No PW was positive for H7N9 in the 2016 survey and for H5N1 in the 2014 and 2015 surveys. **In PW the highest seroprevalence of H5N1 (3.46%) was found in 2016**

			fecal/ slurry specimens were positive for IAV. Active surveillance for AIV revealed that 10 subtypes were circulating at LPM. Single infection with H9, H7, and H5 subtypes was detected in 229 (31.5%), 27 (3.7%), and 25 (3.4%) of 726 AIV-positive specimens, respectively.	MNA was used as confirmatory test (titer ≥80 was considered positive). Seroconversion was defined as detection of a > four-fold rise in MN Ab titer between initial and a paired second serum sample, with the second sample achieving a titer ≥80.		
[94]	China (Beijing), 2013-2016	Large-scale CoPF, large-scale SF, poultry and swine SH, private PF and SF, backyard P and S raising sites / H9N2	No	Serum samples were tested by a turkey RBC-based HIA for Ab against A/environment/Beijing/w001/2013 H9N2. Both HI cut-off titers of 80 and 160 were considered. A four-fold or greater increase between paired sera with titers ≥ 40 for the second specimen was considered a new infection	No information.	During the study period, the seroprevalence trends did not increase or decrease among the 3 participant groups over time. However, PW had significantly higher risk of contracting H9N2 than the general population, but no statistically significant difference was identified between SW and general population.
[95]	China (districts of Guangzhou), 2015-2016	Large, small, and free-range PF, SH, wholesale- and retail-LPM / H9N2, H5 and H7 AIV	Yes. Samples from 17 poultry farms, 2 wholesale LPMs and 6 retail LPM were collected and tested by rRT-PCR using influenza virus H5, H7, H9. Overall, 1771 specimens	Human serum samples were tested by HIA for subtypes H5, H7 and H9. H9 samples with HI titer ≥40 were further confirmed by MN (titer ≥40 as endpoint for seropositivity). Both HIA and MNA were performed according to the WHO Manual for the Laboratory Diagnosis and Virological Surveillance of Influenza.	10.8% (32/296) of PW had a history of seasonal influenza vaccination, which was higher than the general population.	H9N2 was the dominant AIV virus subtype among PW, poultry and the environment around poultry facilities. The risks of H7N9 and H5N1 virus infection remain low in poultry workers, swine workers and the general populations in Beijing, China. **Compared to those working in farms, poultry workers in**

			including 317 bioaerosol samples, 991 environmental swabs from facility surface and 463 cloacal and/or oral swabs from poultry were taken. Overall, 424/1771 (23.9%) specimens were AIV positive, showing detection rates of 4.5%, 11.1%, 30.3% and 51.2%, in farms, transport vehicles, wholesale and retail LPMs respectively. H5, H7 and H9 AIV were detected from poultry and environmental samples with different detection rates: H9 14.6%, H5 2.9%, H7 1.0%.			**wholesale and retail LPMs had significantly higher risks of H9N2 infections** Statistical analysis showed that virus detection and transmission risk to human increased progressively along the poultry supply chain from farms, transport vehicles, wholesale LPM to retail LPM.
[96]	Pakistan, 2016-2017	WpE(L/D)P, SH, AI diagnostic Lab / H9N2	No	Serum samples were tested by a chicken RBC-based HIA for Ab against A(H9N2) (A/Turk/Wisc/1/66) AIV (positive titers ≥1:160 were considered positive).	Hand wash with water after each task was adopted by 74% (245/332) of the participants; 33% (108/332) of the participants used gloves, 39.5% (131/332) used consistently soap/sanitizer, 46% (154/332) outer	The obtained results demonstrated variable A(H9) seropositivity in poultry professionals: **lab staff had the highest seroprevalence (100%) and vets the lowest (38.5%).** The high seroprevalence values observed in lab staff and vaccinators might be explained by their exposure to viral cultures and influenza vaccines, respectively. Poultry

					garments/gowns, 49% (162/332) facemasks, 39.5% (131/332) shoe covers.	professionals with direct exposure to live Ag or infected birds or who did not use PPE had the highest A(H9) seropositivity.
[97]	Korea, 2016-2017	PF / HP H5N6	No	Serum samples were tested by MNA performed in accordance with WHO Guidelines (2011) for Ab against influenza A/duck/ES2 /Korea/2016 HPAI H5N6 virus (sera with MN titer > 1:80 or a titer increase greater than 4-fold in convalescent phase were considered a positive result).	Seasonal influenza vaccines were administered to 337/870 (38.7%) PFW before outbreak and to 534/870 (61.3%) PFW during the outbreak.	On the basis of these serological assays, no transmission of HPAI A/H5N6 to humans was identified in this studied cohort.
