# Supplementary material for: Occupational Risk from Avian Influenza Viruses at Different Ecological Interfaces Between 1997 and 2019
Source: Microorganisms. 2025 Jun 14;13(6):1391. doi: 10.3390/microorganisms13061391 (PMC12195780; doi:10.3390/microorganisms13061391)
Supplement: Supplementary file 1 [file microorganisms-13-01391-s001.zip › Table S6.pdf]

Table S6. Additional information related to Table S3 (Virological and serological results obtained from risk groups occupationally exposed to AIV). See Table S7 and Table S8 for acronym details. **In red bold font,** statistically higher occupational risk in workers.

Ref.	Country, Year(s)	Workplace (animal species exposure)/ Potential AIV exposure	Concurrent studies in animals and/or environments	Virological methods used in humans	Serological methods used in humans	Personal protective equipment (PPE), vaccines, antiviral therapy	Main outcomes
[98]	USA (IA), 2004, 2006	PF, PMePrP, WBH / AIV	No	74 sets of self-collected nasal and gargle swabs (obtained from 66 subjects who developed ILI during 24 months of follow-up) were tested by both culture in MDCK cells and R-Mix Fresh Cells™, and molecular methods (rRT-PCR and sequencing to first screen for IAV and then determine influenza H subtype).	Available serum samples from all the 869 enrolled participants were tested by HIA for Ab to 3 human influenza viruses A/New Caledonia⁄20⁄99(H1N1), A⁄Nanchang⁄933⁄95(H3N2), A⁄Panama⁄2007⁄99(H3N2), using a 0.65% guinea pig or 0.50% turkey RBC solution. Serum samples were also tested by MNA for Ab against LP A/Duck/Cz/1/56 (H4N8), A/Chucker/MN/14591-7/98 (H5N2), A/Turkey/MA/65 (H6N2), A/Turkey/VA/4529/02 (H7N2) and A/Turkey/MN/38391-6/95 (H9N2): sera were first screened at a dilution of 1:10, positive specimens were then titered in duplicate by examining twofold serial dilutions from 1:10 to 1:1280.	More than 50% of the participants were submitted to influenza vaccines during the 4 years prior to enrollment.	Obtained data suggest that hunting and exposure to poultry may be important risk factors for AIV infection among rural US populations.
[99]	China (Guangdong Province), 2006	Large LBM / HP H5N1	Yes. 94 cloacal swabs taken from live birds and 79 animal cage swabs were collected and tested by RT-PCR for H5, N1 and M genes. Positive PCR were confirmed by	Throat swabs collected from the infected patient were tested by RT-PCR for H5, N1 and M genes. Positive PCR results were confirmed by sequencing.	Serum samples obtained from purveyors at LBMs were analyzed by turkey RBC-based HIA for Ab against two different H5N1 virus strains (A/Hong Kong/486/97 and A/Vietnam/1194/04/H5N1) Neutralization test was used as confirmatory assay.	No information	The patient may have been infected with HPAI H5N1 virus by unknown mechanism at the food markets. The purveyor who tested HI positive slaughtered birds for 5 years (≈100 chickens/day) and did not notify any recent respiratory diseases.

			sequencing. None of 94 cloacal swabs was positive; 1/79 animal cage swabs (from a goose cage) resulted positive for HPAI H5N1.				
[100]	Nigeria, 2008-2011	Large-scale CoPF in AgA, open LBM / AIV including HP H5N1	No	Nasal and pharyngeal swabs collected as planned from 124 participants reporting ILI were screened by rRT-PCR for generic IAV and positive specimens further tested by rRT-PCR specific for avian H5, human H1, H3 subtypes, and 2009 pdm H1N1 viruses. Swab samples that resulted IAV positive/suspected positive, but could not be subtyped, were cultured in MDCK cells and passaged twice in order to amplify the virus for other studies.	Serum samples collected as planned were tested by MNA for Ab against AIV belonging to LP A/Migratory duck/Hong Kong MPS180/2003(H4N6), A/Nopi/Minnesota/07/462960-2(H5N2), A/Teal/Hong Kong/w312/97(H6N1), A/WF/Hong Kong/Mpb127/2005(H7N7), A/Migratory duck/Hong Kong/MP2553/04(H8N4), A/Migratory duck/Hong Kong/MPD268/2007(H10N4), A/Hong Kong/1073/1999(H9N2) (human origin), A/Chicken/New Jersey/15906-9/96(H11N1), A/DK/Alberta/60/76(H12N5), and HP A/CK/Nigeria/07/1132123(H5N1) subtypes. Sera were first screened at a dilution of 1:10 and positive specimens were then titrated (from 1:10 to 1:1280). HIA was also used to look for Ab against human AIV using a 0.65% (guinea pig) or 0.50% (turkey) solution of erythrocytes.	No information	Although cross-reactivity from Ab against other influenza viruses cannot be ruled out as a partial confounder, sero-epidemiological results indicate that some workers were exposed recently to H5N1 virus, providing evidence for subclinical HPAI H5N1 infections.
[101]	USA, 2009, 2010	Bird banding sites in WBH (wild ducks, wild geese, wild raptors) / AIV	No	Nasal, throat and conjunctival swabs from ILI subjects were tested by rRT-PCR for IAV screening and and subsequent reactions to determine	Sera collected from 127 BBa and 69 non-bird exposed controls were tested: by a Guinea pig RBC based HIA (titers >1:40 were considered positive) for Ab against human influenza virus A/Brisbane/10/2007(H3N2), and A/Brisbane/59/2007(H1N1); by MNA performed with MDCK cells for Ab against	Among BBa, 15% reported wearing gloves often or always, 36% used eye protection, 78% washed their	36% of BBa resulted also exposed to domestic chickens, ducks, or geese, and 24% to pigs. Despite reports of conjunctivitis and upper respiratory symptoms

				influenza HA subtype, including H5N1 and 2009 pdm H1N1.	A/GF/NJ/14190-23/96(H4N8), A/Nopi/Mn/07/462960-2(H5N2), A/CK/CA/32213-1/00(H6N2), A/BWTE/Ohio/07/495762-6(H7N3), A/Ty/CO/173105/02(H8N4), A/Ty/MN/38391-6/95(H9N2), A/Rhea/MA/44017-12/94(H10N4), A/CK/NJ/7290-2/95(H11N3) AIV.	hands often or always while working with animals. Controls were more likely vaccinated against human influenza in previous 5 years (76% vs. 53%).	while bird banding, sparse evidence of AIV infections was found in the US BBa.
[102]	Mongolia, 2009-2011	AgA (poultry, horses) / AIV including HP H5N1	No	Over 2 years of monthly follow-up, nasal and pharyngeal swabs collected from 100 ILI cases were tested by rRT-PCR specific for IAV detection; confirmed cases were further tested by rRT-PCR to detect concurrently human A/H1, human 2009 pdm H1N1, human A/H3, and avian A/H5 (Asian lineage) influenza viruses.	HA was used to detect Ab against the equine influenza virus A/Equine/Mongolia/01/2008(H3N8), and the following avian viruses: A/Migratory duck/Hong Kong MPS180/2003(H4N6) , A/Cygnus/Mongolia/3/2009(H5N1), A/Nopi/Minnesota/2007/462960-2(H5N2), A/Teal/Hong Kong/w312/97(H6N1), A/Water fowl/HongKong/Mpb127/2005(H7N7), A/Migratory duck/Hong Kong/MP2553/2004(H8N4), A/Migratory duck/Hong Kong/MPD268/2007(H10N4), A/Chicken/New Jersey/15906-9/1996(H11N1), A/Duck/Alberta/60/1976(H12N5) and the avian like A/Hong Kong/1073/1999(H9N2). Sera were first screened at a dilution of 1:10 and positive specimens (with MN titers ≥ 1:10) were further titered. A guinea pig or turkey RBC-based HIA was used according to standard procedures to test Ab against 3 human influenza viruses including the seasonal A/Brisbane/59/2007(H1N1) and A/Brisbane/10/2007(H3N2), and the H1N1 pdm-like influenza strains A/Mexico/4108/2009(H1N1).	No information	Seroreactivity against AIV and equine influenza virus among the study population was higher during the 12- and 24-months follow-up periods in comparison with seroreactivity at enrolment; no specific risk factor could be associated with it. AIV and equine influenza virus serologic results were likely confounded by cross-reacting Ab against human influenza viruses.

[103]	Australia, 2010	Large-scale CoCF, SH (chickens) / H10N7 from confirmed outbreak	Yes. Cloacal and tracheal swabs from 10 dead and 10 live birds were tested by IAV matrix gene quantitative rRT PCR and viruses were then subtyped by a microarray assay. AIV was isolated in embryonated chicken eggs and in MDCK cell cultures. Sequences from several viral genome segments confirmed the A(H10N7) virus as LP AIV. Serologic test was conducted by an IAV nucleoprotein–based blocking ELISA and a subtype H10–specific HIA showed a widespread infection with 18/20 samples positive. Sampling across 4 additional flocks on site	Conjunctival swabs collected from 6 workers and nose and throat swabs from all sampled workers were tested by an IAV rRT-PCR, and positive samples were further characterized by partial sequence analysis of the H gene.	Human serum samples were tested by HIA or VNA according to standard procedures.	No obvious breach of biosecurity occurred on the farm. SHW did not wear goggles and face masks in the evisceration section on the day the flocks were processed. because birds were assumed to be safe.	The phylogenetic analysis of the H sequence in chickens showed an unusual high degree of homology with North American AIV subtype H10. After the outbreak confirmation in chickens, the use of full PPE was implemented among SHW exposed to birds and carcasses from likely infected sites.

			showed that an additional 9/40 birds were positive for influenza A subtype H10.				
[104]	Indonesia, 2012-2016	LPM / HP H5N1	Yes. Cloacal swab samples from 226 sick or dead poultry were collected during May–Sep. 2013 (dry season) and Jan.-Feb. 2014 (rainy season), tested by virus isolation, and subtyped by RT-PCR. Overall, 31 isolates were identified as A(H5N1) Eurasian lineage viruses, 6 as A(H5N1) Indonesian lineage viruses, and 2 as H3N6 AIV.	Oropharyngeal swabs were tested by RT-PCR for viral RNA.	Serum samples from workers recruited in 2014 were tested by HIA (positive cut-off ≥32) to detect Ab to the HPAI virus A/turkey/East Java/v154/2013 (H5N1) of H5 clade 2.3.2.1 Eurasian lineage (Av154[H5N1 Eur]). For evaluation of the specificity, 2 additional viruses were used: A/chicken/East Java/Av240/2014 (H5N1) virus of H5 clade 2.1.3.3 Indonesian lineage and A/duck/East Java/ Av39/2013(H3N6) virus.	No information	The study gives evidence of high prevalence of avian A(H5N1) virus infection among LPM workers in 2013. Although a high percentage of workers had ILI, it could not be related to Av154(H5N1 Eur) virus infection. Authors asserted that Av154(H5N1 Eur) virus did not cause clinical symptoms in humans, indicating that the HP nature of AI A(H5N1) viruses was not inherited when transmitted to humans.
[105]	China (Zhejiang Province), 2013	Wholesale LPM, SH (chickens, ducks, geese, pigeons, wild birds, pigs, and others) / H7N9	No	Nasal swab samples from PW were done at the time of serum collection and tested by RT-PCR specific for the H of A(H7N9)	Serum samples were tested for Ab against a recombinant virus containing H and N from A/ Zhejiang/ DTID-ZJU01/13 (H7N9) by a turkey RBC-based HIA. Ab against the circulating 2009 influenza A(H1N1) virus A/Zhejiang/DTID-ZJU02/2009(H1N1) were also examined for reference. An HI titer of ≥80 was considered positive.	Seasonal flu vaccination was administered to 18/1129 (1.7%) individuals of the general population, 4/396 (1.4%)	The study revealed that 65.8% of A(H7N9) infected patients who survived (25/38) developed an HI antibody titer of ≥80 to A(H7N9) virus soon after symptom onset, whereas only 28.6% of patients

						PW, 2/45 (4.4%) patients.	who died (2/7) generated antibody titers of ≥80.
[106]	China, 2013	LBM with L/D-birds (chickens, ducks, pigeons, geese, quail, wild and pet birds, swine, dogs and cats) / H7N9	No	Virus isolation or nucleic acid detection by rRT-PCR were used to confirm positive cases.	Serological test was performed by a turkey RBC-based HIA with the A/Anhui/1/ 2013 (H7N9) virus used as Ag. Positive cases were confirmed if at least a four-fold or greater rise in Ab was detected by testing paired acute and convalescent sera.	No information	Most H7N9 confirmed cases were epidemiologically unrelated and recently exposed to poultry. Follow-up of contacts of these cases showed a low risk of secondary transmission of the virus, however a limited human to human transmission could not be excluded.
[107]	China (Zhejiang Province), 2013-2015	LPM, PF, BaPF, SH/PMePrP, WBH, WpE(L/D)P / H7N9	Yes. Environmental specimens were taken from 1488 poultry-related premises including 939 LPM (63.1%), 235 poultry rearing farms (15.8%), 173 backyard poultry farm areas (11.6%), 36 slaughtering and processing plants, (2.4%), 32 habitats for migratory birds (2.2%), 73 other premises (4.9%). Overall, 806/14,207 (6.1%) specimens tested positive by rRT-	Human respiratory specimens from patients with influenza were tested by rRT-PCR and viral isolation according to standard procedures.	Serum samples were collected from PW and tested by a horse RBC-based HIA for Ab against H7N9, according to WHO procedures.	No information	This study assessed the risk trend of H7N9 infection, showing that positive detection of H7N9 virus during environmental surveillance increased from the first to third wave of H7N9 infection ($P < 0.05$). **PW from concentrated areas of BaPF (7.7 %, 22/284) and PFW (3.7 %, 11/300) had higher positive rates for H7N9 antibody than those from LPMs (0.4 %, 1/227).** **No workers showing seropositivity to H7N9 were identified from SH/PMePrP (0/28), WBH (0/26) or other premises (0/47).**

			PCR aimed to identify H7N9 virus. Environmental samples positive for IAV nucleic acid were further typed as H7, H9 and N9.				
[108]	China (Guangzhou), 2013-2014	Retail and wholesale LPM, PF /H7N9	Yes. A total of 8900 samples including tracheal and cloacal swabs of live poultry, and environmental samples (drinking water, fecal droppings, cage floors) were collected from each stall. Out of 8900 samples, 131 (1.5%) tested positive by rRT-PCR for A(H7N9) virus and from 23 of them (17.6%) AIV were successfully isolated. 44.4% (16/36) samples from retail LPM and 50.0% (3/6) wholesale LPM tested rRT-PCR positive, whereas	Throat swabs from PW associated with the 19 contaminated LPM were tested by rRT-PCR to detect A(H7N9) virus. Positive samples were inoculated into the allantoic sac of 10-day-old pathogen-free embryonated chicken eggs for viral isolation.	To detect Ab against A/Guangzhou/1/2014(H7N9) virus, 316 serum samples were collected at the end of the seven-day medical observation and tested by a horse RBC-based HIA). HI titers >40 were considered positive.	The use of masks and gloves was reported only in 9.0% of PW from LPM. History of influenza A vaccination was reported from 2.1% of the workers.	All PW reported direct contact with poultry every day (especially slaughtering and de-feathering poultry), none of them attested development of respiratory symptoms during the past month. The extensively contaminated LPM were associated with few human infections with A(H7N9) viruses, accounting for a limited ability of this virus to cross species barrier.

			no sample from the 8 tested poultry farms was positive.				
[109]	China (Shenzen districts), 2013	Retail and wholesale PM / H7N9, H5N1	No	Nasal swabs, collected only in PW sampled in May 2013, were tested for H7N9 virus by rRT-PCR	Serum samples were tested by horse RBC-based HIA for Ab against A/Anhui/1/2013(H7N9) and A/Shenzhen/01/2011(H5N1) (titers >1:160 were considered as positive).	40.6% of PW enrolled in the 1st survey reported seasonal vaccination in the previous year. This information is lacking for the 2nd survey.	Although no participants had virologically confirmed H7N9 infection, the high proportion of PW with serologic evidence of H7N9 infection between May and Dec. 2013 suggests a substantial risk of mild H7N9 infections in the poultry-exposed group.
[110]	China (Zhejiang Province), 2014	LBM / H7N9	Yes. Environmental samples were collected from the LBM (A1), from 3 secondary wholesale markets (B1, C1, D1), and from a neighboring household where several chickens were bred. When tested by RT-PCR to detect IAV subtype H7 and N9, 4 swabs of chicken and duck eggs from A1 were H7N9 negative; 3/5 environmental samples from B1,	Respiratory tract samples collected from index case, cases 2 and 3 were tested by rRT-PCR to detect IAV subtype H7 and N9 and for the presence of seasonal influenza virus (H1, H3, and B) and of H5N1. Complete genomic fragments of the H7N9 virus were amplified directly from clinical samples, and sequencing was done. Respiratory samples from the 25 close contacts were also examined.	Serum samples from the 25 close contacts were collected ≥3–4 weeks after exposure to a case and tested by horse RBC based HIA and MNA (titer ≥1:40 and 4-fold or greater rise in titer in paired sera were considered positive). The antigen used in HI assay was A/Zhejiang/1/2013(H7N9).	PPE usage was limited to Case 2 visiting to Index case and to Case 3 visiting to Case 2. None of the 25 close contacts used PPE at any time before illnesses onset to the time of isolation of the case in hospital.	This family cluster is compatible with non-sustained person-to-person transmission of H7N9. Index case died; case 2 and 3 completely recovered. Index case was infected from LBM and secondary cases by index case during unprotected exposure. None of the 25 close contacts developed symptoms during 7-days surveillance.

			1/2 sewage samples from C1, 10/20 environmental samples from D1 were positive for H7N9. 12 environmental samples from the area where neighbors were breeding poultry were all H7N9 negative; 11/26 environmental samples from different LBM were H7N9 positive during Jan. 2014.				
[111]	China (Jilin Province), 2014	Small-scale CoPF / H7N9, H9N2	Yes. Active virological surveillance was performed by sampling poultry and environment of the patient's poultry farm and other 3 epidemiologically linked farms. From 27 IAV positive samples, 9 isolates were obtained in patient's farm only: 6 H7N9 from oropharyngeal or	Throat swabs, collected from the patient and close contacts who developed clinical symptoms, were tested by rRT-PCR for seasonal influenza viruses (H1, H3 or B) and AIV (H1 to H16 and N1 to N9 subtypes).	Serum samples, collected in the acute and convalescent phases from the patient's close contacts, were tested by a horse RBC-based HIA, with a modified order of serum treatment procedures, to detect Ab response to A/Anhui/1/2013 H7N9 reference virus.	The H7N9 positive PFW checked the farm daily without PPE	The continued introduction of different species of poultry and the mixed breeding model may have contributed to the diversified genotypes generated by co-circulation of the H7N9 and H9N2 viruses in the patient's poultry farm.

			cloacal swabs of chickens and 3 H9N2 from oropharyngeal swabs of chickens and drinking water.				
[112]	Russia (Russian Far East, Eastern Siberia, Western Siberia, Ural and near regions, European part of Russia), 2014-2015	PF / H7N9, HP H5N1 and H5N8 AIV	No	Pieces of the bronchi, trachea and lungs from 19 individuals who died (influenza suspected cause) and clinical materials from 25 individuals with severe disease were collected and processed for virus isolation in Madin-Darby Canine Kidney (MDCK) cell culture.	The presence of Ab to different types/serotypes of influenza viruses in PFW sera was tested by HIA following a WHO standard procedure. Ag used in HIA included the A/California/07/09(H1N1)pdm09, A/Texas/50/2012(H3N2), B/Massachusetts/2/2012 (Yamagata lineage) and B/Brisbane/60/2008 (Victoria lineage) human influenza viruses, and the A/Anhui/01/2013(H7N9) AIV and HPAIV A/Black-Headed gull/Tyva/115/09 (H5N1) and A/wigeon/Sakha/1/2014 (H5N8). None of the 1939 PFW serum samples produced positive results with the H5N1, H5N8, H7N9 antigens even at dilution 1:10.	No information	This study aimed to characterize herd immunity of the population from Asian part of Russia before influenza epidemic and to describe influenza viruses isolated from severe cases including cases with fatal outcomes in the 2014-2015 epidemic season. HI test enabled to study 3888 serum samples from healthy individuals including 1939 samples collected from poultry farm workers. None of the 3888 samples produced positive results with the antigens A/H5N1, A(H5N8) and A/H7N9.
[113]	Cameroon, 2016-2017	PF, LBM with (L/D)P / HP H5N1	Yes. During the outbreak period, cloacal and tracheal swabs from birds and post-mortem organ biopsies were examined	Nasopharyngeal and oropharyngeal swab samples were tested for influenza A and B viruses by RT-qPCR. Positive samples for IAV were then tested by RT-qPCR for	Serum samples from 131 selected subjects (in total 262 paired sera) were tested by horse and human RBC-based HIA, against HPAI H5N1 viruses including the two egg isolates from Cameroon, reference clade 2.3.2.1 A(H5N1) viruses/antigens A/common magpie/Hong Kong/5052/2007, A/environment/Cambodia/z2EP1e3W7M1/2015 and A/duck/Vietnam/NCVD-1584/2012.	No information	During the 2016 outbreak of AI A(H5N1) among poultry, no active AIV infections were detected in PFW and LBMW. Serological results suggested that exposure to AI likely occurred through contact with

			for the detection of IAV as well H5 and H7 subtypes. 58/147 poultry samples tested IAV positive, 34 of whom confirmed as HPAIV A(H5N1)	A(H3N2), A(H1N1)pdm09, A(H5N1), and A(H7N9).	HIA positivity was confirmed by MNA. Samples with detectable Ab in the second serological draw (HI titers≥ 10) were then re-tested by HIA and MNA utilizing both the 1st and 2nd serum samples. Exposure to these viruses was considered "suspected" with an HIA titer≥ 40 in the 2nd serum sample and/or seroconversion defined as the detection of Ab above the thresholds (four-fold increase) defined following no detection of Ab in the serum sample from the previous period.		diseased or dead poultry. No PW reported any symptoms in relation to acute HPAIV infection, thus seroconversion could be related to subclinical or very mild cases.
