# Supplementary material for: Occupational Risk from Avian Influenza Viruses at Different Ecological Interfaces Between 1997 and 2019
Source: Microorganisms. 2025 Jun 14;13(6):1391. doi: 10.3390/microorganisms13061391 (PMC12195780; doi:10.3390/microorganisms13061391)
Supplement: Supplementary file 1 [file microorganisms-13-01391-s001.zip › Table S7.pdf]

Table S7. Ecological interfaces posing zoonotic occupational risk: workplaces and risk group acronyms used in Table S1, Table S2 and Table S3.

Acronym (A-L)	Detail		Acronym (O-W)	Detail
**AgA**	agricultural area(s)		**OF**	ostrich farm(s)
**AgW**	agricultural worker(s)		**OW**	other worker(s)
**AnFW**	animal farm worker(s)		**PCu**	poultry culler(s)
**BBa**	bird bander(s)		**PDeW**	poultry depopulation worker(s)
**BHa**	bird handler(s)		**P-E**	poultry exposed
**BHu**	bird hunter(s)		**PF**	poultry farm(s)
**BaDF**	backyard duck farm(s)		**PFW**	poultry farm worker(s)
**BaPF**	backyard poultry farm(s)		**PG**	poultry grower(s)
**BaPFW**	backyard poultry farm workers		**PIn**	poultry industry(s)
**BaPG**	backyard poultry grower(s)		**PInW**	poultry industry worker(s)
**BaTG**	backyard turkey grower(s)		**PM**	poultry market(s)
**CoCF**	commercial cicken farm(s)		**PMW**	poultry market worker(s)
**CoCFW**	commercial chicken farm worker(s)		**PMePr**	poultry meat processor(s)
**CoDF**	commercial duck farm(s)		**PMePrP**	poultry meat processing plant(s)
**CoPF**	commercial poultry farm(s)		**P-NE**	poultry not exposed
**CoPFW**	commercial poultry farm workers(s)		**PW**	poultry worker(s)
**CoPG**	commercial poultry grower(s)		**RSBHu**	rural subsistence bird hunter(s)
**CoSF**	commercial swine farm		**SF**	swine farm(s)
**CoSFW**	commercial swine farm worker(s)		**SFW**	swine worker(s)
**DFW**	duck farm worker(s)		**SH**	slaughterhouse(s)
**DeW**	depopulation worker(s)		**SHW**	slaughterhouse worker(s)
**DHu**	duck hunter(s);		**TFW**	turkey farm worker(s)
**FaM**	family member(s)		**TMePr**	turkey meat processor(s)
**FW**	farm worker(s)		**USpHu**	urban sport hunter(s)
**GoPDeW**	government poultry depopulation worker(s)		**Vet**	veterinarian(s)
**GoW**	government worker(s)		**WBH**	wild bird habitat
**HCW**	healthcare worker(s)		**WBHW**	wild bird habitat worker(s)
**InPF**	industrial poultry farm(s)		**WBHu**	wild bird hunter(s)
**InPFW**	industrial poultry farm worker(s)		**WLB**	wild-life biologist(s)
**InTFW**	industrial turkey farm worker(s)		**WLE**	wild-life exposed
**Lab**	laboratory		**WLE-GoW**	wild-life exposed government worker(s)
**LBM**	live bird market(s)		**WLE-Vet**	wild-life exposed veterinarian(s)
**LBMW**	live bird market worker(s)		**WLH**	wild-life habitat
**LPM**	live poultry market(s)		**WM**	wet market(s)
**LPMW**	live poultry market worker(s)		**WMW**	wet market worker(s)
**LPV**	live poultry vendor(s)		**WpE(L/D)P**	workplace(s) of exposure to (Live/Dead) poultry
**LW**	laboratory worker(s)		**WpE(L/D)PW**	workplace(s) of exposure to (Live/Dead) poultry worker(s)
