# Supplementary material for: Occupational Risk from Avian Influenza Viruses at Different Ecological Interfaces Between 1997 and 2019
Source: Microorganisms. 2025 Jun 14;13(6):1391. doi: 10.3390/microorganisms13061391 (PMC12195780; doi:10.3390/microorganisms13061391)
Supplement: Supplementary file 1 [file microorganisms-13-01391-s001.zip › Table S8.pdf]

Table S8. Ecological interfaces posing zoonotic occupational risk: list of additional acronyms used in Table S1, Table S2 and Table S3.

Acronym (A-M)	Detail	Acronym (N-W)	Detail
**Ab**	antibody(ies)	**NIA**	neuraminidase inhibition assay(s)
**Ag**	antigen(s)	**np.sw.**	nasopharynx. swab(s)
**AI**	avian influenza	**n.sw.**	nasal swab(s)
**AIV**	avian influenza virus(es)	**NT**	neutralization test(s)
**c.sw.**	conjunctival swab(s)	**op.sw.**	oropharynx swab(s)
**c**	control subjects	**PCR**	polymerase chain reaction
**e.sw.**	eye swab(s)	**pdm**	pandemic(s)
**ELISA**	enzyme-linked immunosorbent assay	**PNA**	plaque neutralization assay(s)
**GMT**	geometric mean titer(s)	**PPE**	personal protective equipment
**HA**	hemagglutinin(s)	**p.sw**	pharyngeal swab(s)
**HAA**	hemagglutination assay(s)	**RBC**	red blood cells
**HI**	hemagglutination inhibition	**RT-PCR**	reverse transcriptase-PCR
**HIA**	hemagglutination inhibition assay(s)	**rRT-PCR**	real-time RT PCR
**HP**	high pathogenicity	**s.c.**	seroconversion
**HPAI**	high pathogenicity avian influenza	**SRH**	single radial haemolysis assay(s)
**HPAIV**	HP avian influenza virus(es)	**s.sw.**	sputum swab(s)
**HS**	hospitalized subjects	**t.sw.**	throat swab
**IAV**	influenza A virus(es)	**VMN**	virus microneutralization
**IFA**	immunofluorescence assay(s)	**VI-CC**	virus isolation in Cell culture
**ILI**	influenza like illness(es)	**VI-EE**	virus isolation in embryonated egg
**LP**	low pathogenicity	**VN**	virus neutralization
**LPAI**	low pathogenicity avian influenza	**VNA**	virus neutralization assay(s)
**LPAIV**	LP avian influenza virus(es)	**WB**	western blot
**MDCK**	Madin-Darby canine kidney	**WBA**	western blot assay(s)
**MN**	microneutralization	**WHO**	World Health Organization
**MNA**	microneutralization assay(s)		
